# Recruited Monocyte-Derived Macrophages Drive T Cell Inflammation in Immune Checkpoint Inhibitor-Mediated Pneumonitis

**DOI:** 10.34133/cancomm.0049

**Published:** 2026-09-02

**Authors:** Xiaoran Cui, Renyong Zhi, Xiaoyan Li, Yinming Ji, Zhisong Hu, Pengfei Cui, Yuhui Qin, Tao Li, Liangliang Wu, Lingxiong Wang, Jinfeng Li, Yibing Bai, Hui Li, Tianyi Liu, Yi Hu

**Affiliations:** ^1^ Senior Department of Oncology, the Fifth Medical Center of Chinese PLA General Hospital, Beijing, P. R. China.; ^2^ Institute of Oncology, the Fifth Medical Center of Chinese PLA General Hospital, Beijing, P. R. China.; ^3^ Department of Hematology, Beijing Friendship Hospital, Capital Medical University, Beijing, P. R. China.; ^4^ Department of Oncology, the Second Affiliated Hospital of Anhui Medical University, Hefei, Anhui, P. R. China.; ^5^ Department of Oncology, the Second Medical Center of Chinese PLA General Hospital, Beijing, P. R. China.; ^6^ Department of Gastroenterology, Beijing Friendship Hospital, Capital Medical University, Beijing, P. R. China.; ^7^ Department of Oncology, the First Medical Center of Chinese PLA General Hospital, Beijing, P. R. China.; ^8^ Institute of Oncology, the First Medical Center of Chinese PLA General Hospital, Beijing, P. R. China.

## Abstract

**Background:** Immune checkpoint inhibitor-mediated pneumonitis (CIP) constitutes a major toxicity that limits the clinical application of cancer immunotherapy, whereas its underlying mechanisms remain incompletely understood. Current management relies on nonspecific immunosuppressants, lacking precision therapies. Although single-cell RNA sequencing (scRNA-seq) of bronchoalveolar lavage fluid (BALF) has implicated T cell activation and inflammatory myeloid responses in CIP pathogenesis, critical gaps persist regarding interstitial lung immunity and mechanisms governing monocyte/macrophage–T cell co-enrichment and crosstalk. We aimed to delineate the lung immune circuits that drive CIP and to identify targetable monocyte/macrophage–T cell pathways that could be leveraged for precision intervention. **Methods:** To elucidate CIP pathogenesis, we performed integrated scRNA-seq analysis of BALF from CIP^+^ and CIP^−^ patients with validation in a prospective cohort using flow cytometry, enzyme-linked immunosorbent assay, Western blotting, and quantitative polymerase chain reaction. Mechanistic studies were performed using an established tumor-bearing forkhead box P3–diphtheria toxin receptor–green fluorescent protein (*Foxp3-DTR-GFP*) mouse model of programmed death-1 inhibitor-induced CIP via micro-computed tomography, histopathology, scRNA-seq, flow cytometry, multiplex immunofluorescence, Western blotting, Transwell migration assays, and pharmacologic interventions. **Results:** In CIP^+^ patient BALF and mouse lung tissues, CD8^+^ T cells expressing cytotoxic effectors and C-X-C chemokine receptor 3 (CXCR3) expanded concomitantly with distinct C-C chemokine receptor 2 (CCR2)^+^ monocyte-derived macrophages (MoMΦ) exhibiting a highly inflammatory phenotype, while tissue-resident macrophages were markedly reduced. Mouse models revealed that expanded CCR2^+^ MoMΦ originating from circulation replenished the depleted niche of lung-resident interstitial macrophages. Mechanistically, integrated in silico prediction and experimental validation demonstrated that CCR2^+^ MoMΦ recruited CD8^+^ T cells via the C-X-C motif chemokine ligand 9/10 (CXCL9/10)–CXCR3 axis. Conversely, CD8^+^ T cells drove CCR2^+^ MoMΦ expansion and pro-inflammatory phenotype via the interferon-γ (IFN-γ) axis, suggesting the existence of a positive feedback loop between these cell types. Pharmacological targeting of CCR2/CCR5 or CXCR3 signaling attenuated pneumonitis, reduced pulmonary CCR2^+^ MoMΦ infiltration, diminished pathogenic T cell activation and cytotoxicity, and improved survival without compromising antitumor immunity. **Conclusions:** Our findings establish CCR2^+^ MoMΦ and the IFN-γ–CXCL9/10–CXCR3 axis as core drivers of CIP pathogenesis and validate their therapeutic targeting potential. This work provides a scientific foundation for developing CIP-specific prevention and treatment strategies.

## Background

Immune checkpoint inhibitors (ICIs) targeting programmed death-1/programmed death-ligand 1 (PD-1/PD-L1) and cytotoxic T lymphocyte-associated antigen-4 (CTLA-4) enhance T cell antitumor activity by blocking immunosuppressive signals, significantly prolonging survival in patients with various cancers. However, excessive immune system activation can lead to autoimmune damage against healthy tissues, termed immune-related adverse events (irAEs) [1–4]. Among these, immune checkpoint inhibitor-mediated pneumonitis (CIP) is the most common cause of ICI-related mortality in patients receiving anti-PD-1/PD-L1 therapy, with a mortality rate of approximately 35% [5,6] and real-world incidence in non-small cell lung cancer (NSCLC) cohorts reaching up to 20% [7,8]. The core clinical challenge in CIP management lies in the lack of predictive biomarkers and specific therapeutic targets. Systemic corticosteroids remain the mainstay of treatment [9], yet a substantial proportion of patients are steroid-refractory, and prolonged immunosuppression may compromise antitumor efficacy. Previous studies have established that CIP is characterized by the accumulation and activation of CD4^+^ and CD8^+^ T cells [10–12]. Notably, aberrant T cell activation can occur even after ICI discontinuation, as delayed-onset CIP has been reported months after stopping therapy [6,13], suggesting that drug-independent, self-sustaining immune circuits may contribute to disease pathogenesis.

Tissue-resident alveolar and interstitial macrophages (IMs), along with recruited monocyte-derived macrophages (MoMΦ), play crucial roles in innate immunity and maintaining lung homeostasis. Upon perturbation of lung homeostasis, tissue-resident macrophages are often replaced by MoMΦ [14–16]. However, excessive MoMΦ infiltration has been demonstrated to closely associate with diverse inflammatory and infectious lung injuries [17–19]. In patients with CIP, bronchoalveolar lavage fluid (BALF) analysis revealed a significant increase in the proportion of pro-inflammatory monocytes/macrophages derived from circulation, accompanied by a marked reduction in tissue-resident alveolar macrophages (AMs) [20,21]. The C-C motif chemokine ligand 2–C-C motif chemokine receptor 2 (CCL2–CCR2) axis has been reported as a core pathway driving intrapulmonary MoMΦ infiltration. During lung tissue injury, activation of this axis recruited classical CCR2^+^ monocytes to sites of inflammation. These monocytes then differentiated into pro-inflammatory MoMΦ, which further amplified the inflammatory cascade by producing multiple inflammatory mediators [15,22,23]. Notably, clinical molecular imaging has demonstrated specific accumulation of CCR2^+^ macrophages within fibrotic foci of idiopathic pulmonary fibrosis patients and mouse models. Their density exhibited a positive correlation with the degree of fibrosis and radiotracer uptake, supporting their utility as molecular biomarkers for monitoring disease activity and therapeutic responses [24]. Moreover, in experimental bleomycin-induced pulmonary fibrosis, silencing CCR2 attenuated disease severity [25]. Previous research on CIP pathogenesis has primarily focused on T cells. How monocytes/macrophages are transcriptionally reprogrammed in CIP and how they interact with activated T cells within the lung interstitium remain poorly understood.

To address these gaps, we sought to elucidate the contribution of MoMΦ and their crosstalk with T cells to CIP pathogenesis and to explore whether these interactions could be therapeutically targeted while preserving antitumor immunity. We therefore adopted the previously validated forkhead box P3–diphtheria toxin receptor–green fluorescent protein (*Foxp3-DTR-GFP*) mouse model, which has been shown to be suitable for assessing irAEs [26,27]. In parallel with BALF single-cell RNA sequencing (scRNA-seq) data from patients with and without CIP, we designed a multimodal study integrating scRNA-seq of mouse lung tissue, multiparameter flow cytometry, multiplex immunofluorescence, in vitro migration assays, and in vivo pharmacologic interventions. We hypothesized that a CCR2-dependent MoMΦ compartment and CD8^+^ T cells form a pathogenic inflammatory circuit in CIP and that chemokine axes such as CCL2–CCR2 and C-X-C motif chemokine ligand 9/10–C-X-C chemokine receptor 3 (CXCL9/10–CXCR3) represent tractable targets for interrupting this circuit.

## Materials and Methods

### Integration of scRNA-seq data from BALF of patients

To elucidate transcriptional features of monocytes and macrophages, we obtained scRNA-seq data from BALF cells of CIP^+^ and CIP^−^ patients deposited in the Genome Sequence Archive Human database (accession nos. OMIX001006 and OMIX004420) [20] and the European Genome-Phenome Archive database (accession no. EGAD00001009723) [21]. Filtered gene-cell count matrices generated by Cell Ranger (version 4.0, 10x Genomics) and featureCounts pipelines served as input for batch correction. Each dataset underwent preprocessing in the Seurat R package (version 5.0) [28], including logarithmic transformation and scaling. The top 2,000 highly variable genes were identified per dataset using the FindVariableGenes function. These processed datasets and highly variable genes were integrated via Harmony (version 1.0) [29] for batch correction. Uniform Manifold Approximation and Projection (UMAP) visualizations were subsequently generated from the harmonized low-dimensional embeddings. The demographic and disease characteristics of the patients included in the scRNA-seq analysis were summarized in Table S1.

### Patient cohort, CIP diagnosis, and BALF collection

To validate the key immune cell and gene expression changes identified by scRNA-seq, BALF samples were prospectively collected by bronchoscopy from an independent validation cohort of 11 patients with NSCLC receiving ICI therapy at the Chinese PLA General Hospital. This validation cohort included 7 CIP^+^ patients and 4 CIP^−^ patients. Demographic and disease characteristics of the validation cohort were summarized in Tables S2 and S3.

CIP was diagnosed by a characteristic combination of clinical features and chest imaging abnormalities [30]. All patients developed new or progressively worsening respiratory symptoms (e.g., dyspnea, reduced exercise tolerance, exertional desaturation, and/or cough) together with new or progressive pulmonary infiltrates on high-resolution computed tomography (CT) during ICI treatment. All suspected cases underwent bronchoscopy with BALF collection to complete an infectious work-up. CIP was considered only when BALF cultures for bacteria, fungi, and mycobacteria and respiratory viral testing were negative, and when there was no clear clinical evidence of infection. In parallel, other causes of pneumonitis were excluded by reassessment of tumor burden according to the Response Evaluation Criteria in Solid Tumors (RECIST 1.1) and by clinical evaluation for alternative etiologies, including tumor progression, radiation-induced pneumonitis, and heart failure. The final diagnosis of CIP was proposed by the treating medical oncologist and adjudicated by a multidisciplinary team, including at least 2 medical oncologists and one thoracic radiologist, after review of the clinical course, imaging, and microbiological data. CIP severity was graded according to the National Cancer Institute Common Terminology Criteria for Adverse Events (CTCAE, version 4.03), and radiological patterns of CIP were classified following the criteria described by Naidoo et al. [31].

BALF samples were collected before the initiation of systemic corticosteroids. In patients with CIP, bronchoalveolar lavage was performed in the lung lobe or segment showing new or most prominent infiltrates, avoiding direct tumor involvement. CIP^−^ patients underwent bronchoscopy because CIP was initially suspected during ICI treatment, but they were ultimately adjudicated as having tumor progression rather than CIP. For these CIP^−^ patients, BALF was collected from an unaffected lung lobe or segment without tumor involvement.

### Cell lines

Human embryonic kidney 293T cells (CRL-3216), mouse Lewis lung carcinoma LLC1 cells (CRL-1642), and mouse melanoma B16-F10 cells (CRL-6475) were obtained from the American Type Culture Collection. Cell lines were maintained at 37 °C in a humidified atmosphere containing 5% CO_2_ in Dulbecco’s modified Eagle’s medium (DMEM; Gibco, Thermo Fisher Scientific) supplemented with 10% fetal bovine serum (FBS; Gibco, Thermo Fisher Scientific) and 1% penicillin–streptomycin (Gibco, Thermo Fisher Scientific). Cells were passaged every 1 to 2 d, or when reaching 70% to 90% confluence, to maintain logarithmic growth. All cell lines were authenticated by short tandem repeat (STR) profiling and confirmed to be free of mycoplasma contamination before use.

### Establishment of LLC1-Luc and B16-F10-Luc cells

Lentiviral particles carrying the firefly luciferase gene were generated by cotransfecting 293T cells in 10-cm dishes with the luciferase lentiviral vector and the packaging plasmids psPAX2 and pMD2.G using polyethylenimine (PEI; #246743, Sigma-Aldrich) according to the manufacturer’s protocol. Viral supernatants were collected at 48 and 72 h post-transfection, pooled, and used to transduce LLC1 cells and B16-F10 cells. Stable polyclonal populations were selected with puromycin (P8833, Sigma-Aldrich) to eliminate uninfected cells.

### Mouse model

Inbred C57BL/6 *Foxp3-DTR-GFP* mice (Shanghai Model Organisms) and homozygous *Ccr2-CreER* mice, originally generated in our laboratory as previously described [32], were housed in the Animal Facility of the Chinese People’s Liberation Army General Hospital under specific pathogen-free conditions (12-h light/dark cycle, standard chow and water ad libitum). *Ccr2-CreER* mice were crossed with homozygous *Rosa26-tdTomato reporter* mice (Shanghai Model Organisms) to generate *Ccr2^ertcre^Rosa^tdTomato^* mice for CCR2 lineage-tracing experiments. All experiments were performed with 8- to 12-week-old mice and were approved by the Laboratory Animal Welfare Ethics Committee of the Chinese People’s Liberation Army General Hospital (ethics approval: 2023-X19-105).

### Experimental tumor models and CIP induction

C57BL/6 *Foxp3-DTR-GFP* mice received subcutaneous injections of 8 × 10^5^ LLC1-Luc or B16-F10-Luc tumor cells. Tumor growth was monitored by caliper measurements. Treatment began when the mean tumor size reached 40 to 50 mm^2^; mice were euthanized upon reaching 150 mm^2^. To induce CIP, mice were treated with intraperitoneal injections of 300 ng of diphtheria toxin (DT; D0564, Sigma-Aldrich) in phosphate-buffered saline (PBS) to deplete regulatory T (Treg) cells, followed by 250 μg/mouse of either control immunoglobulin G2a (IgG2a) (2A3) or anti-PD-1 antibody (RMP1-14, BE0146, Bio X Cell). Lung inflammatory lesions were assessed using micro-CT and histopathological examination. Humane endpoints were predefined, and mice were euthanized earlier if they met any endpoint criteria, including body weight loss > 20%, severe respiratory distress, inability to ambulate or access food/water, persistent lethargy/hypothermia, or tumor ulceration. Euthanasia was performed by CO_2_ inhalation followed by cervical dislocation to confirm death.

### Single-cell suspension preparation of mouse lung tissue

Lung tissue from *Foxp3-DTR-GFP* mice was minced with surgical scissors and transferred into 15-ml conical tubes containing DMEM supplemented with collagenase IV (C5138, Sigma-Aldrich) and deoxyribonuclease (DNase) I (EN401, Vazyme), followed by incubation at 37 °C with agitation for 20 min. The digestion was terminated by adding PBS buffer containing 2% FBS. The cell suspension was filtered through a 70-μm cell strainer into a 50-ml conical tube and centrifuged at 400*g* for 10 min at 4 °C. The supernatant was carefully removed, and the pellet was resuspended in 5 ml of red blood cell lysis buffer (#420302, BioLegend). The suspension was incubated on ice for 5 min, followed by the addition of 20 ml of PBS to terminate the reaction. The mixture was centrifuged under the aforementioned conditions, and the supernatant was discarded. The pellet was resuspended in 5 ml of Cell Staining Buffer (#420201, BioLegend) and then recentrifuged under identical parameters. After supernatant removal, the pellet was resuspended in 500 μl of Cell Staining Buffer and stained with 5 μl of 7-aminoactinomycin D (7-AAD) Viability Dye (#420403, BioLegend) for 5 min. 7-AAD-negative cells were isolated via flow cytometry sorting and collected in cell resuspension buffer. The sorted cell suspension was centrifuged under the same conditions and resuspended in fresh buffer to achieve a target concentration of 1,000 cells/μl as determined by a hemocytometer.

### Mouse lung tissue scRNA-seq library preparation, preprocessing, and cell annotation

For each sample, 12,000 cells were transferred to the Next GEM Chip (10x Genomics) according to the manufacturer’s instructions. scRNA-seq libraries were prepared using the Chromium Single Cell V(D)J 5’ Reagent Kit v2 (10x Genomics). Raw sequencing data were processed using Cell Ranger, including demultiplexing of FASTQ reads, alignment to the mouse mm10 reference genome, and quantification of unique molecular identifier (UMI) counts. Preprocessing and analysis of the UMI count matrix were performed using Seurat [28]. As part of the quality control steps, the following cells were filtered out: (a) doublets detected by the Scrublet package (version 0.2.1); (b) cells with UMI counts <500 or >50,000; (c) cells exhibiting mitochondrial gene content >5%; (d) cells with ribosomal gene content < 20%; and (e) cells with detected genes <200 or >5,000. We integrated the remaining high-quality singlet gene expression matrices using the JointLayer function provided by Seurat. Variably expressed genes with mean expression between 0.0125 and 3 and quantile-normalized variance greater than 0.5 were selected using Seurat and then used to compute the principal components (PCs). A cluster of significant PCs was selected using the JackStraw and PCElbowPlot functions of Seurat. Cell clustering and UMAP visualization were performed using the FindClusters and RunUMAP functions, respectively. The annotations of cell identity on each cluster were applied by combining automated reference-based mapping with manual marker-guided refinement, followed by independent validation using Azimuth [33].

### Downstream bioinformatics analysis of human and mouse scRNA-seq data

#### Pathway enrichment analysis

Differential expression analysis was performed between groups or clusters as indicated. Differentially expressed genes (DEGs) were defined as |log2FoldChange| > 1 with adjusted *P* value < 0.05 in monocyte/macrophage, natural killer (NK) cell, and T cell clusters. Gene Ontology (GO) and Kyoto Encyclopedia of Genes and Genomes (KEGG) pathway analyses were performed using the ClusterProfiler R package (version 4.1.0) [34]. The Gene Set Variation Analysis (GSVA) R package (version 1.50.5) [35] was used to calculate expression scores for gene sets.

#### Trajectory analysis

Cell trajectory analysis was performed using Palantir (version 1.3.6) [36] to model differentiation processes and identify branch points in single-cell transcriptomic data. The analysis utilized a raw count gene expression matrix as input. Dimensionality reduction was first implemented through diffusion maps within Palantir, followed by manual specification of the trajectory’s start cell. Pseudotime values and branch probabilities for each cell were then computed, employing manifold learning to reconstruct differentiation trajectories and Markov chain-based random walks to simulate terminal state probabilities. Terminal states were identified by clustering endpoints of diffusion map components and selecting clusters exhibiting high expression of terminal marker genes, while branch points (defined as trajectory bifurcations) were automatically detected by the algorithm. Differentiation potential was quantified as entropy derived from branch probability distributions, with higher entropy indicating greater developmental plasticity. Results were visualized by mapping pseudotime values and branch probabilities onto a 2-dimensional UMAP embedding. To characterize CCR2 and CCL2 changes along the inferred trajectory, their expression patterns were evaluated across pseudotime using log-normalized expression values and smoothed trend curves, and results were visualized by mapping pseudotime, branch probabilities, differentiation potential, and gene trends onto 2-dimensional UMAP embeddings.

#### Transcription factor analysis

Gene regulatory network inference and regulon analysis were performed using Python implementation of Single-Cell Regulatory Network Inference and Clustering (PySCENIC; version 0.12.1) [37]. The input consisted of a quality-controlled and normalized scRNA-seq count matrix formatted as an AnnData object. Initially, coexpression modules were inferred using GRNBoost2 [37], a gradient boosting machine-based method, to identify potential transcription factor (TF)–target gene relationships. These modules were subsequently refined into regulons (representing direct TF target genes) via cisTarget [37], leveraging motif enrichment analysis with species-specific databases (human or mouse). Enrichment was assessed for the top 5,000 motifs within genomic regions spanning 500 base pairs, 5 kilobases, and 10 kilobases around transcription start sites. Regulons were retained based on a normalized enrichment score > 3.0 and an area under the curve (AUC) recovery curve threshold of 0.05. The activity of each regulon within individual cells was then quantified using AUCell [37], which calculates the area under the recovery curve for the regulon’s target genes, with the AUC threshold for binarizing activity determined automatically per regulon. The resulting regulon activity matrix (AUC scores) served as the basis for downstream analyses, including dimensionality reduction (e.g., UMAP) and clustering. Additionally, regulon specificity scores were computed to identify cell type-specific regulons.

#### Intercellular communication network analysis

Intercellular communication analysis was performed using the CellChat R package (version 2.1.2) [38] to quantitatively infer signaling networks from scRNA-seq data. For human data, the normalized expression matrix of macrophages and T cells was processed through its integrated biological-statistical framework. For mouse data, the normalized expression matrix of monocytes/macrophages and T cells was used. Leveraging a curated database of ligand–receptor–cofactor interactions representing known heteromeric complexes, CellChat identified major signaling inputs/outputs and characterized functional cellular coordination via network analysis and pattern recognition.

### In vivo micro-CT system

In vivo pulmonary micro-CT scans were performed on live mice using the PerkinElmer Quantum GX system under approved protocols. Mice were anesthetized with 2% isoflurane, with physiological parameters monitored (respiratory rate: 80 to 120 beats per minute; temperature: 37 ± 0.5 °C). Respiratory-gated imaging at the expiratory phase was acquired at 90-kVp, 88-μA, 72-μm isotropic resolution (cumulative dose < 200 mGy). Three-dimensional reconstructions were generated from motion-corrected raw data using iterative algorithms (PerkinElmer Analyze 12.0, IVIS-Lumina III module).

### In vivo bioluminescence measurement

Tumor growth was monitored using in vivo bioluminescence imaging with the PerkinElmer In Vivo Imaging System (IVIS) Spectrum system. Prior to imaging, mice received an intraperitoneal injection of 200 μl of d-luciferin (15 mg/ml in PBS, L2916, Thermo Fisher Scientific). Tumor bioluminescent signals were quantified as total flux (photons/s) using Living Image software (version 3.1.0, Caliper Life Sciences).

### Histology

Mouse lung samples were perfusion-fixed overnight in 10% neutral-buffered formalin. For inflation fixation, lungs were intratracheally instilled with 10% formalin, followed by immersion in fresh fixative. After fixation, tissues were processed, paraffin-embedded, and sectioned at 4 μm. Both inflated and non-inflated lung sections were stained with hematoxylin and eosin (H&E) and digitally scanned using a Pannoramic Confocal digital scanner (3DHISTECH). Lung histopathological injury was scored according to the American Thoracic Society workshop report on experimental acute lung injury in animals [39]. The score incorporated alveolar neutrophil accumulation (*A*), interstitial neutrophil accumulation (*B*), hyaline membranes (*C*), intra-alveolar proteinaceous material (*D*), and alveolar septal thickening (*E*) and was calculated as [(20 × *A*) + (14 × *B*) + (7 × *C*) + (7 × *D*) + (2 × *E*)]/(number of fields × 100). Scores ranged from 0 to 1, with higher values indicating more severe injury.

### Quantification of total protein and cell count in mouse BALF

BALF was collected and processed as previously described [40]. Total protein concentration was measured using a bicinchoninic acid (BCA) protein assay kit (7780S, Cell Signaling Technology). The total cell count was determined using a hemocytometer.

### Lung wet/dry ratio

To evaluate lung edema, the wet/dry ratio was determined. Lungs were first weighed to obtain the wet weight and then dried at 80 °C for 48 h. After drying, the dry weight was recorded, and the wet/dry ratio was calculated.

### Flow cytometry staining

Single-cell suspensions were prepared from human BALF, nontumor lung tissues collected from mice in the indicated experimental groups, and mouse subcutaneous tumor tissues as previously described [41]. Cells were stained with a fixable viability dye (Zombie UV, #423108, BioLegend) to exclude dead cells and incubated with an Fc receptor blocking solution. Surface antigens were then stained by incubation with freshly prepared antibody cocktails for 30 min at 4 °C. For intracellular antigen detection, surface-stained cells underwent fixation and permeabilization, followed by incubation with optimal concentrations of fluorophore-conjugated antibody cocktails for 20 min at room temperature in the dark. Cells were washed, acquired on a BD LSRFortessa flow cytometer, and analyzed using FlowJo software (https://www.flowjo.com). Antibodies were listed in Table S4. The gating strategy for murine lung immune cell clusters was adapted from established lung myeloid cytometry literature [41,42]. CD45^+^ viable singlets were first identified. Lymphoid populations were gated as NK cells (CD45^+^NK1.1^+^), CD3^+^ T cells, CD4^+^ T cells, CD8^+^ T cells, and Treg (CD4^+^CD25^+^Foxp3^+^). For lung myeloid analysis, CD11c^+^ cells were used to identify AMs, whereas CD11c^−^CD11b^+^ cells were used to resolve IMs, myeloid-derived suppressor cells (MDSCs), lymphocyte antigen 6 complex, locus C (Ly6C)^−^ and Ly6C^+^ monocytes, and CCR2^+^ MoMΦ. CD11b^−^ major histocompatibility complex class II (MHC II)^+^ cells were intentionally excluded because they predominantly displayed a lymphoid phenotype [partial CD19 expression, lack of Ly6C/lymphocyte antigen 6 complex, locus G (Ly6G)/CD64/CCR2]. The resulting gating ensured accurate representation of bona fide myeloid clusters.

### Multiplexed immunofluorescence and analysis

Multiplexed immunofluorescence was performed on formalin-fixed, paraffin-embedded (FFPE) tissue sections using the Opal system (PerkinElmer), following the manufacturer’s protocol. Briefly, deparaffinized sections underwent fixation in 4% paraformaldehyde and heat-induced epitope retrieval (HIER) in citrate buffer (pH 6). Staining proceeded sequentially through multiple rounds; each round comprised blocking endogenous peroxidase activity and nonspecific protein binding, incubation with a primary antibody, and incubation with a corresponding horseradish peroxidase (HRP)-conjugated polymer secondary antibody (PerkinElmer). Tyramide signal amplification (TSA) employed distinct fluorophores covalently bound via HRP activity. An additional HIER step in a heated citrate buffer for 10 min followed each TSA reaction to strip antibodies before initiating the subsequent staining round. Upon completion of all staining cycles, sections were counterstained with 4′,6-diamidino-2-phenylindole (DAPI). Primary antibodies and dilutions used were as follows: CCR2 (1:300, MA5-41175, Thermo Scientific), CXCL9 (1:50, #701117, Thermo Scientific), CXCL10 (1:100, MAB4661, R&D Systems), and CD8α (1:500, ab217344, Abcam). Whole-slide scanning and multispectral image acquisition utilized the Vectra platform with Vectra (version 3.3) and Phenochart software (version 1.0.12, Akoya Biosciences). Spectral unmixing was performed using inform software (version 2.4.8, Akoya Biosciences) with a spectral library generated from the individual fluorophores. Quantitative single-cell image analysis was conducted in QuPath (version 0.4.3) [43], and spatial statistics were computed in R using the spatstat package. In multiplex immunofluorescence images from DT + anti-PD-1-treated lungs, CCR2^+^ cells and CD8^+^ T cells were segmented and phenotyped in QuPath, and μm-calibrated *x*–*y* coordinates of cell centroids were exported. In R, point-pattern objects were constructed and, for each CD8^+^ T cell, the Euclidean distance to its nearest CCR2^+^ cell was calculated. Distances were grouped into 4 bins (0 to 20 μm, 20 to 40 μm, 40 to 60 μm, and >60 μm). For each image, the number of CD8^+^ T cells in each bin was expressed as a proportion of total CD8^+^ T cells, providing a distance-stratified measure of short-range enrichment around CCR2^+^ cells.

### Harvest, stimulation, and transient transfection of bone marrow-derived macrophages

Bone marrow cells isolated from mouse femurs and tibiae underwent 6-d differentiation into bone marrow-derived macrophage (BMDMs) in DMEM containing 10% FBS, 1% penicillin–streptomycin, and 5% macrophage colony-stimulating factor (M-CSF). At day 7, cultures were switched to M-CSF-free DMEM for 48 h. For interferon-γ (IFN-γ) stimulation experiments, 8 × 10^5^ BMDMs per well were plated in 6-well plates overnight for adherence. Cells received stimulation with 50 ng/ml IFN-γ (#575306, BioLegend) alone or co-administered with 25 μM ruxolitinib (INCB18424, MedChemExpress). Under IFN-γ stimulation, BMDMs were transfected with 125 pM signal transducer and activator of transcription 1 (*Stat1*) small interfering RNA (siRNA) (AM16708, Thermo Scientific) or Ctrl siRNA (AM4611, Thermo Scientific) using Lipofectamine 3000 transfection reagent (L3000015, Thermo Scientific) for 48 h to achieve *Stat1* knockdown.

### Protein extraction and Western blotting

Analysis of protein expression in the BALF cells, mouse lungs, and BMDMs was performed as described previously [44]. Total protein was extracted from patient BALF and from mouse lung tissue and BMDMs by homogenizing the collected cells or tissues in ice-cold radioimmunoprecipitation assay lysis buffer containing 1 mM phenylmethylsulfonyl fluoride and protease and phosphatase inhibitor cocktails. The lysates were then incubated on ice for 30 min and centrifuged at 12,000*g* for 15 min at 4 °C, and the supernatants were recovered. Protein concentration was quantified using a BCA protein assay kit. Proteins were resolved by electrophoresis on 10% or 15% sodium dodecyl sulfate–polyacrylamide gel electrophoresis (SDS-PAGE) and subsequently transferred to polyvinylidene fluoride membranes. After blocking, the membranes were incubated with primary antibodies [signal transducer and activator of transcription (STAT1), 1:1,000, #9172, Cell Signaling Technology; p-STAT1, 1:1,000, #9167, Cell Signaling Technology; CXCL9, 1:500; CXCL10, 1:500, #42912, Cell Signaling Technology], followed by incubation with HRP-conjugated anti-rabbit IgG secondary antibodies. Protein bands were visualized using enhanced chemiluminescence and detected with a Tanon 5200 digital imager. Band intensities were quantified in ImageJ (https://imagej.nih.gov/ij/) and normalized to β-actin (1:2,000, #4970, Cell Signaling Technology).

### Real-time quantitative polymerase chain reaction

Analysis of mRNA expression in the BALF cells and BMDMs was performed as described previously [45]. Total RNA was isolated from samples using TRIzol reagent (Invitrogen), and concentrations were determined spectrophotometrically (NanoDrop). We synthesized cDNA with the First Strand cDNA Synthesis Kit (K1612, Thermo Scientific). Quantitative polymerase chain reaction (qPCR) was performed in 50-μl reactions containing SYBR Green Universal Master Mix (#4309155, Applied Biosystems) and sequence-specific primers, using an ABI Prism 7500 sequence detection system (Applied Biosystems). The mRNA expression levels were normalized to glyceraldehyde-3-phosphate dehydrogenase (GAPDH) (human) or 18S ribosomal RNA (mouse). Primer sequences are listed in Table S5.

### Enzyme-linked immunosorbent assay

IFN-γ concentrations in BALF supernatants were measured using a commercial human IFN-γ ELISA Kit (KHC4021, Thermo Scientific), following the manufacturer’s instructions. Concentrations were determined from a standard curve generated with kit-provided purified IFN-γ.

### In vitro Transwell system for CXCR3, CXCL9, and CXCL10 blockade

For T cell stimulation, 24-well plates were coated with 1 μg/ml anti-CD3 and 3 μg/ml anti-CD28 antibodies (Thermo Fisher Scientific) in a humidified incubator (37 °C, 5% CO_2_) for 1 to 2 h. Lungs harvested from *Foxp3-DTR-GFP* mice administered DT plus anti-PD-1 were digested with collagenase IV (1 mg/ml) plus DNase I (50 U/ml) at 37 °C for 20 min to generate single-cell suspensions. Lung-derived peripheral blood mononuclear cells (PBMCs) were subsequently isolated using density gradient centrifugation (800*g* for 20 min at room temperature, without brake). Isolated lung PBMCs were cultured in RPMI 1640 medium supplemented with 10% FBS, 10 mM 4-(2-hydroxyethyl)-1-piperazineethanesulfonic acid (Hepes), 70 μM β-mercaptoethanol, 1% penicillin/streptomycin, and 20 ng/ml recombinant interleukin-2 (IL-2). Cells were maintained on the precoated plates under standard culture conditions (37 °C, 5% CO_2_). Subculturing was performed every 2 to 3 d to maintain optimal density, with an activation and expansion period of 6 to 7 d.

#### CXCR3 blockade

The stimulated lung-derived T cells described above were incubated with anti-CXCR3 antibody (10 μg/ml, Bio X Cell) or isotype IgG control (Bio X Cell) for 30 min at 37 °C, and then 5 × 10^4^ cells were added to the upper chamber of each well in a 96-well Transwell system. Peritoneal macrophages from DT + anti-PD-1-treated mice were added to the lower chamber at 2 × 10^4^ cells per chamber. After 2-h migration at 37 °C/5% CO_2_, cells in the lower chamber were harvested for flow cytometry.

#### Chemokine blockade

Using the same Transwell system, peritoneal macrophages in the lower chamber were incubated for 45 min at 37 °C with anti-CXCL9 (3 μg/ml, AF492-SP, R&D Systems) and/or anti-CXCL10 (3 μg/ml, AF466-SP, R&D Systems) before addition of the stimulated lung-derived T cells to the upper chamber. Migrated cells in the lower chamber were quantified by flow cytometry after 2 h (37 °C/5% CO_2_).

#### Recombinant chemokine supplementation

Using the same Transwell system, recombinant murine CXCL9 (0.15 μg/ml, #250-18-20UG, Thermo Scientific) and/or CXCL10 (0.1 μg/ml, #250-16-25UG, Thermo Scientific) was added to the lower chamber before addition of the stimulated lung-derived T cells to the upper chamber. Migration proceeded for 2 h (37 °C/5% CO_2_), after which lower chamber cells were collected for flow cytometry.

### Pharmacological intervention studies

C57BL/6 *Foxp3-DTR-GFP* mice received daily intraperitoneal injections (100 μl per mouse) of cenicriviroc (CVC; dual CCR2/CCR5 antagonist, 20 mg/kg, Cayman Chemical), RS102895 (selective CCR2 antagonist, 20 mg/kg, Cayman Chemical), maraviroc (selective CCR5 antagonist, 20 mg/kg, Cayman Chemical), or AMG487 (CXCR3 antagonist, 5 mg/kg, Cayman Chemical). Treatment commenced 3 d prior to the first DT dose and continued daily for 28 d.

### Statistical analyses

Data are presented as mean ± standard error of the mean (SEM). Statistical analyses were performed using GraphPad Prism software. For comparisons between 2 groups, significance was determined using 2-tailed Mann–Whitney tests. For comparisons among 3 or more groups, significance was determined using ordinary one-way analysis of variance (ANOVA) followed by Tukey’s multiple comparisons test. Tumor growth curves were analyzed using 2-way ANOVA followed by Dunnett’s multiple comparisons test. Survival curves (Kaplan–Meier) were analyzed by the log-rank (Mantel-Cox) test. A *P* value of <0.05 was considered statistically significant.

## Results

### Analysis of BALF from CIP^+^ patients revealed substantial accumulation of CD8^+^ T cells and CCR2^+^ MoMΦ

**Fig. 1.**
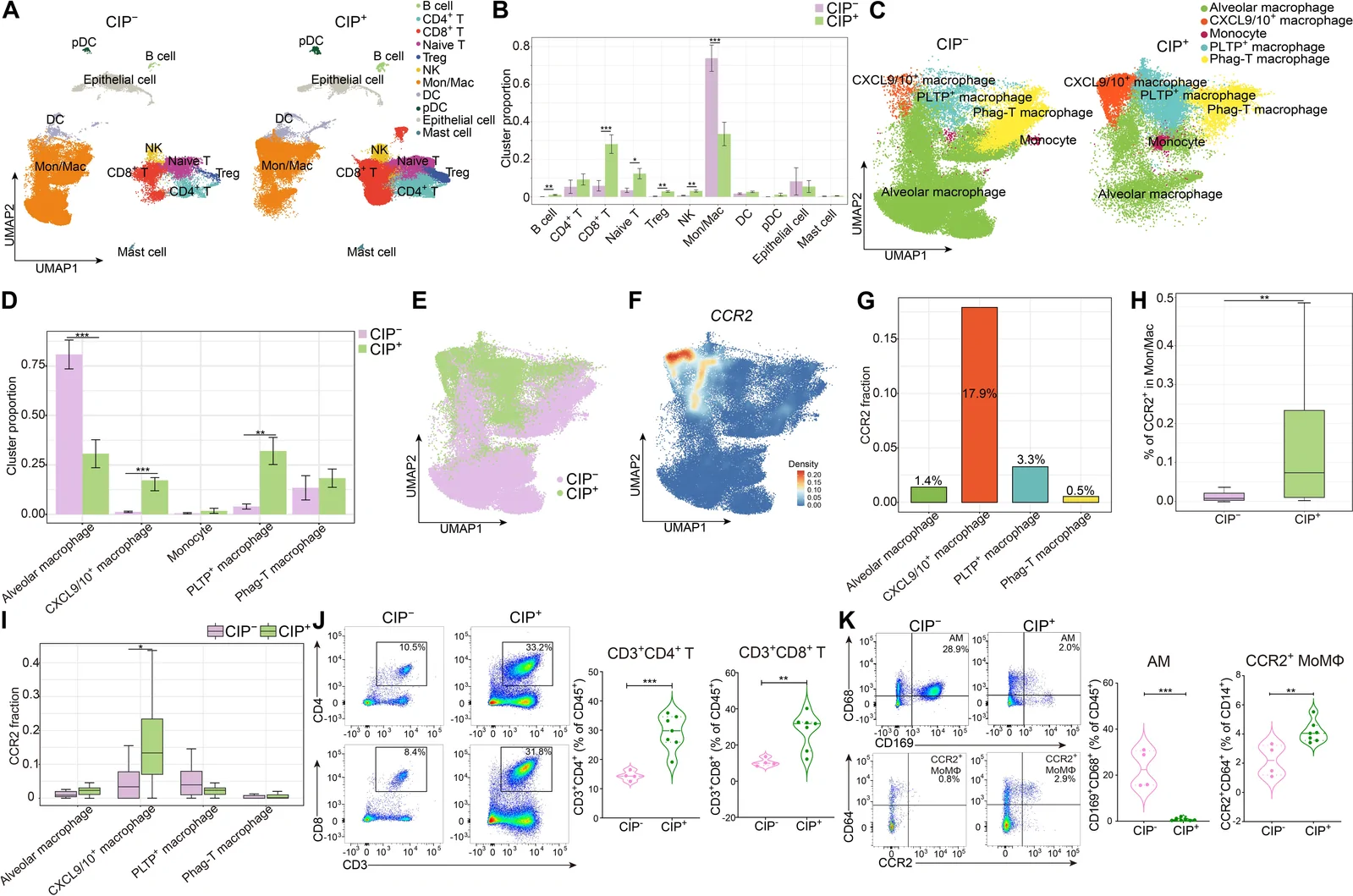
CD8^+^ T cells and CCR2^+^ MoMΦ are expanded in BALF from CIP^+^ patients. (A) UMAP plot of 174,886 high-quality BALF cells from 2 single-cell datasets [20,21], colored by 11 annotated cell types. Clinical characteristics of the patients were detailed in Table S1. (B) Proportions of the 11 annotated cell types in BALF of CIP^−^ and CIP^+^ patients. (C) The UMAP plot of 85,951 monocytes/macrophages from BALF of CIP^−^ and CIP^+^ patients revealed 5 cell types. (D) Proportion of each monocyte/macrophage cluster in BALF of CIP^−^ and CIP^+^ patients. (E) UMAP plot of monocytes/macrophages, colored by CIP^−^ and CIP^+^ patients. (F) Density plot of *CCR2* expression in monocytes/macrophages from the integrated BALF scRNA-seq data of CIP^−^ and CIP^+^ patients. (G) Proportion of CCR2^+^ cells within each monocyte/macrophage cluster from the integrated BALF scRNA-seq data of CIP^−^ and CIP^+^ patients. (H) Comparison of the proportion of CCR2^+^ cells among monocytes/macrophages between CIP^−^ and CIP^+^ patients. (I) Comparison of the proportion of CCR2^+^ cells in each monocyte/macrophage cluster between CIP^−^ and CIP^+^ patients. (J) Representative flow cytometry plots and quantification of CD3^+^CD4^+^ and CD3^+^CD8^+^ T cells in BALF from the independent validation cohort of 4 CIP^−^ and 7 CIP^+^ patients. (K) Representative flow cytometry plots and quantification of CD68^+^CD169^+^ cells (AMs) and CD14^+^CD64^+^CCR2^+^ cells (CCR2^+^ MoMΦ) in BALF from the independent validation cohort of 4 CIP^−^ and 7 CIP^+^ patients. Data collected from 2 independent experiments. Clinical characteristics of the patients were detailed in Tables S2 and S3. Two-tailed Mann–Whitney *U* test was used; significance is shown as *P < 0.05, **P < 0.01, ***P < 0.001. BALF, bronchoalveolar lavage fluid; CCR2, C-C motif chemokine receptor 2; CIP, immune checkpoint inhibitor-associated pneumonitis; CXCL9, C-X-C motif chemokine ligand 9; CXCL10, C-X-C motif chemokine ligand 10; DC, dendritic cell; MoMΦ, monocyte-derived macrophage; Mon/Mac, monocytes/macrophages; NK, natural killer; pDC, plasmacytoid dendritic cell; Phag-T macrophage, T cell-phagocytosing macrophage; PLTP, phospholipid transfer protein; Treg, regulatory T cell; UMAP, uniform manifold approximation and projection; AM, alveolar macrophage.

To perform an unbiased analysis of immune cell populations in CIP, we integrated high-quality scRNA-seq profiles of 174,886 BALF cells from 18 CIP^+^ patients and 12 CIP^−^ patients (Table S1). This cohort comprised (a) NSCLC patients treated with PD-1/PD-L1 blockade who developed CIP (CIP^+^, *n* = 7) or did not develop CIP (CIP^−^, *n* = 6) [20] and (b) patients who developed CIP during PD-1/PD-L1 blockade therapy (10 NSCLC and 1 melanoma patient; CIP^+^, *n* = 11) and NSCLC patients who were not receiving ICIs and had not developed CIP (CIP^−^, *n* = 6) [21]. Based on the expression of canonical gene markers, we identified and annotated 11 major cell type clusters, encompassing all expected immune populations: B cells, CD4^+^ T cells, CD8^+^ T cells, naïve T cells, Tregs, NK cells, monocytes/macrophages (Mon/Mac), dendritic cells (DCs), plasmacytoid dendritic cells (pDCs), epithelial cells, and mast cells (Fig. 1A and Fig. S1A). Among these, monocytes/macrophages were the most abundant in the integrated dataset, accounting for 49.1% of sequenced cells, followed by T cells (35.6%). Notably, the most significant differences between CIP^+^ and CIP^−^ patients were observed within the monocyte/macrophage and CD8^+^ T cell compartments. Specifically, we detected significant alterations in the cellular composition: CIP^+^ patients exhibited a marked reduction in the fraction of monocytes/macrophages and a significant increase in CD8^+^ T cells compared to CIP^−^ patients (Fig. 1A and B). Subclustering of the monocytes/macrophages revealed 5 well-defined clusters annotated based on their transcriptomic profiles and published markers [14] (Fig. 1C and Fig. S1B). We found that the proportion of AMs [marked by macrophage receptor with collagenous structure (*MARCO*), peroxisome proliferator activated receptor gamma (*PPARG*), transglutaminase 2 (*TGM2*), procollagen C-endopeptidase enhancer 2 (*PCOLCE2*), inhibin subunit β A (*INHBA*)] was significantly decreased in the BALF of CIP^+^ patients compared to CIP^−^ patients (Fig. 1C and D and Fig. S1B and C), explaining the overall reduction in the monocyte/macrophage compartment. Conversely, the proportions of CXCL9/10^+^ macrophages [marked by *CXCL10*, *CXCL9*, ficolin 1 (*FCN1*), calcium homeostasis modulator family member 6 (*CALHM6*), *CCL2*)] and PLTP^+^ macrophages [marked by phospholipid transfer protein (*PLTP*), interleukin-1β (*IL1B*), legumain (*LGMN*), secreted phosphoprotein 1 (*SPP1*), matrix metallopeptidase 9 (*MMP9*)] were significantly increased in CIP^+^ patients (Fig. 1C and D and Fig. S1B to E). Notably, *CCR2* expression was largely confined to the CXCL9/10^+^ macrophage cluster, with CCR2^+^ cells accounting for approximately 17.9% of this population (Fig. 1E to G). The proportion of CCR2^+^ macrophages within the CXCL9/10^+^ cluster in BALF was significantly higher in CIP^+^ patients than in CIP^−^ patients (Fig. 1H and I and Fig. S1F). Consistent with this, *CCL2* was also preferentially expressed by CXCL9/10^+^ macrophages and showed increased expression within this cluster in CIP^+^ patients compared with CIP^−^ patients (Fig. S1G to I).

To investigate the relationships between monocyte/macrophage clusters, we computed cellular differentiation trajectories using Palantir, designating monocytes as the starting point. Pseudotime analysis indicated that CXCL9/10^+^ macrophages represented a differentiated cellular state (Fig. S2A). Determination of differentiation potential (entropy) values revealed that CXCL9/10^+^ macrophages exhibited the lowest level of cellular plasticity (Fig. S2B), suggesting that these macrophages in CIP were highly specialized and functionally active. The differentiation trajectory also indicated that CXCL9/10^+^ macrophages were the endpoint of monocyte differentiation (Fig. S2C).

To validate these scRNA-seq findings at the protein level, we performed flow cytometric immunophenotyping of BALF from an independent clinical cohort of NSCLC patients who had received anti-PD-1 therapy, including 7 patients who developed CIP (CIP^+^) and 4 demographically matched patients who did not develop CIP (CIP^−^) (Fig. S2D and Tables S2 and S3).

Flow cytometry showed that the proportions of CD3^+^CD4^+^ and CD3^+^CD8^+^ T cells were significantly increased in CIP^+^ patients compared with CIP^−^ patient controls (Fig. 1J), indicating increased T cell accumulation in the BALF of patients with CIP. Analysis of monocytes/macrophages within the same cohort confirmed the scRNA-seq data: CIP^+^ patients showed a significantly reduced proportion of CD68^+^ cells (Fig. S2E), specifically AMs (CD169^+^CD68^+^) (Fig. 1K). Importantly, the proportion of inflammatory CCR2^+^ MoMΦ (CD14^+^CCR2^+^CD64^+^) was significantly increased in CIP^+^ patients relative to CIP^−^ patients (Fig. 1K).

These results demonstrate that CIP is associated with significant alterations in pulmonary immune cells, characterized by increased T cells and inflammatory CCR2^+^ MoMΦ.

### Treg depletion plus PD-1 blockade was associated with CIP-like lung injury and accumulation of CD8^+^ T cells and CCR2^+^ MoMΦ in mouse lung tissues

**Fig. 2.**
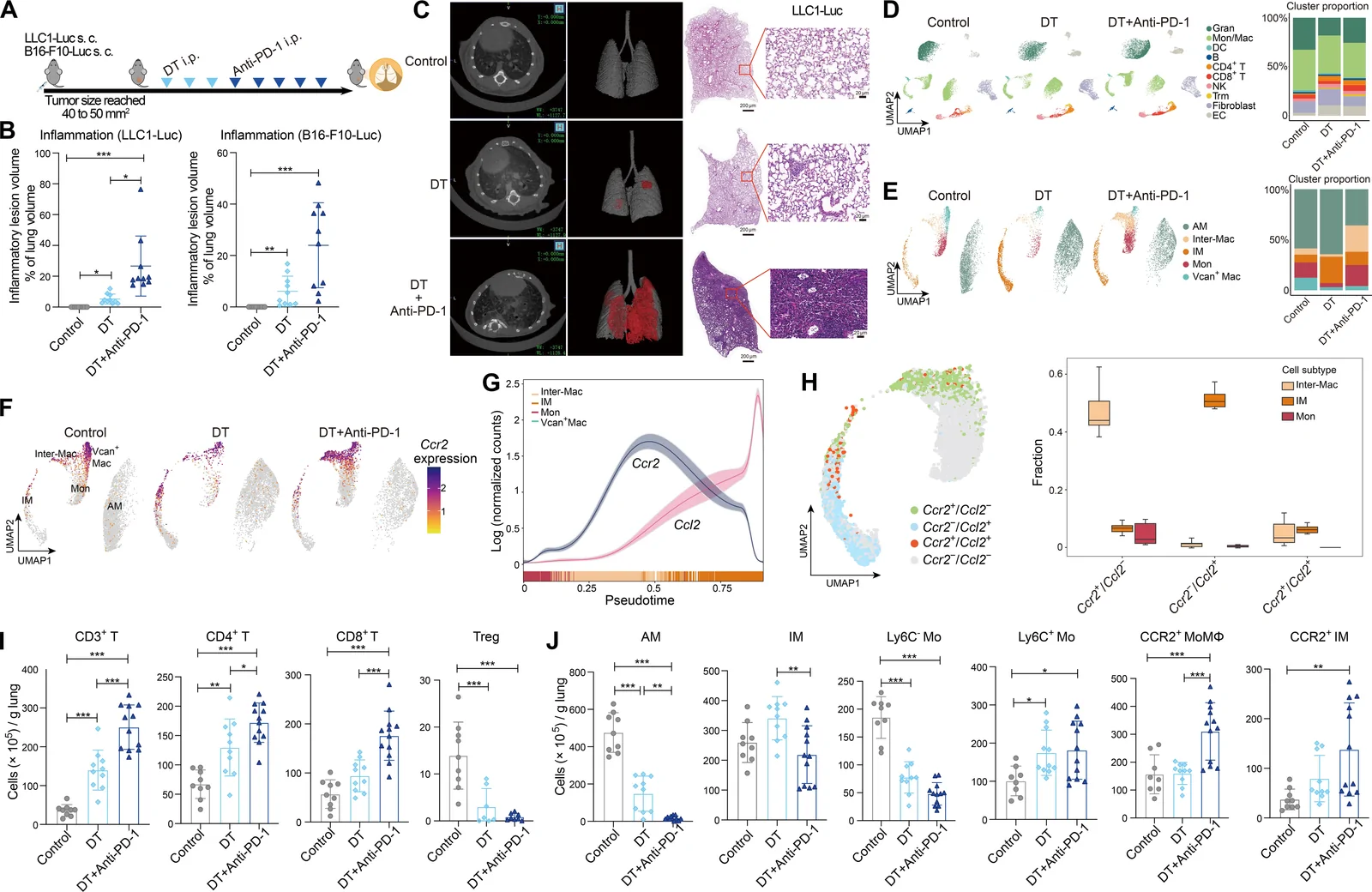
DT + anti-PD-1 treatment recapitulates CIP clinical features and induces expansion of CD8^+^ T cells and CCR2^+^ MoMΦ in lung tissue of *Foxp3-DTR-GFP* mice. (A) *Foxp3-DTR-GFP* mice were subcutaneously inoculated with LLC1-Luc or B16-F10-Luc tumor cells. When the mean tumor size reached 40 to 50 mm^2^, tumor-bearing mice were intraperitoneally administered PBS or DT (3 doses at 2-d intervals). Two days after the final DT treatment, mice received intraperitoneal injections of either anti-PD-1 or control IgG (5 doses at 2-d intervals). Treatment groups were labeled: control, PBS + IgG; DT, DT + IgG; DT + anti-PD-1, DT + anti-PD-1. (B) Quantification of inflammatory foci in lung tissues from tumor-bearing mice across treatment groups (*n* = 10 per group). Inflammation was quantified using CT-based 3-dimensional (3D) reconstruction by calculating inflammatory lesion volume and normalizing it to total lung volume. (C) Representative micro-CT images (left), 3D-reconstructed lung tissue (gray) and inflammatory foci (red) (middle), and H&E-stained sections (right) from LLC1-Luc-bearing mice across treatment groups (*n* = 10 per group). (D) UMAP plot of 39,355 cells from 12 mouse lungs (control, *n* = 4; DT, *n* = 4; DT + anti-PD-1, *n* = 4), colored by 10 annotated cell types, with a stacked bar plot showing the proportion of each cell type in each group. (E) UMAP plot of 15,127 monocytes/macrophages from mouse lungs stratified by experimental group (control, DT, DT + anti-PD-1), revealing 5 transcriptionally distinct clusters, with a stacked bar plot showing the proportion of each monocyte/macrophage cluster in each group. (F) UMAP plot of *Ccr2* expression within monocytes/macrophages across treatment groups in mice. (G) Expression trends of *Ccr2* and *Ccl2* along the inferred monocyte-to-macrophage pseudotime trajectory. (H) UMAP plot of monocytes/macrophages colored by *Ccr2* and *Ccl2* expression status (*Ccr2*^+^/*Ccl2*^−^, *Ccr2*^−^/*Ccl2*^+^, *Ccr2*^+^/*Ccl2*^+^, *Ccr2*^−^/*Ccl2*^−^). Box plot showing the fraction of cells in each *Ccr2*/*Ccl2* category within each monocyte/macrophage cluster (Mon, Inter-Mac, IM). (I) Flow cytometric quantification of pulmonary lymphocyte populations, including CD3^+^ T cells, CD4^+^ T cells, CD8^+^ T cells, and Tregs. (J) Flow cytometric quantification of pulmonary myeloid populations, including AMs (CD11c^+^CD64^+^CD24^−^), IMs (CD11b^+^CD64^+^MHC II^+^), Ly6C^−^ Mo (CD11b^+^Ly6C^−^Ly6G^−^), Ly6C^+^ Mo (CD11b^+^Ly6C^+^Ly6G^−^), CCR2^+^ MoMΦ (CD11b^+^Ly6C^+^CCR2^+^CD64^+^), and CCR2^+^ IMs (CD11b^+^CD64^+^MHC II^+^CCR2^+^). The gating strategy was detailed in Fig. S5A. Absolute counts were presented as cells per gram of lung tissue. For each mouse, the total number of live CD45^+^ cells recovered from the digested lung was multiplied by the frequency of the indicated cell population within live CD45^+^ cells and divided by the recorded lung wet weight. Data collected from 4 independent experiments. Control (*n* = 9), DT (*n* = 7 to 11), DT + anti-PD-1 (*n* = 8 to 12). One-way ANOVA with Tukey’s test was used; significance is shown as *P < 0.05, **P < 0.01, ***P < 0.001. AM, alveolar macrophage; B, B cell; B16-F10-Luc, luciferase-expressing B16-F10 melanoma cells; CT, computed tomography; DT, diphtheria toxin; DTR, diphtheria toxin receptor; EC, endothelial cell; Gran, granulocyte; H&E, hematoxylin and eosin; IM, interstitial macrophage; Inter-Mac, intermediate-stage inflammatory macrophage; LLC1-Luc, luciferase-expressing Lewis lung carcinoma 1 cells; Ly6C, lymphocyte antigen 6 complex, locus C; Ly6C^−^ Mo, Ly6C-negative monocyte; Ly6C^+^ Mo, Ly6C-positive monocyte; Ly6G, lymphocyte antigen 6 complex, locus G; MHC II, major histocompatibility complex class II; Mon, monocyte; PD-1, programmed cell death protein 1; Trm, tissue-resident memory T cell; Vcan^+^ Mac, versican-expressing macrophage.

To investigate the immunological mechanisms underlying CIP, we employed the *Foxp3-DTR-GFP* mouse model. However, previous studies using this model provided little lung-specific imaging, histopathologic, or detailed immunophenotypic characterization of pulmonary immune cell clusters. In the present study, we assessed the effects of Treg depletion combined with anti-PD-1 antibody treatment on tumor growth and pneumonitis development in *Foxp3-DTR-GFP* mice bearing LLC1-Luc and B16-F10-Luc tumors (Fig. 2A). Overall, compared to DT alone or control, DT + anti-PD-1 treatment inhibited tumor growth in both the LLC1-Luc and B16-F10-Luc models, although the magnitude of this effect was tumor type-dependent (Fig. S3A to F). This response was likely driven by a significant increase in the proportion of CD4^+^ and CD8^+^ T cells, as well as a marked enrichment of IFN-γ^+^CD4^+^ and IFN-γ^+^CD8^+^ T cells within the tumor microenvironment in the DT + anti-PD-1-treated mice, compared to the DT or control groups (Fig. S3G and H). Furthermore, we observed that LLC1-Luc-bearing mice treated with DT + anti-PD-1 had a significantly shorter survival time compared to the DT group (Fig. S3C), potentially due to the onset of severe irAEs. Second, we performed a comprehensive analysis of mouse lung tissues. Overall, the characteristic features of pneumonitis were induced by Treg depletion combined with anti-PD-1 antibody therapy, irrespective of tumor type. Micro-CT revealed characteristic changes in the DT + anti-PD-1 group, including ground-glass opacities, centrilobular nodules, and fibrotic bands. Histological examination confirmed substantial lymphocytic and mononuclear infiltration within the lung parenchyma and alveolar spaces, along with thickened alveolar septa in the DT + anti-PD-1 group (Fig. 2B and C and Fig. S3I to K). These features mirror the radiologic and pathologic alterations observed in patients with ICI-induced pneumonitis [30]. Three-dimensional reconstruction of lung tissue based on CT scans and quantification of inflamed areas demonstrated a significant increase in pneumonitis area in the DT + anti-PD-1 group compared to control groups (DT and control) (Fig. 2B and C and Fig. S3I). Consistent with these findings, histologic injury scores were significantly increased in both the DT and DT + anti-PD-1 groups compared with the control group, and were further elevated in the DT + anti-PD-1 group relative to the DT group (Fig. S3J and K). Additionally, BALF protein concentration, total BALF cell counts, and lung wet/dry weight ratios showed significant increases in DT + anti-PD-1-treated mice relative to control or DT mice (Fig. S3L to N). The DT group exhibited an intermediate phenotype. Significantly increased lymphocytic and monocytic immune infiltration was also observed in other indicated organs of DT + anti-PD-1-treated mice (Fig. S3O).

Next, we performed a comprehensive scRNA-seq analysis on lung tissues from LLC1-Luc-bearing mice. Unsupervised clustering identified a total of 39,355 high-quality cells, defining 10 distinct clusters based on canonical gene marker expression (Fig. 2D and Fig. S4A). Consistent with the single-cell transcriptomic profiles observed in the BALF from CIP^+^ patients, anti-PD-1 treatment reduced the proportion of monocytes/macrophages [Mon/Mac; *Cd68*, *cytochrome b-245 β chain* (*Cybb*)] in the mouse lungs (control: 41.9%; DT: 38.7%; DT + anti-PD-1: 35.6%), whereas the proportions of CD4^+^ T cells (control: 1.5%; DT: 5.2%; DT + anti-PD-1: 5.2%) and CD8^+^ T cells (control: 3.5%; DT: 2.6%; DT + anti-PD-1: 5.7%) were increased (Fig. 2D). Further subclustering of the monocyte/macrophage compartment based on transcriptomic profiles revealed 5 clusters: AMs [placenta expressed transcript 1 (*Plet1*), *Pparg*, *Marco*], intermediate-stage inflammatory macrophages (Inter-Mac; *Ly6c2*, *Ccr2*), IMs [*Ccl2*, folate receptor β (*Folr2*), lymphatic vessel endothelial hyaluronan receptor 1 (*Lyve1*)], monocytes [Mon; pou class 2 homeobox 2 (*Pou2f2*), *Cd300e*], and Vcan*^+^* macrophages [Vcan*^+^* Mac; lymphocyte antigen 6 complex, locus A2 (*Ly6a2*), versican (*Vcan)*] (Fig. 2E and Fig. S4B). Among these, the abundance of AMs was reduced in the lungs of DT + anti-PD-1-treated mice compared to DT or control mice (control: 58.6%; DT: 64.4%; DT + anti-PD-1: 35.8%). Conversely, the abundance of Inter-Mac was dramatically increased in DT + anti-PD-1-treated mice (control: 6.2%; DT: 2.5%; DT + anti-PD-1: 26.0%), representing an ~13-fold increase compared to DT and an ~4.3-fold increase compared to control (Fig. 2E). We observed *Ccr2* expression predominantly in Inter-Mac, Vcan^+^ Mac, and partially in IMs. Notably, *Ccr2*^+^ Inter-Mac were increased in DT + anti-PD-1-treated mice compared to control groups (DT and control) (Fig. 2F). *Ccl2* expression was primarily localized to IMs and was also elevated in DT + anti-PD-1-treated mice (Fig. S4C to E).

Using Palantir, we computed differentiation trajectories for the monocyte/macrophage compartment, designating monocytes as the starting point. Pseudotime analysis indicated that IMs and AMs represent differentiated cellular states. Differentiation potential (entropy) assessment revealed that IMs and AMs exhibited the lowest levels of cellular plasticity (Fig. S4F and G), highlighting their highly specialized state. In contrast, Inter-Mac displayed intermediate pseudotime and entropy values (Fig. S4F and G), suggesting a transitional state during monocyte differentiation. Trajectory analysis revealed 3 distinct terminal differentiation states for monocytes: IMs, Vcan^+^ Mac, and AMs (Fig. S4H). The trajectory paths indicated that monocytes first differentiate into Inter-Mac prior to their final maturation into IMs (Fig. S4H). Furthermore, we performed a pseudotime gene expression trend plot analysis using Palantir, examining the dynamic expression of *Ccr2* and *Ccl2* across the inferred trajectory (Fig. 2G). At early pseudotime, *Ccr2* expression in monocytes progressively increased and reached its highest levels within Inter-Mac. With further progression toward IMs, *Ccr2* expression declined, whereas *Ccl2* expression increased and became dominant at the endpoint of the trajectory. To strengthen this interpretation, we quantified the proportions of *Ccr2*^+^ single-positive, *Ccl2*^+^ single-positive, and *Ccr2*^+^*Ccl2*^+^ double-positive cells within each cluster (monocytes, Inter-Mac, IMs) (Fig. 2H). In early/intermediate pseudotime (Inter-Mac stage), *Ccr2*^+^ single-positive cells predominated (44.3% *Ccr2*^+^ only, 1.2% *Ccl2*^+^ only, 3.3% double-positive). In contrast, in the late pseudotime (IM stage), *Ccl2*^+^ single-positive cells became the major population (50.5%), with a marked reduction in *Ccr2* expression. Double-positive *Ccr2*^+^*Ccl2*^+^ cells were most abundant at intermediate pseudotime positions between Inter-Mac and IMs, consistent with a transitional state. Together, these findings suggest that Inter-Mac and IMs represent sequential stages along a monocyte-to-macrophage differentiation continuum rather than independent macrophage clusters.

We employed a flow cytometry panel to validate lung immune cell populations (Fig. S5A). Consistent with scRNA-seq data of mouse lung tissue, the absolute counts of CD3^+^ T cells, CD4^+^ T cells, and CD8^+^ T cells were significantly increased, while that of Treg cells was significantly decreased in the lungs of LLC1-Luc-bearing mice receiving DT + anti-PD-1 treatment compared to DT or control mice (Fig. 2I and Fig. S5B to D). The absolute counts of NK cells did not show any significant differences among the 3 groups (Fig. S5E). Notably, the absolute counts of CD8^+^ T cells were significantly elevated only in the lungs of mice treated with DT + anti-PD-1 (Fig. 2I). Subsequent analysis focused on monocytes/macrophages: AMs (CD11c^+^CD11b^−^CD64^+^CD24^−^), IMs (CD11c^−^CD11b^+^CD64^+^MHC II^+^), Ly6C^−^ monocytes (Ly6C^−^ Mo; CD11c^−^CD11b^+^Ly6C^−^Ly6G^−^), Ly6C^+^ monocytes (Ly6C^+^ Mo; CD11c^−^CD11b^+^Ly6C^+^Ly6G^−^), CCR2^+^ MoMΦ (Ly6C^+^CCR2^+^CD64^+^; gated from Ly6C^+^ Mo), and CCR2^+^ IMs (CCR2^+^CD64^+^MHC II^+^; gated from IMs). Compared with control mice, the absolute counts of AMs were significantly reduced in the lungs of both DT- and DT + anti-PD-1-treated mice. Notably, anti-PD-1 treatment following DT (DT + anti-PD-1) further decreased AM counts compared with DT alone. The absolute counts of IMs were significantly reduced in the lungs of DT + anti-PD-1-treated mice compared with DT-treated mice (Fig. 2J and Fig. S5F and G). The absolute counts of Ly6C^−^ Mo were significantly lower in the lungs of DT- and DT + anti-PD-1-treated mice than in control mice, whereas the absolute counts of pro-inflammatory Ly6C^+^ Mo were significantly higher in DT- and DT + anti-PD-1-treated mice than in control mice (Fig. 2J and Fig. S5H). Importantly, a marked increase in the absolute counts of inflammatory CCR2^+^ MoMΦ and CCR2^+^ IMs was observed in the lungs of DT + anti-PD-1-treated mice (Fig. 2J and Fig. S5I and J). By contrast, the absolute counts of CD11b^+^ cells and MDSCs in lung tissue did not differ significantly among the 3 groups (Fig. S5K to M).

To further confirm the monocytic origin of CCR2^+^ macrophages, we performed lineage tracing using *Ccr2^ertcre^Rosa^tdTomato^* mice. Blocking the CCR2–CCL2 interaction with anti-CCR2 antibody prevented the recruitment of CCR2^+^ monocytes to inflammatory sites, resulting in a reduction in CCR2^+^ MoMΦ. Consistent with our trajectory analysis, anti-CCR2 antibody treatment reduced CCR2^+^ macrophages by inhibiting monocyte recruitment, rather than by acting directly on existing CCR2^+^ macrophages (Fig. S6).

These results demonstrate that Treg depletion combined with PD-1 inhibition successfully recapitulates the immune microenvironment remodeling characteristic of clinical CIP^+^ patients: reduced AMs alongside a synchronized expansion of CD8^+^ T cells and CCR2^+^ MoMΦ.

### Pathogenic CCR2^+^ MoMΦ exhibited core pro-inflammatory functions and responded to type II interferon signaling

**Fig. 3.**
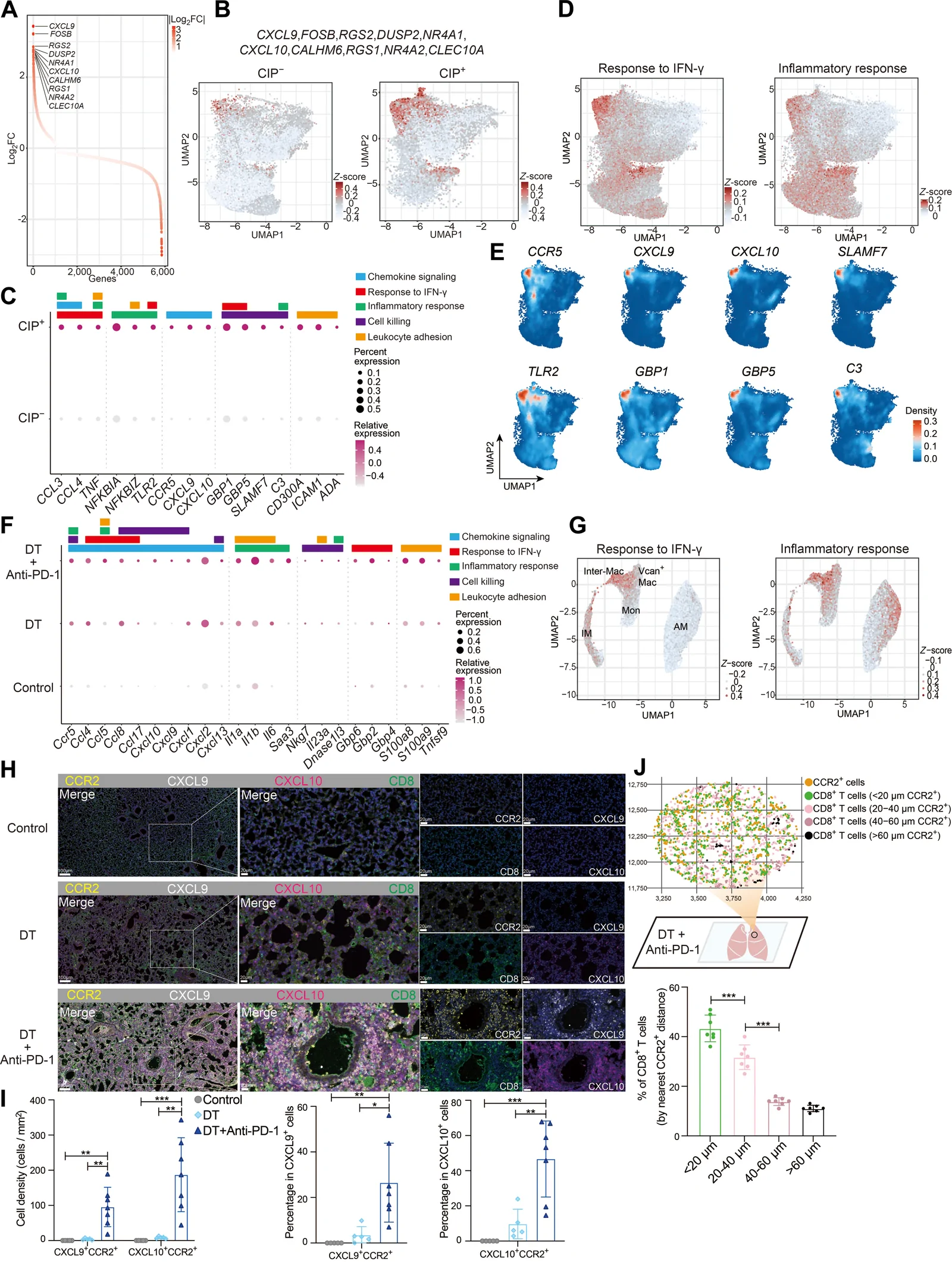
Expanded CCR2^+^CXCL9/10^+^ MoMΦ exhibit a pro-inflammatory and IFN-γ-responsive transcriptional signature in CIP^+^ patients and the mouse model. (A) Volcano plot showed the differentially expressed genes (adjusted *P* value < 0.05 and |log_2_FC| > 1) in monocytes/macrophages from BALF of CIP^+^ and CIP^−^ patients. (B) *Z*-score feature plot showed the top 10 up-regulated genes in CIP^+^ BALF monocytes/macrophages (versus CIP^−^), with these genes selectively expressed in CXCL9/10^+^ macrophages. (C) Expression levels of specific genes, chosen from the GO terms enriched within the set of up-regulated genes in the monocytes/macrophages of BALF from CIP^+^ patients (adjusted *P* < 0.01). Dot size corresponds to the proportion of cells, and color indicates relative expression. (D) *Z*-score feature plots of enriched genes associated with response to IFN-γ and inflammatory response pathways in BALF monocytes/macrophages. (E) Density plot of specific genes shown in (C). (F) Expression levels of specific genes, chosen from the GO terms enriched within the set of up-regulated genes in the monocytes/macrophages of lungs from *Foxp3-DTR-GFP* mice (adjusted *P* < 0.01). Dot size corresponds to the fraction of cells, and color indicates average expression. (G) *Z*-score feature plots displaying enriched genes involved in response to IFN-γ and inflammatory response pathways in mouse lung monocyte/macrophage. (H to J) Multiplex immunofluorescence analysis of mouse lung tissue (LLC1-Luc). The staining panel included antibodies against CCR2, CXCL9, CXCL10, and CD8α. (H) Representative images of lung tissues from each group. Insets display magnified views of the areas marked by white dashed lines, highlighting the coexpression of CCR2 with CXCL9 and CXCL10, as well as the spatial proximity between CCR2^+^ cells and CD8^+^ T cells. Magnification: ×10 and ×40. (I) Quantification of the CXCL9^+^CCR2^+^ and CXCL10^+^CCR2^+^ cell density (cells/mm^2^) and the percentage of these double-positive cells among CXCL9^+^ or CXCL10^+^ positive cells for each group. (J) Representative spatial map showing CCR2^+^ cells and CD8^+^ T cells in DT + anti-PD-1-treated lung tissue, with CD8^+^ T cells color-coded by their nearest-neighbor distance to CCR2^+^ cells (<20 μm, 20 to 40 μm, 40 to 60 μm, >60 μm). Bar plot quantifying the percentage of CD8^+^ T cells in each distance bin based on the nearest CCR2^+^ cell distance. Control (*n* = 5), DT (*n* = 5), DT + anti-PD-1 (*n* = 7); one-way ANOVA with Tukey’s test was used; significance is shown as *P < 0.05, **P < 0.01, ***P < 0.001. C3, complement component 3; CALHM6, calcium homeostasis modulator 6; CCR5, C-C motif chemokine receptor 5; CLEC10A, C-type lectin domain-containing 10A; DUSP2, dual-specificity phosphatase 2; FOSB, FosB proto-oncogene, AP-1 transcription factor subunit; GBP1, guanylate-binding protein 1; GBP5, guanylate-binding protein 5; GO, Gene Ontology; IFN-γ, interferon-γ; NR4A1, nuclear receptor subfamily 4 group A member 1; NR4A2, nuclear receptor subfamily 4 group A member 2; RGS1, regulator of G-protein signaling 1; RGS2, regulator of G-protein signaling 2; SLAMF7, signaling lymphocytic activation molecule family member 7; TLR2, Toll-like receptor 2.

To further analyze the transcriptional features of CIP-associated monocytes/macrophages, we performed differential gene expression analysis using BALF scRNA-seq data. The top 10 genes up-regulated in monocytes/macrophages from CIP^+^ versus CIP^−^ patients were preferentially expressed in CXCL9/10^+^ macrophages (Fig. 3A and B), suggesting that the expansion and activation of CXCL9/10^+^ macrophages represent a primary difference in monocytes/macrophages between CIP^+^ and CIP^−^ groups. GO enrichment analysis of up-regulated genes in the monocytes/macrophages of CIP^+^ patients revealed that several biologically relevant immune-inflammatory programs were among the most significantly affected, including chemokine signaling, response to IFN-γ, inflammatory response, cell killing, and leukocyte adhesion (Fig. 3C and Fig. S7A). Representative up-regulated genes contributing to these programs included chemokines and chemokine receptors such as *CCL3*, *CCL4*, *CCR5*, *CXCL9*, and *CXCL10*; inflammatory and interferon-response genes such as tumor necrosis factor (*TNF*), Toll-like receptor 2 (*TLR2*), guanylate binding protein 1 (*GBP1*), *GBP5*, and complement component 3 (*C3*); the cell killing-associated gene signaling lymphocytic activation molecule family member 7 (*SLAMF7*); and genes associated with leukocyte adhesion, including *CD300A*, intercellular adhesion molecule 1 (*ICAM1*), and adenosine deaminase (*ADA*) (Fig. 3C). These inflammatory programs and representative genes were predominantly localized to the CXCL9/10^+^ macrophages (Fig. 3D and E and Fig. S7B and C). Reverse transcription polymerase chain reaction (RT-PCR) further confirmed significantly increased expression of *CXCL9*, *CXCL10*, *CCR5*, *CCL3*, and *CCL4* in the BALF of CIP^+^ patients compared to CIP^−^ patients (Fig. S7D).

In addition to CXCL9/10^+^ inflammatory macrophages, scRNA-seq analysis also revealed a distinct PLTP^+^ macrophage population that was expanded in CIP (Fig. 1D). We performed a DEG comparison between PLTP^+^ and CXCL9/10^+^ macrophages. PLTP^+^ macrophages showed higher expression of lipid-associated genes [e.g., *PLTP*, apolipoprotein C1 (*APOC1*), *APOE*, fatty acid binding protein 4 (*FABP4*)] and enrichment of lipid-related processes, including lipid transport, fatty acid metabolic processes, and cholesterol efflux (Fig. S7E and F). In contrast, CXCL9/10^+^ macrophages were enriched for immune-response processes, including T cell activation, positive regulation of cytokine production, and response to type II interferon (Fig. S7F).

Next, we analyzed the transcriptional profiles of monocytes/macrophages in the lungs of *Foxp3-DTR-GFP* mice. Consistent with our expectations, monocytes/macrophages from DT + anti-PD-1-treated mice exhibited functional similarities to those from CIP^+^ patients, characterized by marked up-regulation of multiple pro-inflammatory processes (Fig. 3F and Fig. S7G). The response to IFN-γ, leukocyte adhesion, and cell killing pathways were primarily localized to Inter-Mac (Fig. 3G and Fig. S7H). The inflammatory response was localized to Inter-Mac and partially to AM (Fig. 3G). Chemokine-mediated signaling pathways were mainly localized to IM (Fig. S7H). We observed that genes associated with chemokine signaling, inflammatory response, and cell killing (*Cxcl9*, *Cxcl10*, *Ccr5, Il1b*) were primarily expressed in Inter-Mac, IM, and partially in AM. Notably, expression of these genes was increased within Inter-Mac in DT + anti-PD-1-treated mice compared to control and DT mice (Fig. S7I). These data indicate that CXCL9/10^+^ macrophages in CIP represent an activated population with enhanced potential for inflammatory cytokine/chemokine production and responsiveness to IFN-γ signaling.

We subsequently employed multiplex immunofluorescence staining to validate the colocalization of CCR2 with CXCL9 and CXCL10, and to assess the spatial relationship between CCR2^+^ cells and CD8^+^ T cells. Multiplex immunofluorescence analysis revealed a significant increase in the absolute abundance and frequency of CXCL9^+^CCR2^+^ and CXCL10^+^CCR2^+^ macrophages concentrated around peribronchovascular bundles in the lungs of DT + anti-PD-1-treated mice bearing LLC1-Luc tumors compared to control and DT mice (Fig. 3H and I). The majority of CXCL9^+^ and CXCL10^+^ cells coexpressed CCR2 and were identified as macrophages in DT + anti-PD-1-treated mouse lungs (Fig. 3I). Within DT + anti-PD-1-treated mouse lungs, nearest-neighbor distance analysis showed that CD8^+^ T cells were most frequently located within 0 to 20 μm of CCR2^+^ cells, with a marked decrease in the proportion of CD8^+^ T cells at 20 to 40 μm and 40 to 60 μm and no further increase beyond 60 μm (Fig. 3J). These findings indicate a short-range enrichment of CD8^+^ T cells in the immediate vicinity of CCR2^+^ macrophages, suggesting potential local interactions between these populations.

Furthermore, using the PySCENIC pipeline, which defines TF modules based on their coexpression with target genes, we uncovered a unique transcriptional regulatory network that characterizes activated CXCL9/10^+^ macrophages in CIP^+^ patients. TFs regulated by IFN-γ signaling, notably STAT1 and interferon regulatory factor 1 (IRF1), showed significant enrichment within activated CXCL9/10^+^ macrophages (Fig. S8A). RNA expression of *STAT1* and *IRF1* was confirmed within CXCL9/10^+^ macrophages (Fig. S8B). To validate this, we analyzed STAT1 protein expression and phosphorylation status in BALF from CIP^+^ and CIP^−^ patients. CIP^+^ patients exhibited significantly enhanced STAT1 expression and phosphorylation levels compared with CIP^−^ patients (Fig. S8C), indicating activation of the IFN-γ signaling pathway. PySCENIC analysis of murine monocyte/macrophage gene regulatory networks similarly revealed enrichment of IFN-γ downstream TFs Stat1 and Irf7 in Inter-Mac from DT + anti-PD-1-treated mice (Fig. S8D). RNA expression of *Stat1* and *Irf7* was further confirmed predominantly in Inter-Mac (Fig. S8E). In vivo validation in mice confirmed significantly enhanced Stat1 protein expression and phosphorylation levels in the lungs of DT + anti-PD-1-treated mice compared with control or DT mice (Fig. S8F). In vitro, inhibition of Stat1 activation using the Janus kinase 1/2 (JAK1/2) inhibitor ruxolitinib or knockdown of *Stat1* expression in BMDMs significantly reduced IFN-γ-induced Stat1 phosphorylation and/or expression (Fig. S8G), and significantly suppressed IFN-γ-induced *Cxcl9* and *Cxcl10* mRNA expression (Fig. S8H) as well as Cxcl9 and Cxcl10 protein expression (Fig. S8I). These results suggest the IFN-γ–STAT1 signaling axis as a key driver of CXCL9/10^+^ macrophage expansion and emergence in CIP.

### IFN-γ was primarily secreted by CD8^+^ T cells

**Fig. 4.**
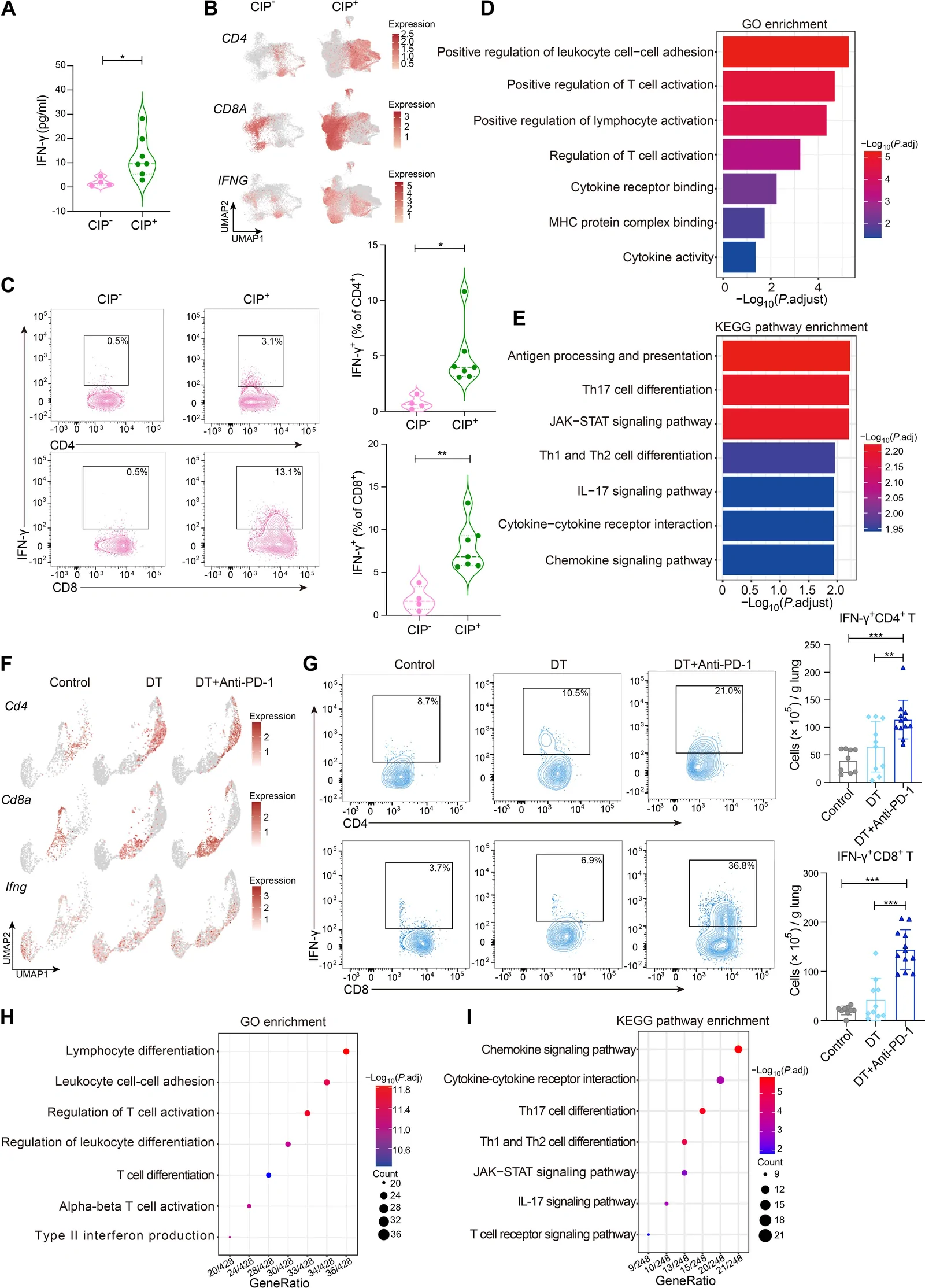
T cell populations exhibit functional similarity in CIP^+^ patients and the mouse model, with CD8^+^ T cells serving as the primary source of IFN-γ. (A) BALF from CIP^+^ patients exhibited significantly elevated IFN-γ levels compared to CIP^−^ patients (2-tailed Mann–Whitney test; significance is shown as *P < 0.05; CIP^−^, *n* = 4; CIP^+^, *n* = 7). (B) UMAP analysis of *CD4*, *CD8A*, and *IFNG* expression in 66,186 BALF NK/T cells demonstrated CD8^+^ T cell expansion and *IFNG* enrichment in the CD8^+^ T cluster in CIP^+^ patients relative to CIP^−^ controls. (C) Percentages of IFN-γ^+^CD4^+^ and IFN-γ^+^CD8^+^ T cells in BALF were analyzed by flow cytometry (2-tailed Mann–Whitney test; significance is shown as *P < 0.05, **P < 0.01; CIP^−^, *n* = 4; CIP^+^, *n* = 7). (D and E) GO (D) and KEGG (E) pathway enrichment analyses were performed on genes up-regulated in CIP^+^ BALF T cells compared to CIP^−^ controls. Genes used in the analysis were identified via Seurat differential expression analysis with adjusted *P* < 0.05, |log_2_FC| > 1. (F) UMAP analysis of 4,982 murine lung NK/T cells revealed CD8^+^ T cell expansion and *Ifng* enrichment in CD8^+^ T cells following DT + anti-PD-1 treatment, based on *Cd4*, *Cd8*α, and *Ifng* expression profiling. (G) Absolute counts of IFN-γ^+^CD4^+^ and IFN-γ^+^CD8^+^ T cells in murine lungs (LLC1-Luc) were analyzed by flow cytometry. Data pooled from 4 independent experiments. Control (*n* = 9), DT (*n* = 10), DT + anti-PD-1 (*n* = 12). One-way ANOVA with Tukey’s test was used; significance is shown as *P < 0.05, **P < 0.01, ***P < 0.001. (H and I) GO (H) and KEGG (I) pathway enrichment of up-regulated genes in lung T cells (DT + anti-PD-1 versus control). Genes selected by Seurat (adjusted *P* < 0.05, |log_2_FC| > 1). JAK–STAT, Janus kinase–signal transducer and activator of transcription; KEGG, Kyoto Encyclopedia of Genes and Genomes.

Pathway analysis revealed that CCR2^+^CXCL9/10^+^ MoMΦ displayed features of IFN-γ pathway activation, suggesting that activation of IFN-γ signaling within the lung tissue may regulate the differentiation or functional activity of this macrophage cluster. Consistent with this possibility, we detected increased IFN-γ levels in the BALF of CIP^+^ patients compared to CIP^−^ patients (Fig. 4A). To identify the cellular source of IFN-γ, we analyzed the BALF scRNA-seq dataset and observed constitutive expression of *IFNG* mRNA primarily within CD8^+^ T and CD4^+^ T cell clusters (Fig. S9A). Furthermore, *IFNG* mRNA expression was higher in CIP^+^ patients than in CIP^−^ patients (Fig. 4B). Intracellular flow cytometry confirmed elevated IFN-γ production by both CD4^+^ and CD8^+^ T cells in the BALF of CIP^+^ patients compared with CIP^−^ patients. Among the T cell populations, CD8^+^ T cells showed a more pronounced increase in IFN-γ expression than CD4^+^ T cells (Fig. 4C). Additionally, intracellular flow cytometry confirmed increased production of perforin 1 (PRF1) and granzyme B (GZMB) by CD4^+^ and CD8^+^ T cells in the BALF of CIP^+^ patients compared with CIP^−^ patients (Fig. S9B and C). GO and KEGG pathway analyses indicated that enhanced leukocyte adhesion, T cell activation, antigen processing and presentation, and JAK–STAT signaling were putative hallmarks of T cell activation in CIP (Fig. 4D and E). These data highlight dysregulated T cell activation in CIP.

We next analyzed the cellular source of IFN-γ in the CIP mouse model. scRNA-seq data showed constitutive *Ifng* mRNA expression within CD4^+^ T cell, CD8^+^ T cell, and NK-cell clusters (Fig. 4F and Fig. S9D). Intracellular flow cytometry showed that the absolute counts of IFN-γ^+^CD4^+^ and IFN-γ^+^CD8^+^ T cells in the lungs were higher in DT + anti-PD-1-treated mice than in control or DT-treated mice, with IFN-γ^+^CD8^+^ T cells showing a more pronounced increase than IFN-γ^+^CD4^+^ T cells (Fig. 4G). The absolute counts of IFN-γ^+^ NK cells remained low and did not differ significantly among the 3 groups (Fig. S9E). Furthermore, intracellular flow cytometry confirmed that the absolute counts of PRF1^+^CD4^+^, PRF1^+^CD8^+^, GZMB^+^CD4^+^, and GZMB^+^CD8^+^ T cells in the lungs were increased in DT + anti-PD-1-treated mice compared with control mice (Fig. S9F and G). Consistently, the absolute counts of inducible T cell costimulator (ICOS)^+^CD4^+^ and ICOS^+^CD8^+^ T cells in the lungs were significantly higher in both DT- and DT + anti-PD-1-treated mice than in control mice, with DT + anti-PD-1-treated mice showing a greater increase than DT mice (Fig. S9H). Notably, functional enrichment analysis of lung T cell populations in the DT + anti-PD-1-treated mice showed similarities to those in CIP^+^ patients and was also characterized primarily by aberrant T cell activation (Fig. 4H and I).

Collectively, these findings identify activated CD8^+^ T cells as a major cellular source of elevated pulmonary IFN-γ in both patients with CIP and the mouse model.

### CD8^+^ T cells interacted with CCR2^+^ MoMΦ via the IFN-γ and CXCL9/CXCL10–CXCR3 signaling axis

**Fig. 5.**
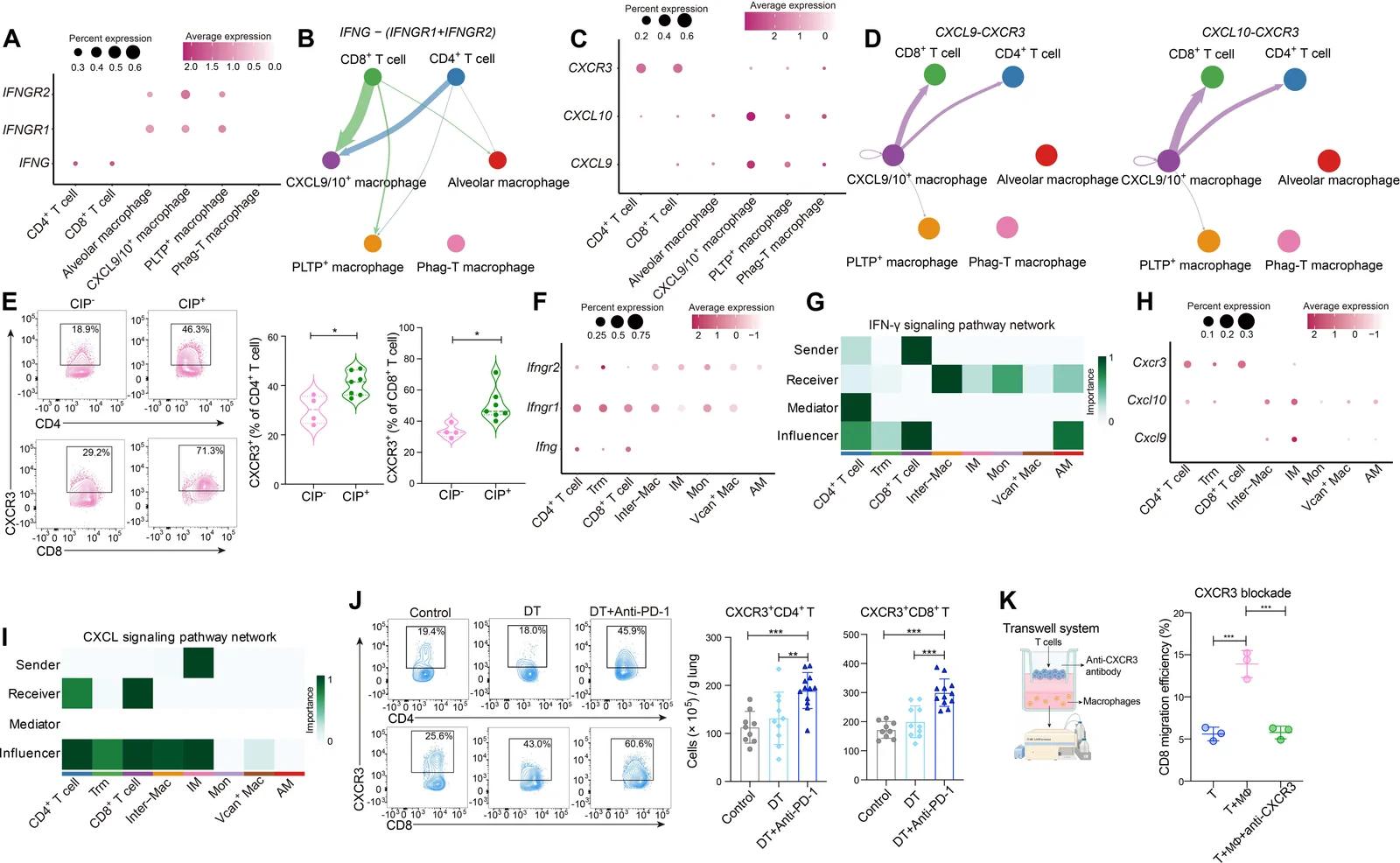
The communication between T cells and CCR2^+^CXCL9/10^+^ MoMΦ is mediated by the IFN-γ and CXCL9/CXCL10–CXCR3 signaling axis. (A) CellChat analysis of BALF scRNA-seq data predicted T cell-to-macrophage signaling via IFN-γ, and the dot plot showed the expression distribution of ligands and receptors in the IFN-γ pathway across T cells and macrophages. (B) A circular plot summarized the inferred T cell–macrophage communication network mediating IFN-γ signaling. (C) CellChat analysis of BALF scRNA-seq data predicted macrophage-to-T cell signaling via the CXCL pathway (CXCL9/CXCL10–CXCR3 axis), with the dot plot showing expression distributions of ligands and receptors in the CXCL signaling network across macrophages and T cells. (D) Circular plots visualized the inferred macrophage–T cell communication network mediating CXCL signaling. (E) Percentages of CXCR3^+^CD4^+^ and CXCR3^+^CD8^+^ T cells in BALF were analyzed by flow cytometry (2-tailed Mann–Whitney test; significance is shown as *P < 0.05; CIP^−^, *n* = 4; CIP^+^, *n* = 7). (F) CellChat analysis of mouse lung scRNA-seq data predicted T cell-to-macrophage signaling via IFN-γ. The dot plot displayed the expression distribution of ligands and receptors in the IFN-γ pathway across T cells and macrophages. (G) The heatmap visualized the relative importance of T cells and macrophage states derived from computational analysis of the IFN-γ signaling network. (H) CellChat analysis of mouse lung scRNA-seq data predicted macrophage-to-T cell signaling via the CXCL pathway (Cxcl9/Cxcl10–Cxcr3 axis). The dot plot displayed expression distributions of associated ligands and receptors in the CXCL signaling network across macrophage and T cell populations. (I) The heatmap visualized the relative importance of T cells and macrophage states derived from computational analysis of the CXCL signaling network. (J) Absolute counts of CXCR3^+^CD4^+^ and CXCR3^+^CD8^+^ T cells in murine lungs (LLC1-Luc) were analyzed by flow cytometry. Data pooled from 4 independent experiments. Control (*n* = 9), DT (*n* = 10), DT + anti-PD-1 (*n* = 12). (K) Quantitative analysis of pulmonary CD8^+^ T cell migration in the Transwell assay following in vitro CXCR3 blockade of T cells before addition to the upper chamber, with peritoneal macrophages placed in the lower chamber (*n* = 3 per group). One-way ANOVA with Tukey’s test was used; significance is shown as **P < 0.01 and ***P < 0.001. CXCR3, C-X-C motif chemokine receptor 3; MΦ, macrophages.

To investigate cellular crosstalk between macrophages and T cells in CIP, we employed CellChat to infer intercellular communication networks. IFN-γ signaling represented the predominant pathway originating from predicted CD4^+^ and CD8^+^ T cells, with CXCL9/10^+^ macrophages identified as the primary recipients. Furthermore, CXCL9/10^+^ macrophages were predicted to initiate CXCL signaling, which was predominantly received by CD4^+^ and CD8^+^ T cells (Fig. S10). These predictions aligned with GO enrichment analyses demonstrating the responsiveness of CXCL9/10^+^ macrophages to IFN-γ signaling (Fig. 3D and Fig. S7A). Consequently, we focused on IFN-γ and CXCL signaling. We first examined *IFNG*, *IFNGR1*, and *IFNGR2* mRNA expression. *IFNG* mRNA was selectively expressed in CD4^+^ and CD8^+^ T cells. *IFNGR1* and *IFNGR2* were selectively expressed in AMs, CXCL9/10^+^ macrophages, and PLTP^+^ macrophages. In particular, *IFNGR2* was most highly expressed in CXCL9/10^+^ macrophages compared with AMs and PLTP^+^ macrophages (Fig. 5A). CellChat pathway network analysis predicted CD4^+^ and CD8^+^ T cells as the principal sources of ligands, while CXCL9/10^+^ macrophages were the main receivers of IFN-γ signals. CD8^+^ T cells exhibited a higher communication probability than CD4^+^ T cells (Fig. 5B and Fig. S11A). CXCL9/10^+^ macrophages were predicted to act as sources of CXCL ligands, signaling to CD4^+^ and CD8^+^ T cells via the CXCR3 receptor (Fig. 5C and D). Again, CD8^+^ T cells showed a higher communication probability than CD4^+^ T cells (Fig. S11B). Flow cytometry analysis revealed a significantly higher proportion of CXCR3^+^CD4^+^ and CXCR3^+^CD8^+^ T cells in the BALF of CIP^+^ patients compared to CIP^−^ patients. Notably, the increase in CXCR3^+^ frequency was more pronounced in CD8^+^ T cells than in CD4^+^ T cells (Fig. 5E).

We next used CellChat to infer macrophage–T cell communication in the *Foxp3-DTR-GFP* mice. IFN-γ signaling was primarily emitted by CD4^+^ and CD8^+^ T cells, with inflammatory Inter-Mac identified as the main receiver (Fig. 5F and G and Fig. S12). CD8^+^ T cells demonstrated a higher communication probability than CD4^+^ T cells (Fig. S13A). IMs were predicted as the dominant source of CXCL ligands, signaling to CD4^+^ and CD8^+^ T cells via the CXCR3 receptor (Fig. 5H and I and Fig. S12). CD8^+^ T cells again showed a higher communication probability than CD4^+^ T cells (Fig. S13B). Flow cytometry confirmed that the absolute counts of CXCR3^+^CD4^+^ T cells and CXCR3^+^CD8^+^ T cells in the lungs were significantly higher in DT + anti-PD-1-treated mice than in DT or control mice (Fig. 5J).

To determine the role of CXCR3, CXCL9, and CXCL10 in T cell migration toward macrophages, we employed a Transwell migration assay system. Lung-derived T cells and peritoneal macrophages were isolated from DT + anti-PD-1-treated mice. T cells were seeded in the upper chamber, whereas macrophages were placed in the lower chamber. Migrated CD8^+^ T cells in the lower chamber were quantified by flow cytometry (Fig. S14A). The presence of macrophages significantly increased CD8^+^ T cell migration, whereas CXCR3 blockade on T cells significantly reduced this macrophage-directed migration (Fig. 5K). Furthermore, selective blockade of either CXCL9 or CXCL10 significantly reduced CD8^+^ T cell migration toward macrophages, with CXCL10 blockade exerting a more pronounced inhibitory effect than CXCL9 blockade. Combined blockade of both CXCL9 and CXCL10 resulted in a moderate amplification of this inhibitory effect (Fig. S14B). To further investigate the contribution of CXCL9 and CXCL10 to T cell migration toward macrophages, we supplemented otherwise identical chemotaxis assays with recombinant murine CXCL9 (rmCXCL9) or CXCL10 (rmCXCL10). Both chemokines significantly enhanced CD8^+^ T cell migration compared with medium-only controls, with rmCXCL10 exhibiting greater chemotactic potency than rmCXCL9. Moreover, combined administration of both chemokines elicited a more pronounced migratory response (Fig. S14C). Collectively, these findings support a reciprocal communication circuit between activated T cells and inflammatory macrophages in CIP, in which T cell-derived IFN-γ is predicted to stimulate inflammatory macrophages, whereas macrophage-derived CXCL9 and CXCL10 promote the migration of CXCR3^+^CD8^+^ T cells.

### Pharmacological inhibition of either CCR2/CCR5 or CXCR3 markedly attenuated T cell-mediated inflammation

Given that CCR5 appeared to be highly expressed by the same pathogenic CCR2^+^CXCL9/10^+^ MoMΦ (Fig. 3E), which also showed high expression of pro-inflammatory chemokines critical for recruiting CCR2^+^, CCR5^+^, and CXCR3^+^ cells (CXCL9/10, CCL3/4, CCL2), we next sought to clarify the relative contribution of CCR2 versus CCR5 to the recruitment of pathogenic monocytes/macrophages in CIP. In our human BALF scRNA-seq datasets, cluster-level comparisons revealed that CCR2/CCR5 and their major ligands, together with CXCR3 ligands, displayed higher expression within the pathogenic CCR2^+^CXCL9/10^+^ MoMΦ in CIP^+^ patients than in CIP^−^ patients. Importantly, the magnitude of CCR2 and CCL2 up-regulation exceeded that of CCR5 and its ligands (Fig. S15A). A similar pattern was observed in the mouse model, where DT + anti-PD-1-treated mice showed higher expression of these receptors/ligands than controls within the corresponding myeloid clusters, with CCR2 in Inter-Mac and CCL2 in IMs showing the prominent increases (Fig. S15B). Together, these human and mouse data suggested that CCL2–CCR2 likely serves as a principal recruitment axis, while CCR5 may act as a parallel or complementary pathway during inflammation.

We then performed a pharmacological dissection of these recruitment pathways in the DT + anti-PD-1-treated model. Compared with selective CCR2 or CCR5 inhibition, CVC, a dual CCR2/CCR5 antagonist, provided stronger protection against pneumonitis, as assessed by lung CT, histologic injury scores, and acute lung injury indices (BALF protein, BALF cell counts, and wet/dry ratio). CVC also achieved superior tumor control, while selective CCR2 inhibition outperformed selective CCR5 inhibition in antitumor efficacy (Fig. S15C to I). Together, these findings support the notion that both CCR2 and CCR5 contribute to pathogenic myeloid recruitment/maintenance, and that dual blockade more effectively restrains the inflammatory chemokine network.

**Fig. 6.**
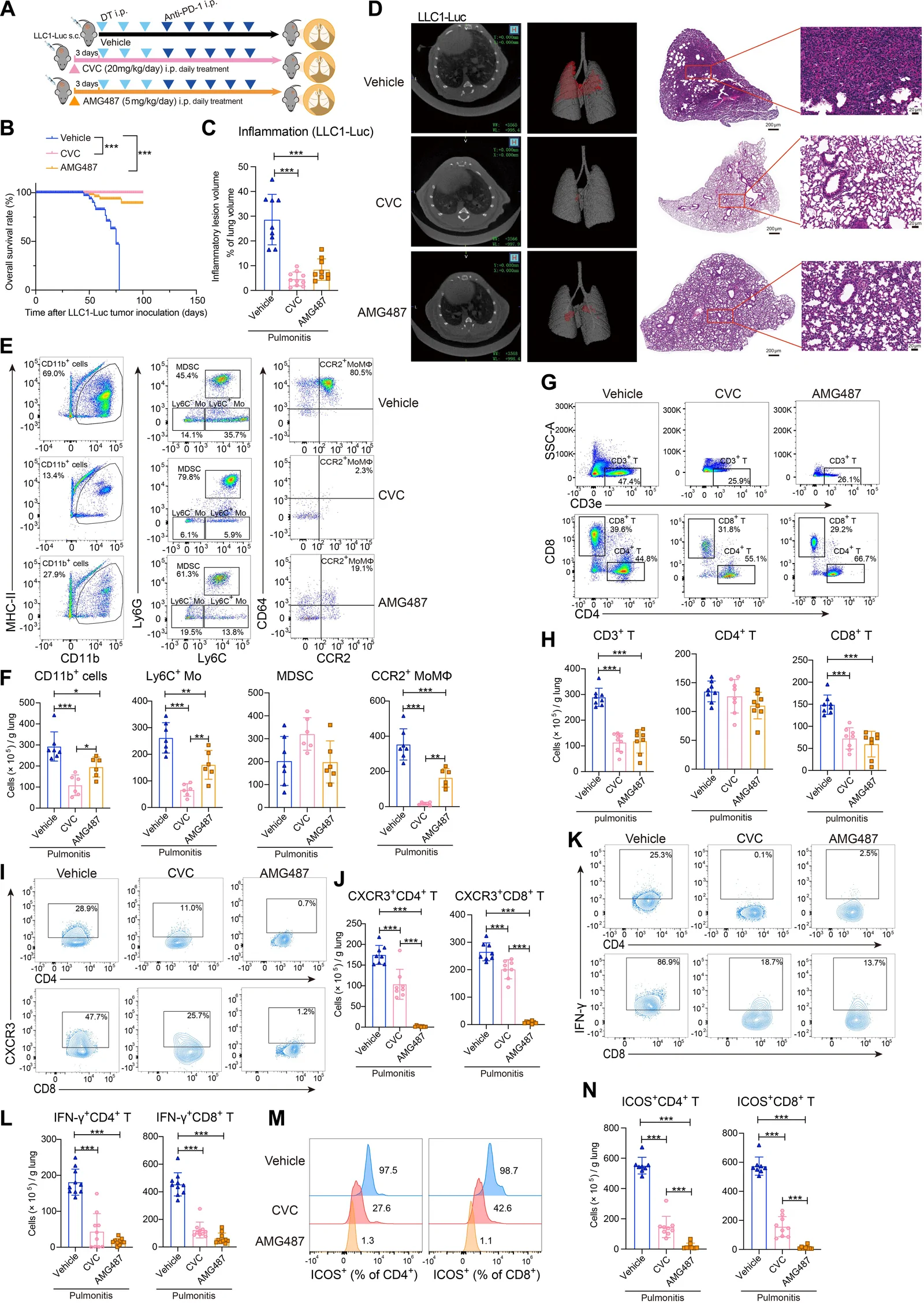
CCR2/CCR5 or CXCR3 antagonists alleviate pulmonary T cell inflammation in DT + anti-PD-1-treated mice. (A) LLC1-Luc-bearing mice developed CIP following combinatorial DT and anti-PD-1. CIP mice received CVC (CCR2/CCR5 antagonist) or AMG487 (CXCR3 antagonist) starting 3 d prior to initial DT administration and continuing throughout the experimental period. Treatment groups were labeled: Vehicle, DT + anti-PD-1 + PBS; CVC, DT + anti-PD-1 + CVC; AMG487, DT + anti-PD-1 + AMG487. (B) Kaplan–Meier survival curves of CIP mice receiving CVC or AMG487 versus nontreatment. Data represent 4 experimental replicates (*n* = 10 per group). Log-rank test was used; significance is shown as *P < 0.05, ***P < 0.001. (C) Quantification of inflammatory foci in lung tissue across treatment groups revealed that both CVC and AMG487 significantly attenuated pulmonary inflammation. (D) Multimodal assessment of CIP in LLC1-Luc-bearing mice (*n* = 10 per group): micro-CT (left), 3D-rendered inflammatory foci (red) within lung parenchyma (gray; middle), and H&E histopathology (right). (E and F) Flow cytometry analysis of pulmonary CD11b^+^ cells, Ly6C^+^ Mo, MDSCs, and Ly6C^+^CCR2^+^ MoMΦ following CVC or AMG487 treatment. Representative images (E) and quantitative analysis of each cell population (F). Data pooled from 2 independent experiments: vehicle (*n* = 7), CVC (*n* = 6), AMG487 (*n* = 6). (G to N) Representative images (G, I, K, and M) and quantitative analysis (H, J, L, and N) of pulmonary T cell clusters (gated on live CD45^+^CD3^+^) by flow cytometry, including CD3^+^ T, CD4^+^ T, CD8^+^ T, CXCR3^+^CD4^+^ T, CXCR3^+^CD8^+^ T, IFN-γ^+^CD4^+^ T, IFN-γ^+^CD8^+^ T, ICOS^+^CD4^+^ T, and ICOS^+^CD8^+^ T cells. Data pooled from 3 independent experiments (*n* = 8 to 9 per group). One-way ANOVA with Tukey’s test was used; significance denoted as *P < 0.05, **P < 0.01, ***P < 0.001. CVC, cenicriviroc; ICOS, inducible T cell costimulator.

Based on this mechanistic and pharmacological dissection of the upstream myeloid recruitment module, we investigated whether pharmacologically inhibiting pro-inflammatory monocyte migration or targeting the CXCR3 signaling axis could ameliorate the CIP phenotype. *Foxp3-DTR-GFP* mice were treated daily with CVC (20 mg kg^−1^ day^−1^) or the small-molecule CXCR3 inhibitor AMG487 (5 mg kg^−1^ day^−1^) via intraperitoneal injection starting 3 d prior to the first DT administration and continuing throughout CIP induction with DT and anti-PD-1 treatment (Fig. 6A). Compared with the vehicle group, both CVC and AMG487 treatments significantly prolonged the survival of DT + anti-PD-1-treated mice (Fig. 6B), which succumbed to autoimmune events as previously established [46]. Notably, micro-CT combined with lung histology revealed that both treatments effectively attenuated the pneumonitis phenotype compared to vehicle group, as evidenced by fewer centrilobular nodules, reduced inflammatory cell infiltration in the lung parenchyma and alveolar spaces, and decreased BALF protein levels, BALF cell counts, and lung wet-to-dry weight ratios (Fig. 6C and D and Fig. S16A and B). Tumor growth curves showed that, in the CVC group, 7 of 10 mice achieved durable tumor control without evidence of rebound, whereas 3 of 10 mice developed late tumor regrowth; in the AMG487 group, 6 of 10 mice maintained tumor control, while 4 of 10 mice displayed tumor regrowth (Fig. S16C and D).

Flow cytometry analysis of lung immune cell clusters after treatment showed that CVC exerted a more pronounced impact on myeloid populations than did AMG487 in DT + anti-PD-1-treated mice. Both CVC and AMG487 significantly reduced the absolute counts of CD11b^+^ cells, Ly6C^+^ Mo, and CCR2^+^ MoMΦ in pneumonitic lungs, but the magnitude of reduction was consistently greater with CVC (Fig. 6E and F). Moreover, only CVC significantly decreased the absolute counts of CCR2^+^ IMs, whereas AMG487 had no detectable effect on this population (Fig. S16E and F). CVC also showed a trend toward increasing MDSC counts, although this did not reach statistical significance (Fig. 6E and F). With respect to lymphoid populations, both inhibitors significantly decreased the absolute counts of CD3^+^ and CD8^+^ T cells while having minimal impact on total CD4^+^ T cell counts (Fig. 6G and H). Notably, although CVC and AMG487 both significantly reduced CXCR3^+^CD4^+^ and CXCR3^+^CD8^+^ T cells, the reduction was more pronounced with AMG487 (Fig. 6I and J), in line with its role as a CXCR3 antagonist. Consistently, both inhibitors significantly lowered the absolute counts of IFN-γ^+^CD4^+^ and IFN-γ^+^CD8^+^ T cells (Fig. 6K and L); AMG487-treated mice showed numerically lower counts than did CVC-treated mice, although this difference did not reach statistical significance. CVC and AMG487 also significantly decreased ICOS^+^ T cell counts, and AMG487 further reduced this population compared with CVC (Fig. 6M and N). ICOS plays a critical role in the production of IL-2, IL-4, IL-5, and IFN-γ by newly activated T cells. It also promotes T cell-dependent antibody responses in vivo and is implicated in the pathogenesis of autoimmune diseases [47]. Finally, both CVC and AMG487 markedly reduced the absolute counts of GZMB^+^ and PRF1^+^ T cells (Fig. S16G to J), indicating attenuation of cytotoxic effector programs in the lungs.

Collectively, these results demonstrate that both therapeutic strategies successfully suppressed the expansion and activation of pathogenic T cell clusters, and mitigated cytotoxicity-induced organ damage, and confirm the functional importance of MoMΦ and the CXCL9/10–CXCR3 signaling axis in CIP pathogenesis.

## Discussion

**Fig. 7.**
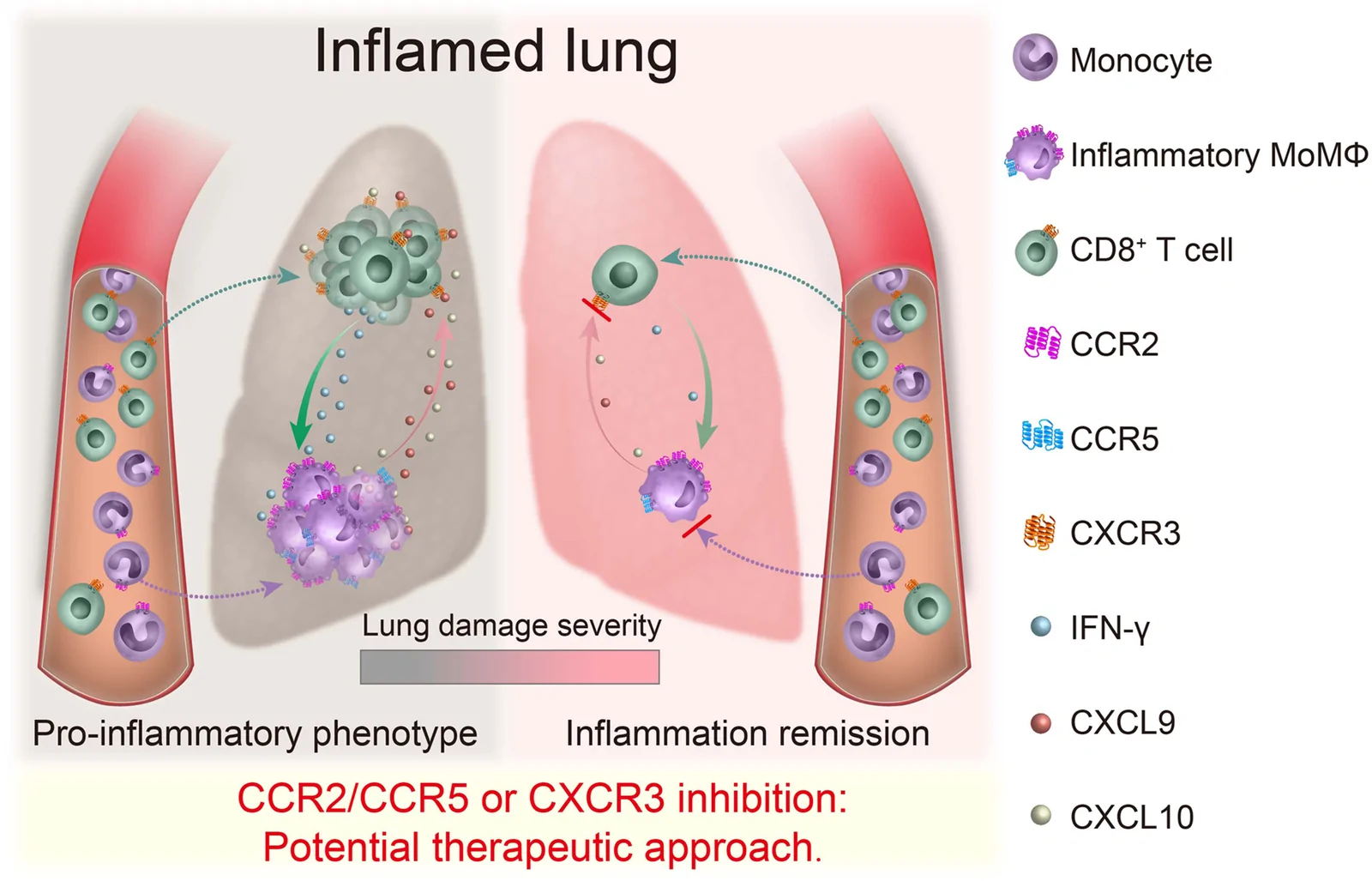
Schematic of the proposed mechanism. In CIP pathogenesis, CD8^+^ T cells interacted with CCR2^+^ MoMΦ through the IFN-γ and CXCL9/10–CXCR3 signaling axis. Both CCR2/CCR5 inhibitor (blocking CCR2^+^ monocyte recruitment) and CXCR3 inhibitor (disrupting CXCL9/10–CXCR3 signaling) effectively attenuated T cell-mediated inflammation, indicating these pathways as potential therapeutic targets.

In this study, we combined BALF profiling from patients with CIP and a *Foxp3-DTR-GFP* mouse model with anti-PD-1 treatment to dissect the pulmonary immune circuits underlying CIP. We found that CIP lungs were characterized by marked accumulation of CCR2^+^ MoMΦ and activated CD8^+^ T cells. scRNA-seq revealed an inflammatory CCR2^+^ MoMΦ state that was highly responsive to IFN-γ and enriched for chemokines such as CXCL9 and CXCL10. Spatial and functional analyses demonstrated close apposition and bidirectional crosstalk between CCR2^+^ MoMΦ and CD8^+^ T cells through the CXCL9/10–CXCR3 and IFN-γ axes, forming a short-range feed-forward circuit. Pharmacologic blockade of CCR2/CCR5 or CXCR3 selectively reduced pulmonary CCR2^+^ MoMΦ and effector T cells, attenuated lung inflammation, and improved survival while maintaining tumor control. Together, these findings identify a CCR2^+^ MoMΦ–T cell interaction axis as a central driver of CIP and highlight CCR2^+^ monocyte recruitment and CXCR3 signaling as promising therapeutic targets (Fig. 7).

Autoimmune diseases are characterized by a loss of immune self-tolerance. Treg cells represent one of the most critical T cell populations for maintaining immune self-tolerance and homeostasis, playing an indispensable role in autoimmunity [48]. Indeed, irAEs induced by ICIs may partially result from the depletion or functional impairment of immunosuppressive Treg cells [49]. Wild-type mice have been reported to be relatively resistant to developing overt irAEs after ICI therapy, reflecting a high threshold for breaking self-tolerance [50]. In this study, we selected *Foxp3-DTR-GFP* mice as a model for investigating CIP. This model leverages DT-induced Treg depletion to reduce immune self-tolerance. Subsequent administration of ICIs enables the evaluation of both antitumor efficacy and irAEs [26,51]. Using micro-CT, histological examination, and scRNA-seq, we comprehensively characterized pulmonary inflammation in this model for the first time. Radiographically, micro-CT revealed ground-glass opacities and centrilobular nodules. Histopathological analysis demonstrated dense immune cell infiltration within the parenchyma and alveolar spaces, accompanied by septal thickening. Furthermore, scRNA-seq identified remodeling of the immune microenvironment, characterized by expansion of T cells and CCR2^+^CXCL9/10^+^ MoMΦ. These features recapitulate the clinical presentation and dynamic immune microenvironment evolution observed in CIP patients. This model overcomes the limitations of obtaining lung tissue samples from CIP patients, providing a controllable platform for mechanistic studies.

Lung tissue harbors a highly heterogeneous macrophage population. This population primarily comprises long-lived AMs and IMs (collectively termed tissue-resident macrophages), alongside a constantly replenished, short-lived population of MoMΦ. These MoMΦ originate from recruited monocytes that, under the guidance of location-specific signals, progressively acquire the tissue-resident characteristics of the macrophages they replace [14,15]. CCR2 is recognized as a marker for short-lived MoMΦ in both humans and mice [52]. Our data demonstrate a significant reduction in resident AMs within the BALF of CIP^+^ patients, concomitant with a marked expansion of CCR2^+^CXCL9/10^+^ MoMΦ. A similar pattern was observed in the DT + anti-PD-1-induced pneumonitis model, where resident AMs and IMs were reduced, whereas CCR2^+^ MoMΦ were substantially increased. CCR2^+^ IMs showed a modest increase relative to control mice. Trajectory analysis of mouse lung scRNA-seq data, together with quantitative assessment of CCR2^+^, CCL2^+^, and double-positive cells across monocytes, intermediate macrophages, and IMs, supports a differentiation continuum from monocytes through CCR2^high^ intermediate macrophages to CCL2^high^ IMs, with progressive down-regulation of CCR2 and acquisition of CCL2-secreting capacity along this path. In vivo CCR2/CCR5 blockade reduced CCR2^+^ MoMΦ and CCR2^+^ IMs and ameliorated CIP, indicating that CCR2-lineage MoMΦ are key pathogenic effectors in this setting. These findings are consistent with lineage-tracing and scRNA-seq studies in other inflammatory contexts showing that recruited CCR2^+^ classical monocytes can differentiate into IMs while losing CCR2 and gaining chemokine expression, including CCL2 [53–55], and with reports from COVID-19 and cystic fibrosis, where massive influx of Ly6C^+^CCR2^+^ monocytes and replacement of resident macrophage pools contribute to lung pathology [42,56]. Together, our data support a model in which CCR2^+^ MoMΦ are initially recruited by preexisting CCL2 gradients and then become local CCL2 producers that sustain feed-forward recruitment of additional CCR2^+^ cells, thereby driving CIP development.

A remaining question is what initiates CCL2 up-regulation and the consequent accumulation of CCR2^+^ MoMΦ during PD-1 blockade. Although IFN-γ was a dominant effector cytokine in this setting, a previous study has suggested that IFN-γ alone is insufficient to robustly induce CCL2 in monocytes/macrophages without additional inflammatory cues [57]. Importantly, the CCL2 pool that fueled recruitment of CCR2^+^ cells may not have originated from a single cellular source. In our human CIP dataset, although CCL2 was prominently enriched in CXCL9/10^+^ macrophages, we also detected CCL2 expression in lung endothelial cells, implying that nonhematopoietic compartments might have contributed to an early chemokine milieu that permitted CCR2^+^ monocyte ingress. In parallel, a previous study has shown that PD-1 blockade can enhance CD4^+^ T cell activation with increased CD40 ligation (CD40L) expression [58], and that CD40L has been shown to promote CCL2 production in macrophages through canonical inflammatory transcriptional programs, including activator protein 1 (AP-1) and nuclear factor κB (NF-κB) [59–61]. Together, these observations support a model in which macrophage-intrinsic CCL2 induction and endothelial/stromal contributions may act in concert to initiate and amplify CCR2^+^ monocyte recruitment, subsequently feeding into the IFN-γ–CXCL9/10–CXCR3 amplification loop established by our data. Future studies testing this multicellular upstream circuit will further refine the initiating events of CIP.

Cytokine dysregulation is implicated in irAEs, including CIP [10]. Prior immunophenotyping studies reported significantly elevated levels of various proinflammatory chemokines/cytokines including CXCL9, CXCL10, CCL2, IFN-γ, IL-1β, IL-6, and TNF-α in the BALF of patients with PD-1/PD-L1 inhibitor-induced CIP [20]. Consistent with this, our scRNA-seq data also indicated a significant up-regulation of chemokine-mediated signaling pathways within monocyte/macrophage and T cell populations in both CIP^+^ patients and the mouse model. Within the myeloid compartment, the pathogenic CCR2^+^CXCL9/10^+^ MoMΦ cluster not only expressed high levels of CCR2 and its ligand CCL2 but also up-regulated CCR5 together with its ligands CCL3 and CCL4, chemokine axes known to be induced on inflammatory monocytes/macrophages and to contribute to their recruitment into inflamed tissues. These findings suggested that CCR2- and CCR5-dependent pathways might cooperate in driving monocyte/macrophage accumulation in CIP. Accordingly, we used our CIP mouse model to investigate how selectively blocking CCR2 or CCR5, as well as dual CCR2/CCR5 blockade with CVC, affects the development of CIP. In this setting, all 3 regimens attenuated lung inflammation, as reflected by improved CT imaging, lower histopathological scores, and reduced indices of acute lung injury (BALF protein concentration, BALF cell counts, and lung wet/dry ratio), with CVC conferring the most consistent and pronounced protective effects and superior tumor control compared with either selective CCR2 or CCR5 inhibition. Taken together with the more prominent up-regulation of CCR2 and CCL2 than CCR5 and its ligands in the pathogenic MoMΦ, we interpret these data as suggesting that the CCL2–CCR2 axis is the dominant driver of monocyte recruitment in CIP, whereas CCR5 functions as a parallel or compensatory pathway. In other inflammatory settings, CCR2–CCL2 signaling has been shown to mediate the early influx of Ly6C^+^ inflammatory monocytes, while CCR5 and its ligands participate in amplification and tissue retention of MoMΦ [62], providing a mechanistic framework for why dual CCR2/CCR5 blockade may achieve more complete interruption of pathogenic monocyte entry and persistence than selective inhibition of either receptor alone.

These CCR2^+^ MoMΦ exhibited a robust IFN-γ response, reflected in the significant up-regulation of multiple inflammatory mediators associated with the IFN-γ signaling pathway. Furthermore, this population displayed transcriptional signatures indicative of enhanced leukocyte adhesion, cytotoxic potential, and chemotactic activity. These pathways are directly linked to exacerbating local tissue damage, and IFN-γ pathway activation serves as a core driver for these processes [63]. Integrating scRNA-seq data with flow cytometry, we confirmed that IFN-γ is predominantly produced by T cells in both CIP^+^ patients and the mouse model. In vitro stimulation assays using CIP samples demonstrated polarization of T cells toward a type 1 phenotype with increased IFN-γ production [12]. Additionally, Franken et al. [21] identified pathogenic T helper 17.1 (Th17.1) cells capable of producing IFN-γ as being associated with CIP. Notably, we observed a significant increase in the proportion of both IFN-γ^+^CD4^+^ T cells and IFN-γ^+^CD8^+^ T cells in CIP^+^ patients and the mouse model, with a more pronounced increase in IFN-γ production by CD8^+^ T cells. Therefore, understanding the contribution of IFN-γ signaling to the activation and expansion of inflammatory macrophages and the role of these cells in CIP pathogenesis is of significant importance.

The IFN-γ–CXCL9/10–CXCR3 signaling axis, designated the ICC axis, plays a central role in recruiting autoimmune CXCR3^+^CD8^+^ T cells, which are crucial for disease development [64]. Our results confirmed a significant increase in the proportion of CXCR3^+^CD4^+^ T cells and CXCR3^+^CD8^+^ T cells in both CIP^+^ patients and the mouse model. These CD4^+^ and CD8^+^ T cells exhibited increased expression of the cytotoxic markers GZMB and PRF1, potentially contributing to lung tissue damage. Similarly, elevated CXCR3 expression, notably on CD8^+^ memory T cells, is a feature of T cells in patients with ICI-associated myocarditis [65]. Importantly, these CXCR3^+^CD8^+^ T cells express high levels of GZMB and PRF1 [66]. In vitro coculture experiments using cardiomyocytes and CXCR3^+^CD8^+^ T cells demonstrated that CXCR3^+^CD8^+^ T cells are sufficient to induce cardiomyocyte apoptosis [67]. Furthermore, we confirmed through in vitro Transwell assays that CXCL9/10 are essential for the migration of CXCR3^+^CD8^+^ T cells toward macrophages. Finally, by blocking the interaction between CXCR3^+^CD8^+^ T cells and CXCL9/10^+^ macrophages, we successfully prevented and alleviated CIP in the mouse model, indicating that this axis represents a promising therapeutic target. Although a prior study profiling cytokines in irAEs confirmed significantly elevated circulating levels of CXCL9 and CXCL10 at 2 and 6 weeks after anti-PD-1/PD-L1 therapy [68], their cellular source remained undefined. Our results demonstrated abundant expression of CXCL9 and CXCL10 specifically within the CCR2^+^ MoMΦ presented in CIP^+^ patients and the mouse model. In silico analysis further suggested that IFN-γ stimulation drove the differentiation of infiltrating monocytes or MoMΦ into CXCL9/10^+^ macrophages. Consistent with this prediction, IFN-γ stimulation of BMDMs robustly up-regulated Cxcl9 and Cxcl10 expression in vitro, whereas pharmacologic inhibition with ruxolitinib or *Stat1* knockdown markedly attenuated this induction, supporting a JAK–STAT1-dependent mechanism underlying IFN-γ-driven CXCL9/10 polarization.

When considering therapeutic strategies for CIP, it is crucial to recognize that the selected therapeutic target should ideally alleviate the inflammatory response without compromising ongoing or established tumor control. In this study, we explored 2 potential therapeutic strategies: limiting the migration of inflammatory monocytes into the lung using a dual chemokine inhibitor targeting CCR2/CCR5 (CVC) and reducing T cell recruitment using a chemokine inhibitor targeting CXCR3. Notably, accumulating literature provides evidence for the involvement of CCL2–CCR2 signaling in various inflammatory and infectious diseases [56,69–71]. Furthermore, within the tumor microenvironment, MoMΦ recruited through CCL2–CCR2 signaling constitute the predominant cluster of immunosuppressive tumor-associated macrophages, driving tumor progression and metastasis [72,73]. This suggests that targeting this pathway may offer the dual benefit of mitigating inflammation while simultaneously controlling tumors. Several selective CCR2 and CCR2/CCR5 small-molecule antagonists have entered phase I/II clinical trials for treating cancer and inflammatory diseases (NCT01413022, NCT02996110, NCT04123379, NCT03767582, NCT03496662, NCT00689273). In tumor cells, the role of CXCR3 has been reported to depend on the balance between its isoforms, CXCR3-A and CXCR3-B. The CXCR3-A isoform has been shown to mediate tumor-promoting activities, including stimulation of neovascularization and tumor growth. By contrast, CXCR3-B has been reported to exert tumor-suppressive effects through inhibition of angiogenesis and induction of malignant cell apoptosis [74]. In vitro studies confirmed that the CXCR3 antagonist AMG487 primarily suppresses the pro-tumorigenic proliferative and migratory functions of CXCR3 by blocking the CXCR3-A pathway [75]. Preclinical studies demonstrated that AMG487 exerts antitumor effects, inhibiting glioblastoma growth and suppressing metastatic spread in breast, colon, and osteosarcoma cancers, as well as melanoma [76–79]. Importantly, given that CXCR3 plays critical roles in the migration of CD4^+^ Th1 cells and CD8^+^ cytotoxic T lymphocytes in immune responses [80,81], its pathological functions have been established in autoimmune diseases (vitiligo and rheumatoid arthritis) and ICI-associated myocarditis [82–85]. A recent preclinical study demonstrated evidence for effective alleviation of the myocarditis phenotype using an anti-CXCR3 monoclonal antibody [84]. Furthermore, AMG487 has shown evidence of efficacy in multiple preclinical studies for rheumatoid arthritis [86–88].

In addition to the well-characterized CXCL9/10^+^ macrophages with IFN-γ-associated pro-inflammatory functions, we identified an expanded, phenotypically distinct PLTP^+^ macrophage cluster in CIP^+^ patients. This population exhibited a transcriptional program enriched for genes involved in lipid metabolism, inflammatory activation, and matrix remodeling (e.g. *PLTP*, *APOC1*, *APOE*, *FABP4*, *IL1B*, *SPP1*, and *MMP9*). This profile is consistent with a lipid-associated macrophage-like state described in metabolic and fibrotic disease settings [89]. Notably, SPP1^+^/MMP^high^ MoMΦ have recently been described as core effector populations driving fibrotic matrix remodeling across multiple organs [90]. Further functional studies will be required to determine whether, and in what contexts, this cluster directly contributes to CIP pathogenesis.

Our study has several limitations. While our findings suggest the significance of the CCR2^+^ MoMΦ population in CIP pathology, we did not conduct an in-depth investigation into the functional roles or specific transcriptional regulatory mechanisms governing this population. Additionally, although we observed CCR2^+^ MoMΦ partially repopulating the niche vacated by depleted resident macrophage populations in both CIP^+^ patients and the mouse model, the precise molecular mechanisms underlying the loss of resident macrophages induced by ICIs require further investigation. The functional contribution of resident macrophage depletion to ICI-associated pneumonitis also represents an important area for substantial future research. Furthermore, while we explored the prophylactic use of CCR2/CCR5 and CXCR3 small-molecule inhibitors in the CIP mouse model and obtained initial evidence of efficacy in attenuating inflammation, we did not assess the impact of these antagonists on the tumor microenvironment. The effects and safety profile of combination therapy with these antagonists on the CIP phenotype also remain to be investigated and will be addressed in future studies.

## Conclusions

In summary, our study establishes CCR2^+^ MoMΦ as pivotal effector cells in CIP through an integrated chain of evidence derived from clinical cohorts, animal models, and pharmacodynamic intervention studies. We reveal the molecular mechanism underlying a self-amplifying loop between these macrophages and CD8^+^ T cells via the CXCL9/10–CXCR3–IFN-γ axis, challenging the prevailing view of unidirectional T cell activity. Importantly, successful intervention using CCR2/CCR5 and CXCR3 small-molecule inhibitors provides proof-of-concept evidence for clinically targeting monocyte–macrophage migration and the ICC axis, demonstrating significant translational potential.

## Ethical Approval

This study was conducted in accordance with the Declaration of Helsinki. Ethical approval was granted by the Ethics Committee of the Chinese People’s Liberation Army General Hospital (approval no. S2021-553-01). Written informed consent was obtained from all participants prior to sample collection and subsequent analysis.

## Data Availability

The mouse lung scRNA-seq data generated in this study have been deposited in GSA-Human under accession number OMIX013518. All other data supporting the findings of this study are available within the article and its Supplementary Materials. Any additional information or custom scripts required to reanalyze the data reported in this paper are available upon reasonable request.
