# Supplementary material for: Recruited Monocyte-Derived Macrophages Drive T Cell Inflammation in Immune Checkpoint Inhibitor-Mediated Pneumonitis
Source: Cancer Commun (Lond). 2026 Sep 2;46:0049. doi: 10.34133/cancomm.0049 (PMC13534810; doi:10.34133/cancomm.0049)
Supplement: Supplementary file 1 [file cancomm.0049.f1.zip › 25-00336 Supplementary file - final.pdf]

Recruited monocyte-derived macrophages drive T cell inflammation in immune checkpoint inhibitor-mediated pneumonitis

Xiaoran Cui[1,2†]; Renyong Zhi[3†]; Xiaoyan Li[1†]; Yinming Ji[1,4†]; Zhisong Hu[1]; Pengfei Cui[5]; Yuhui Qin[6]; Tao Li[7]; Liangliang Wu[2,8]; Lingxiong Wang[2,8]; Jinfeng Li[2]; Yibing Bai[7]; Hui Li[1]; Tianyi Liu[2,8]*; Yi Hu[1,2,7]*

1, Senior Department of Oncology, the Fifth Medical Center of Chinese PLA General Hospital, Beijing, P. R. China.

2, Institute of Oncology, the Fifth Medical Center of Chinese PLA General Hospital, Beijing, P. R. China.

3, Department of Hematology, Beijing Friendship Hospital, Capital Medical University, Beijing, P. R. China.

4, Department of Oncology, the Second Affiliated Hospital of Anhui Medical University, Hefei, Anhui, P. R. China.

5, Department of Oncology, the Second Medical Center of Chinese PLA General Hospital, Beijing, P. R. China.

6, Department of Gastroenterology, Beijing Friendship Hospital, Capital Medical University, Beijing, P. R. China.

7, Department of Oncology, the First Medical Center of Chinese PLA General Hospital, Beijing, P. R. China.

8, Institute of Oncology, the First Medical Center of Chinese PLA General Hospital, Beijing, P. R. China.

†: These authors contributed equally: Xiaoran Cui, Renyong Zhi, Xiaoyan Li, Yinming Ji

*: Corresponding author: Yi Hu huyi301zlxb@sina.com; Tianyi Liu tianyiliu08@hotmail.com

Supplementary Figures

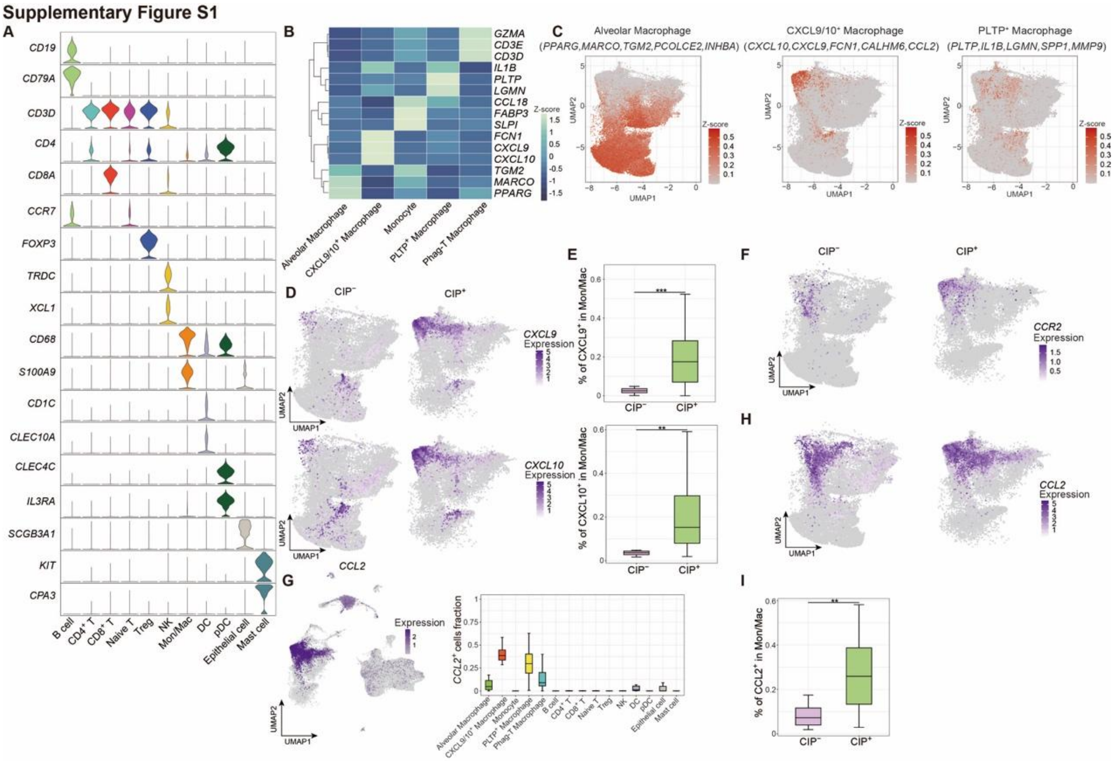

Figure S1: scRNA-seq reveals alterations in immune cell composition within BALF from CIP$^+$ and CIP$^-$ patients. (A) Violin plots showing expression of canonical marker genes in each major cell type. **(B)** Heatmap showing representative marker genes for each monocyte/macrophage cluster identified from the integrated BALF scRNA-seq data. **(C)** Z-score feature plots of monocytes/macrophages subcluster signatures. UMAP embedding of monocytes/macrophages with overlaid Z-score signature intensity for marker genes enriched in each indicated monocytes/macrophages state: alveolar macrophages (*PPARG*, *MARCO*, *TGM2*, *PCOLCE2*, *INHBA*), CXCL9/10$^+$ macrophages (*CXCL10*, *CXCL9*, *FCN1*, *CALHM6*, *CCL2*), and PLTP$^+$ macrophages (*PLTP*, *IL1B*, *LGMN*, *SPP1*, *MMP9*). **(D)** UMAP plot showing *CXCL9* and *CXCL10* expression in BALF monocytes/macrophages. **(E)** Boxplot representing the percentage of CXCL9$^+$ and CXCL10$^+$ cells within BALF monocytes/macrophages. Two-tailed Mann-Whitney test. **(F)** UMAP plot showing *CCR2* expression in BALF monocytes/macrophages. **(G)** *CCL2* expression across major lung cell populations. Left, UMAP feature plot showing the distribution of *CCL2* expression. Right, boxplots summarizing the fraction of CCL2$^+$ cells in each

annotated cell type. **(H)** UMAP plot showing *CCL2* expression in BALF monocytes/macrophages. **(I)** Boxplot representing the percentage of $CCL2^+$ cells within BALF monocytes/macrophages. Two-tailed Mann-Whitney test; significance denoted as $^{**}P < 0.01$, $^{***}P < 0.001$.

Abbreviations: BALF, bronchoalveolar lavage fluid; CALHM6, calcium homeostasis modulator family member 6; CCL2, C-C motif chemokine ligand 2; CCR2, C-C motif chemokine receptor 2; CIP, checkpoint inhibitor pneumonitis; CXCL9, C-X-C motif chemokine ligand 9; CXCL10, C-X-C motif chemokine ligand 10; DC, dendritic cell; FCN1, ficolin 1; IL1B, interleukin 1 beta; INHBA, inhibin subunit beta A; LGMN, legumain; MARCO, macrophage receptor with collagenous structure; MMP9, matrix metallopeptidase 9; Mon/Mac, monocytes/macrophages; PCOLCE2, procollagen C-endopeptidase enhancer 2; pDC, plasmacytoid dendritic cell; Phag-T macrophage, T-cell-phagocytosing macrophage; PLTP, phospholipid transfer protein; PPARG, peroxisome proliferator-activated receptor gamma; scRNA-seq, single-cell RNA sequencing; SPP1, secreted phosphoprotein 1; TGM2, transglutaminase 2; Treg, regulatory T cell; UMAP, uniform manifold approximation and projection.

Supplementary Figure S2

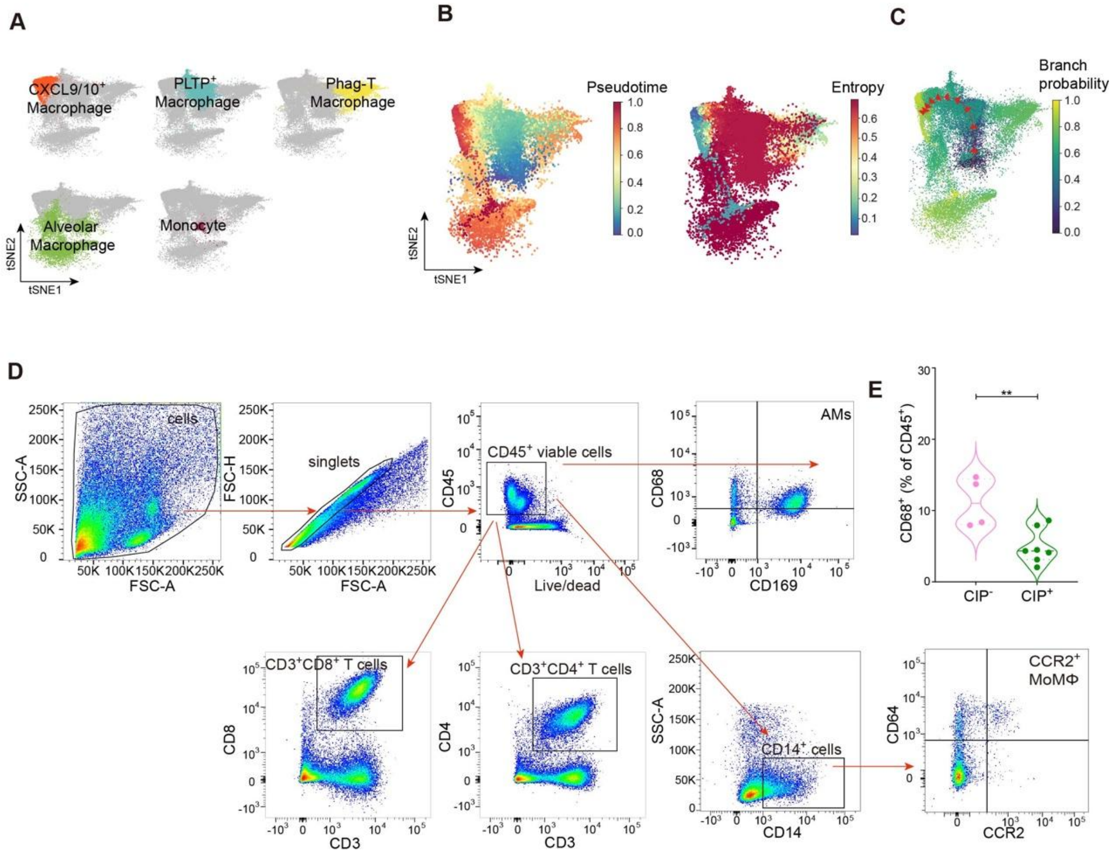

Figure S2: Palantir analysis of monocytes/macrophages in BALF from CIP$^+$ patients and flow cytometry gating strategy. (A) t-SNE visualization of BALF monocytes/macrophages, colored by annotated cell clusters. **(B)** Pseudotime and entropy analysis of BALF monocytes/macrophages. CXCL9/10$^+$ macrophages exhibited high pseudotime and low entropy values, indicative of a differentiated cell state. **(C)** Trajectory analysis identified CXCL9/10$^+$ macrophages as the terminal differentiation state of monocytes. **(D)** Flow cytometry gating strategy for BALF CD3$^+$CD4$^+$ T cells, CD3$^+$CD8$^+$ T cells, alveolar macrophages (AMs), and CCR2$^+$ monocyte-derived macrophages (CCR2$^+$ MoMΦ). **(E)** Quantification of CD68$^+$ cells in BALF from 7 CIP$^+$ and 4 CIP$^-$ patients in the independent validation cohort at the Chinese PLA General Hospital, measured by flow cytometry. Data were expressed as percentages of total CD45$^+$ cells (Mann-Whitney U test; significance denoted as $**P < 0.01$; $n = 4/7$ per group).

Abbreviation: MoMΦ, monocyte-derived macrophages; t-SNE, t-distributed stochastic neighbor embedding.

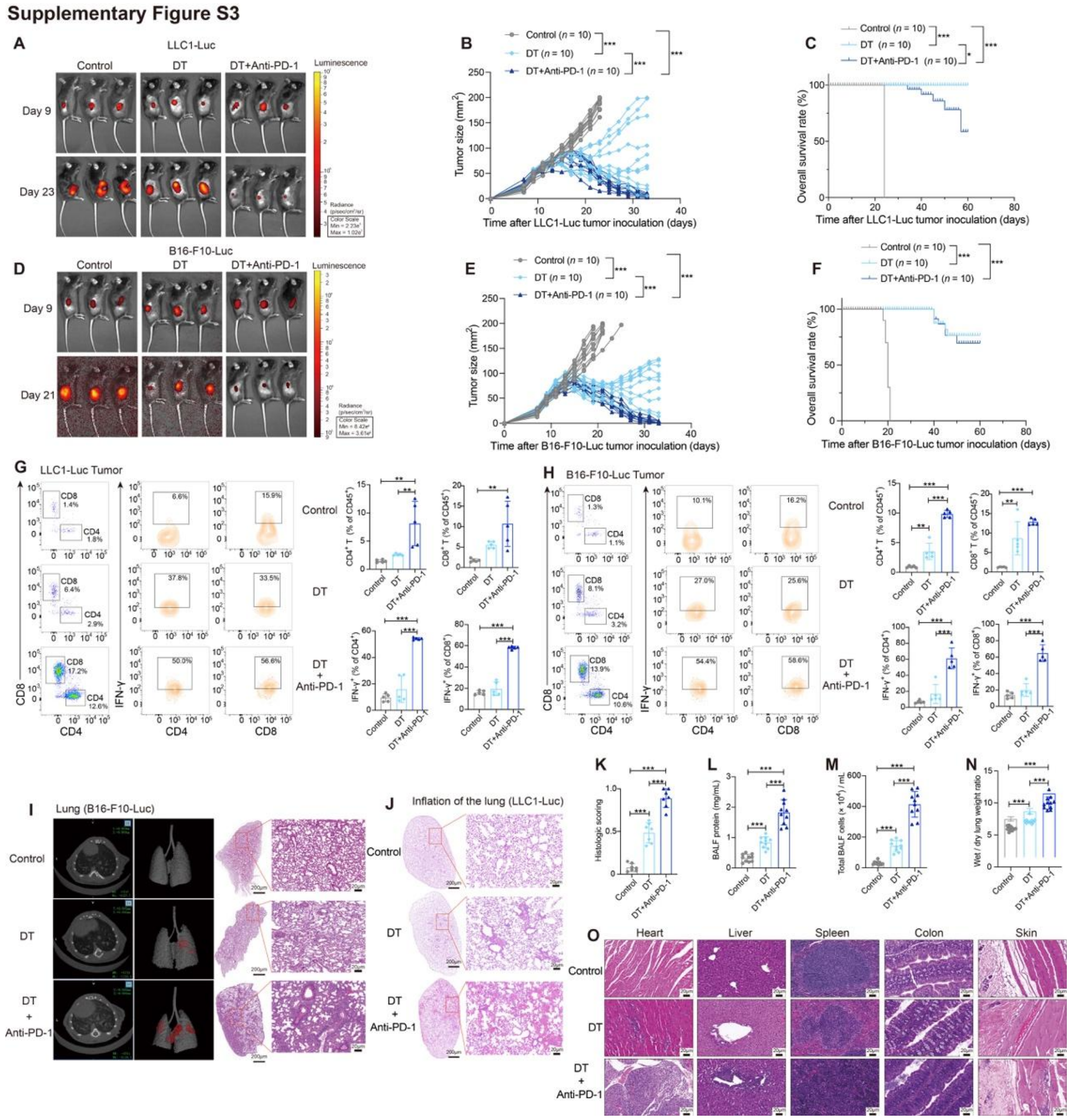

Figure S3: Treg depletion plus anti-PD-1 therapy suppresses tumor growth but triggers pneumonitis and multi-organ inflammation in *Foxp3-DTR-GFP* mice.

(A) In vivo imaging of LLC1-Luc tumors in *Foxp3-DTR-GFP* mice at Day 9 and Day 23 (when control group tumors reached the humane endpoint) following subcutaneous cell injection. **(B)** In vivo growth curve of LLC1-Luc tumors. Tumor growth was measured using a caliper. The day of subcutaneous tumor cell injection in mice was designated as Day 0. Each curve showing tumor size in one individual mouse measured longitudinally. Data were pooled from 2 independent experiments (n = 10 per group). Two-way ANOVA with Dunnett's multiple comparisons test was used; significance denoted as ***P < 0.001. **(C)** Kaplan-Meier survival curves of mice after subcutaneous LLC1-Luc tumor inoculation among treatment groups. Data were pooled from 2 independent experiments (n = 10 per group). Log-rank test was used;

significance denoted as *P < 0.05, ***P < 0.001. **(D)** In vivo imaging of B16-F10-Luc tumors in *Foxp3-DTR-GFP* mice at Day 9 and Day 21 following subcutaneous cell injection. **(E)** In vivo growth curve of B16-F10-Luc tumors. Data were pooled from 2 independent experiments (n = 10 per group). Two-way ANOVA with Dunnett's multiple comparisons test was used; significance denoted as ***P < 0.001. **(F)** Kaplan-Meier survival curves of mice after subcutaneous B16-F10-Luc tumor inoculation among treatment groups. Data were pooled from 2 independent experiments (n = 10 per group). Log-rank test was used; significance denoted as ***P < 0.001. **(G-H)** Flow cytometric analysis of T-cell responses in two tumor models. Representative flow cytometry plots (left) and quantification (right) of tumor-infiltrating T cells from LLC1-Luc **(G)** and B16-F10-Luc **(H)** tumors in Control, DT, and DT + anti-PD-1 groups (n = 5 per group). Shown were the frequencies of $CD4^+$ and $CD8^+$ T cells among $CD45^+$ cells, and IFN-γ^+CD4^+ and IFN-γ^+CD8^+ T cells among their respective subsets. One-way ANOVA with Tukey's multiple comparisons test was used; significance denoted as **P < 0.01, ***P < 0.001. **(I)** Representative micro-CT images (left), 3D-reconstructed lung tissue (gray) and inflammatory foci (red) (middle), and H&E-stained lung sections (right) from B16-F10-Luc-bearing mice across treatment groups. Magnification: ×4 and ×40. Scale bars: 200 μm and 20 μm. **(J-N)** Assessment of lung injury in the LLC1-Luc model. **(J)** Representative H&E-stained sections of inflated lungs from LLC1-Luc model. Boxed regions in the whole-lung images were shown at higher magnification to highlight parenchymal inflammation and tissue injury. Magnification: ×4 and ×40. Scale bars: 200 μm and 20 μm. **(K)** Semi-quantitative histologic scoring of lung pathology. **(L)** BALF protein concentration. **(M)** Total BALF cell counts. **(N)** Wet-to-dry lung weight ratio. Data were pooled from 2 independent experiments (n = 7-10 per group). One-way ANOVA with Tukey's multiple comparisons test was used; significance denoted as ***P < 0.001. **(O)** Representative H&E-stained sections of multiple tissues from LLC1-Luc tumor-bearing mice across treatment groups.

Abbreviation: B16-F10-Luc, luciferase-expressing B16-F10 melanoma cells; DT, diphtheria toxin; DTR, diphtheria toxin receptor; Foxp3, forkhead box P3; Foxp3-DTR-GFP mice, forkhead box P3–diphtheria toxin receptor–green fluorescent protein transgenic mice; H&E, hematoxylin and eosin; IFN-γ, interferon-gamma; LLC1-Luc, luciferase-expressing Lewis lung carcinoma 1 cells; micro-CT, micro-computed tomography; PD-1, programmed cell death protein 1.

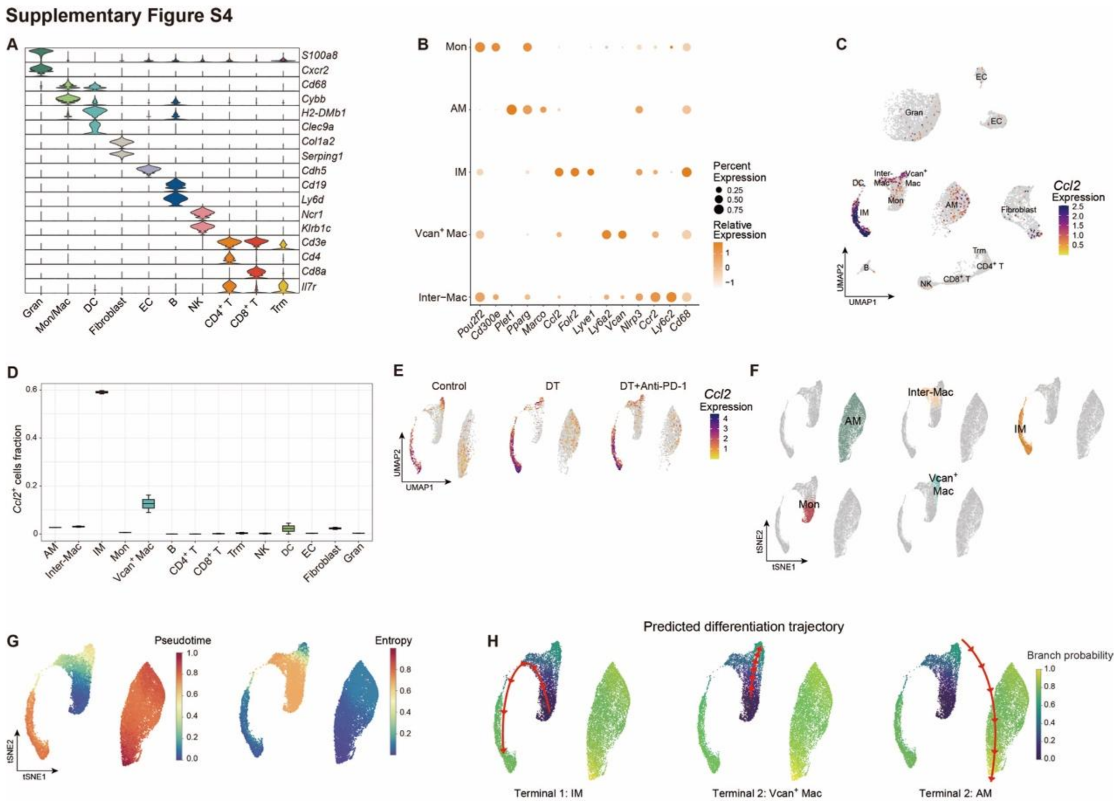

Figure S4: ScRNA-seq analysis of lungs from *Foxp3-DTR-GFP* mice. (A) Violin plots showing expression of identified specific gene markers across major cell types in the integrated lung scRNA-seq data. **(B)** Dot plots showing differentially expressed genes in each monocyte/macrophage cluster. **(C)** UMAP feature plot showing the distribution of *Ccl2* expression across mouse lung cell populations. **(D)** Boxplots summarizing the fraction of *Ccl2*$^{+}$ cells in each annotated cell type. **(E)** UMAP plot showing *Ccl2* expression in monocytes/macrophages of lungs from *Foxp3-DTR-GFP* mice. **(F-H)** Palantir analysis of monocytes/macrophages in lungs from *Foxp3-DTR-GFP* mice. **(F)** t-SNE visualization of mouse lung monocyte/macrophage, colored by annotated cell clusters. **(G)** Pseudotime and entropy analysis of mouse lung monocyte/macrophage revealed distinct differentiation states. IM and AM showed high pseudotime values coupled with low entropy, suggesting terminally differentiated states. In contrast, Inter-Mac displayed moderate pseudotime and entropy values, indicating a transitional state. **(H)** Trajectory analysis revealed that IM, Vcan^{+} Mac, and AM represent three terminal differentiation states of monocytes. Monocytes first differentiate into Inter-Mac, then acquire IM phenotypes.

Abbreviation: AM, alveolar macrophages; B, B cells; DC, dendritic cells; EC, endothelial cells; Gran, granulocytes; IM, interstitial macrophages; Inter-Mac, intermediate-stage inflammatory macrophages; Mon, monocytes; NK, natural killer cells; Trm, tissue-resident memory T cells; Vcan^{+} Mac, versican-positive macrophages.

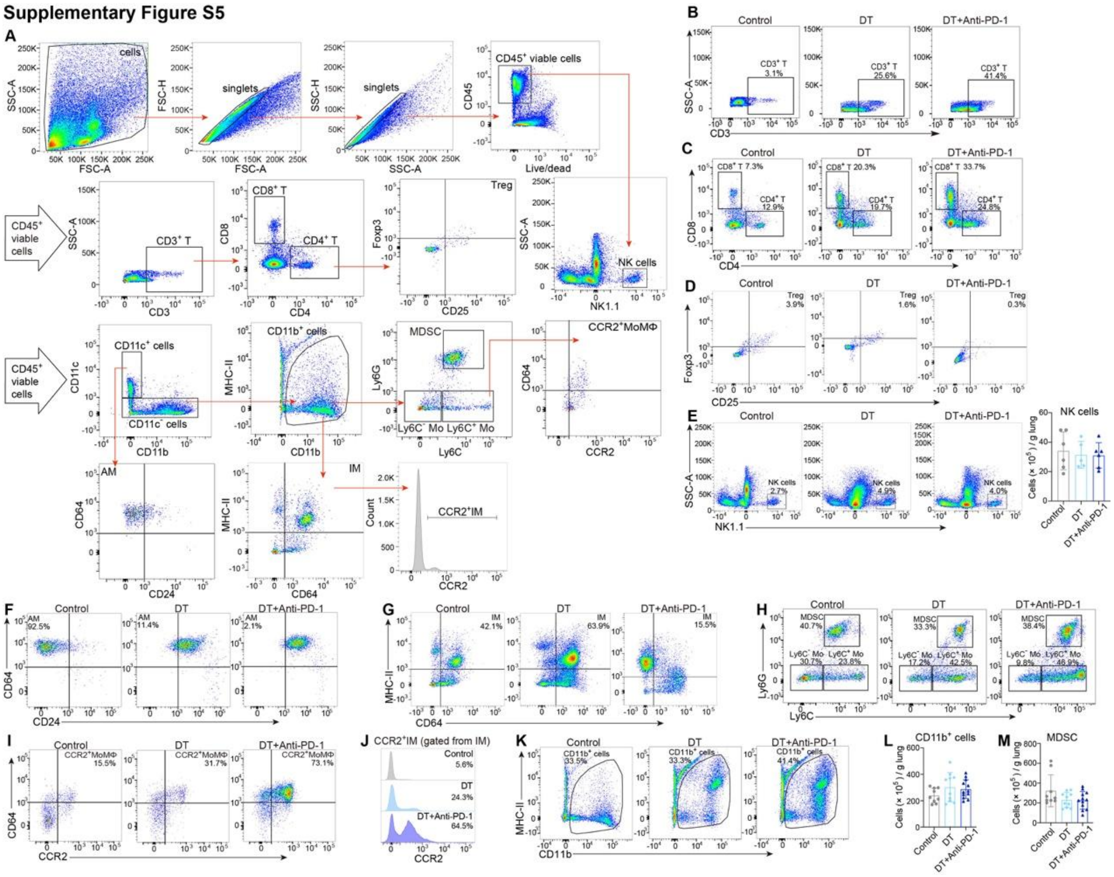

Figure S5: Flow cytometric analysis of lymphoid and myeloid cells of lungs across treatment groups in mice. (A) The gating strategy for murine lung immune cell subsets was adapted from Misharin et al. [41] and Öz et al. [42]. Cells isolated from enzymatically digested mouse lungs were identified as immune cells through CD45 staining after excluding doublets and dead cells. First, lymphoid populations expressing specific markers were identified: NK cells (CD45^{+}NK1.1^{+}), CD3^{+} T cells (CD45^{+}CD3^{+}), CD4^{+} T cells (CD45^{+}CD3^{+}CD4^{+}), CD8^{+} T cells (CD45^{+}CD3^{+}CD8^{+}), and Treg cells (CD4^{+}CD25^{+}Foxp3^{+}). Second, myeloid populations expressing specific markers were identified: alveolar macrophages (AMs; CD45^{+}CD11c^{+}CD11b^{-}CD64^{+}CD24^{-}), interstitial macrophages (IMs;

CD45^{+}CD11c^{-}CD11b^{+}MHC II^{+}CD64^{+}), Ly6C^{-} monocytes (Ly6C^{-} Mo; CD45^{+}CD11c^{-}CD11b^{+}Ly6G^{-}Ly6C^{-}), Ly6C^{+} monocytes (Ly6C^{+} Mo; CD45^{+}CD11c^{-}CD11b^{+}Ly6G^{-}Ly6C^{+}), CCR2^{+} MoMΦ (CD45^{+}CD11c^{-}CD11b^{+}Ly6G^{-}Ly6C^{+}CD64^{+}CCR2^{+}; gated from Ly6C^{+} Mo), CCR2^{+} IMs (CD45^{+}CD11c^{-}CD11b^{+}MHC II^{+}CD64^{+}CCR2^{+}; gated from IMs), CD11b^{+} cells (CD45^{+}CD11c^{-}CD11b^{+}), and MDSCs (CD45^{+}CD11c^{-}CD11b^{+}Ly6G^{+}Ly6C^{+}). **(B-M)** Representative profiling and quantitative analyses of the aforementioned lymphoid and myeloid subsets across treatment groups: CD3^{+} T cells **(B)**, CD4^{+} and CD8^{+} T cells **(C)**, Treg cells **(D)**, NK cells **(E)**, AMs **(F)**, IMs **(G)**, Ly6C^{-}/Ly6C^{+} Mo **(H)**, CCR2^{+} MoMΦ **(I)**, CCR2^{+} IMs **(J)**, CD11b^{+} cells **(K)**, and MDSCs **(L, M)**. Quantitative analyses corresponding to the main results are shown in **Figure 2I, J**. **Abbreviations:** MDSCs, myeloid-derived suppressor cells; MHC-II, major histocompatibility complex class II; Ly6C, lymphocyte antigen 6 complex, locus C; Ly6G, lymphocyte antigen 6 complex, locus G.

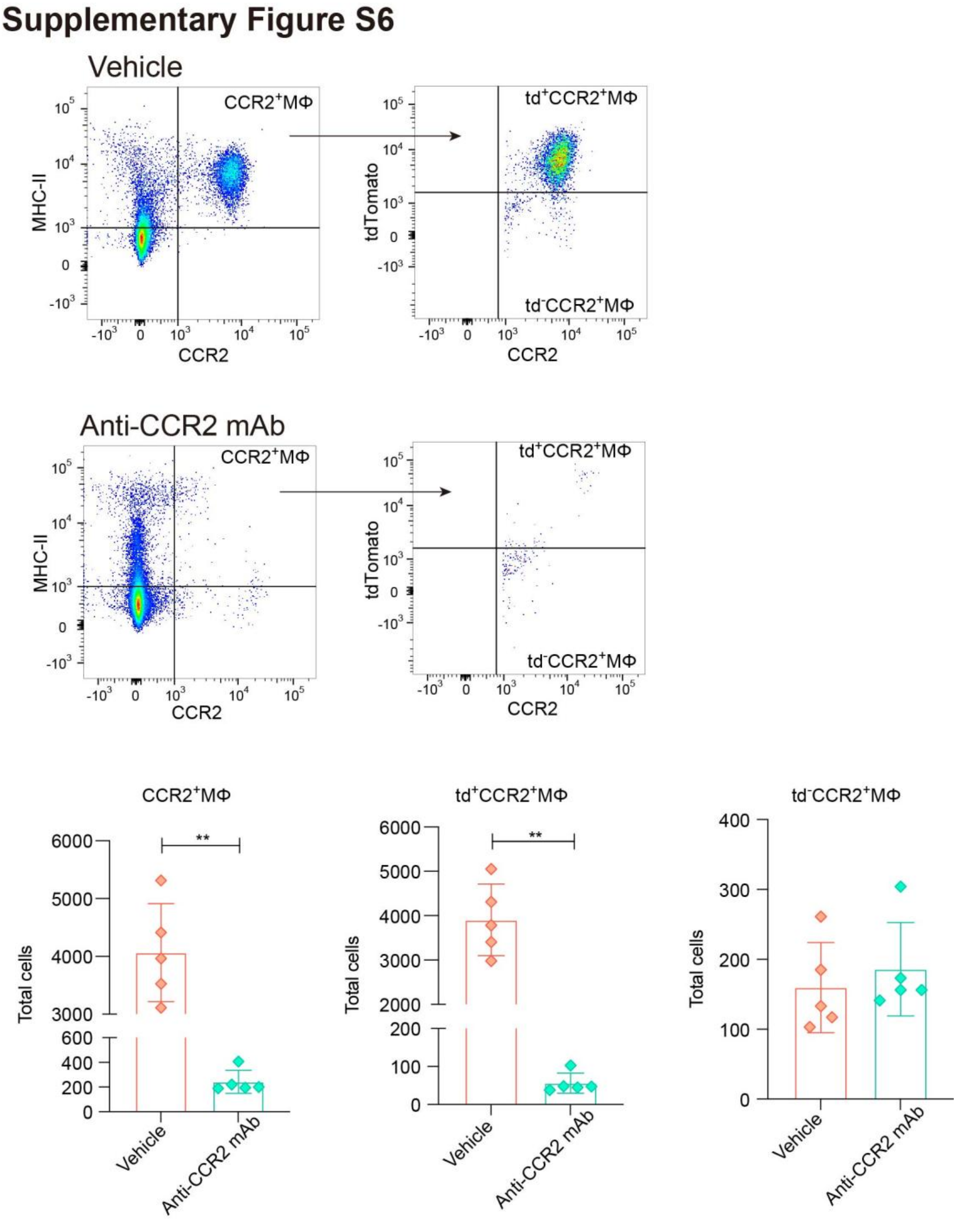

Figure S6: Lineage tracing experiments demonstrated that anti-CCR2 antibody reduces CCR2$^+$ macrophage numbers by suppressing the recruitment of CCR2$^+$ monocytes, rather than through direct action on CCR2$^+$ macrophages. To investigate the origin of CCR2$^+$ macrophages in the lungs and confirm their derivation from peripheral blood monocytes, *Ccr2^{ertcre}Rosa^{tdTomato}* mice were employed to label and track peripheral monocytes. Tamoxifen (60 mg/kg) was administered intraperitoneally every 3 days for 1 week. Subsequently, anti-CCR2 antibody or

control IgG was administered by intraperitoneal injection. Flow cytometric analysis of lung tissues defined $tdTomato^{+}CCR2^{+}$ macrophages ($td^{+}CCR2^{+}M\Phi$) as recently recruited $CCR2^{+}$ monocyte-derived macrophages ($CCR2^{+}$ MoMΦ), while $tdTomato^{-}CCR2^{+}$ macrophages ($td^{-}CCR2^{+}M\Phi$) represented tissue-resident $CCR2^{+}$ macrophages. Quantification of total cell numbers for $CCR2^{+}$ macrophages ($CCR2^{+}M\Phi$), $td^{+}CCR2^{+}M\Phi$, and $td^{-}CCR2^{+}M\Phi$ within $CD45^{+}CD68^{+}$ cells was calculated by multiplying the percentage of live cells by total cell counts. Two-tailed Mann-Whitney U test ($n = 5$ per group), with significance levels denoted as $^{**}P < 0.01$.

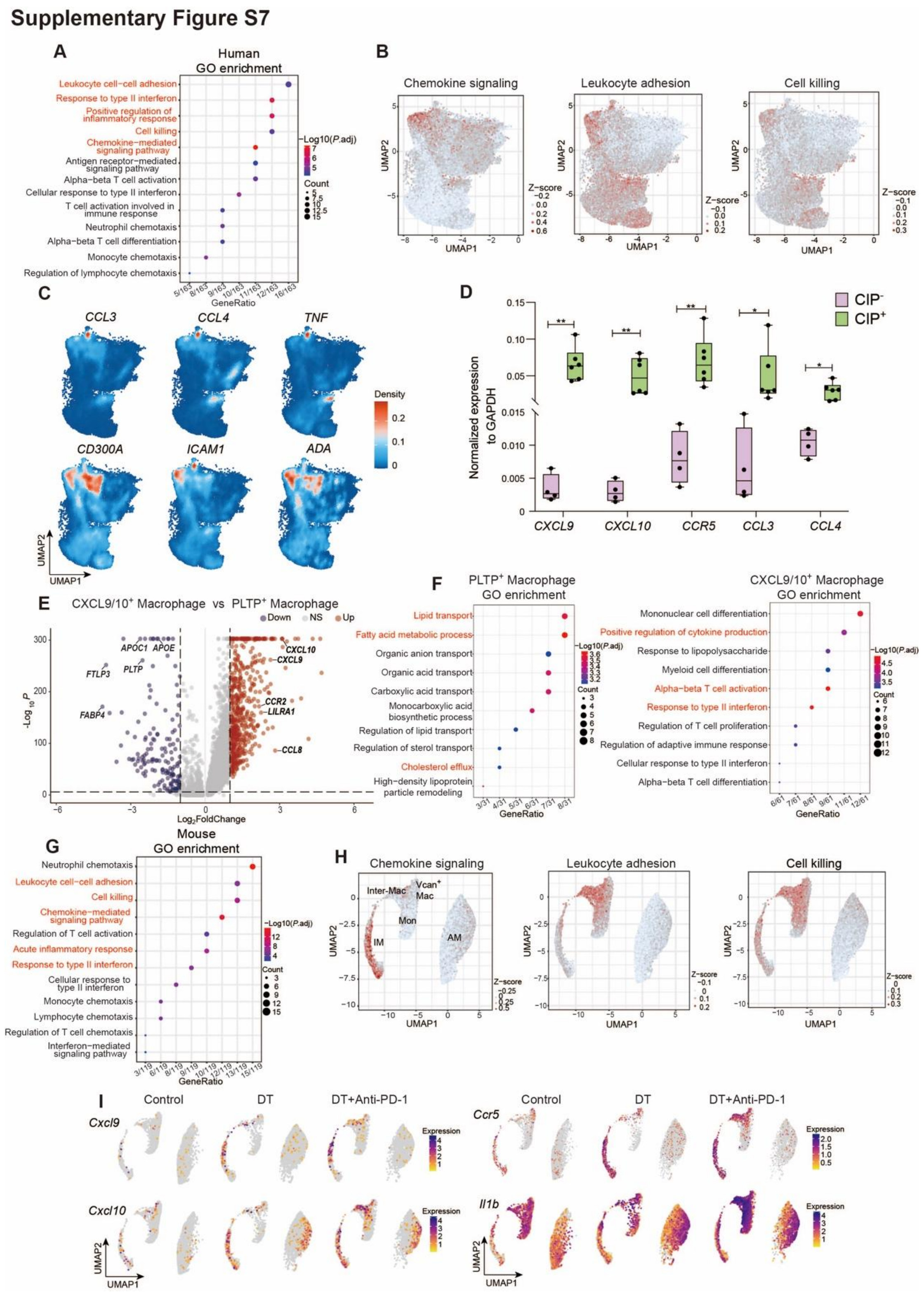

Figure S7: Transcriptional signature of CCR2^{+}CXCL9/10^{+} MoMΦ in CIP^{+} patients and the mouse model. (A) Gene Ontology (GO) enrichment analysis of upregulated genes in CIP^{+} monocytes/macrophages was performed. Genes were selected based on Seurat differential expression analysis with an adjusted *P* value <

0.05 and $|log_2FC| > 1$. The top enriched biological processes (BP), ranked by *P* value, were shown. **(B)** Single-cell feature plots of Z-score normalized gene expression highlighting chemokine signaling, leukocyte adhesion, and cell killing pathways enriched in BALF monocytes/macrophages from patients. **(C)** Density plot of specific genes showing in **Figure 3C**. **(D)** RT-qPCR analysis showed significantly increased mRNA expression of *CXCL9*, *CXCL10*, *CCR5*, *CCL3*, and *CCL4* in BALF from CIP$^+$ compared to CIP$^-$ (Mann-Whitney test, 2-tailed; significance denoted as $^*P < 0.05$, $^{**}P < 0.01$; $n = 4/6$ per group). **(E)** Volcano plot showing differentially expressed genes between CXCL9/10$^+$ macrophages and PLTP$^+$ macrophages identified from mouse scRNA-seq. **(F)** GO enrichment analyses of genes preferentially expressed in PLTP$^+$ macrophages (left) and CXCL9/10$^+$ macrophages (right). The top enriched BP, ranked by *P* value, are shown. **(G)** GO analysis of upregulated genes in lung monocytes/macrophages of DT + anti-PD-1-treated mice (vs. Control) was performed. Genes were selected based on Seurat differential expression analysis with an adjusted *P* value < 0.05 and $|log_2FC| > 1$. BP were grouped and sorted by adjusted *P* value. **(H)** Single-cell feature plots of Z-score normalized gene expression highlighting chemokine signaling, leukocyte adhesion, and cell killing pathways enriched in lung monocytes/macrophages from *Foxp3-DTR-GFP* mice. **(I)** UMAP plot showing the expression of *Cxcl9, Cxcl10, Ccr5,* and *Il1b* within the lung monocytes/macrophages from *Foxp3-DTR-GFP* mice.

Abbreviation: ADA, adenosine deaminase; APOC1, apolipoprotein C1; APOE, apolipoprotein E; CCL3, C-C motif chemokine ligand 3; CCL4, C-C motif chemokine ligand 4; CCL8, C-C motif chemokine ligand 8; CCR5, C-C motif chemokine receptor 5; FABP4, fatty acid-binding protein 4; FTLP3, ferritin light chain pseudogene 3; ICAM1, intercellular adhesion molecule 1; Il1b, interleukin 1 beta; LILRA1, leukocyte immunoglobulin-like receptor A1; TNF, tumor necrosis factor.

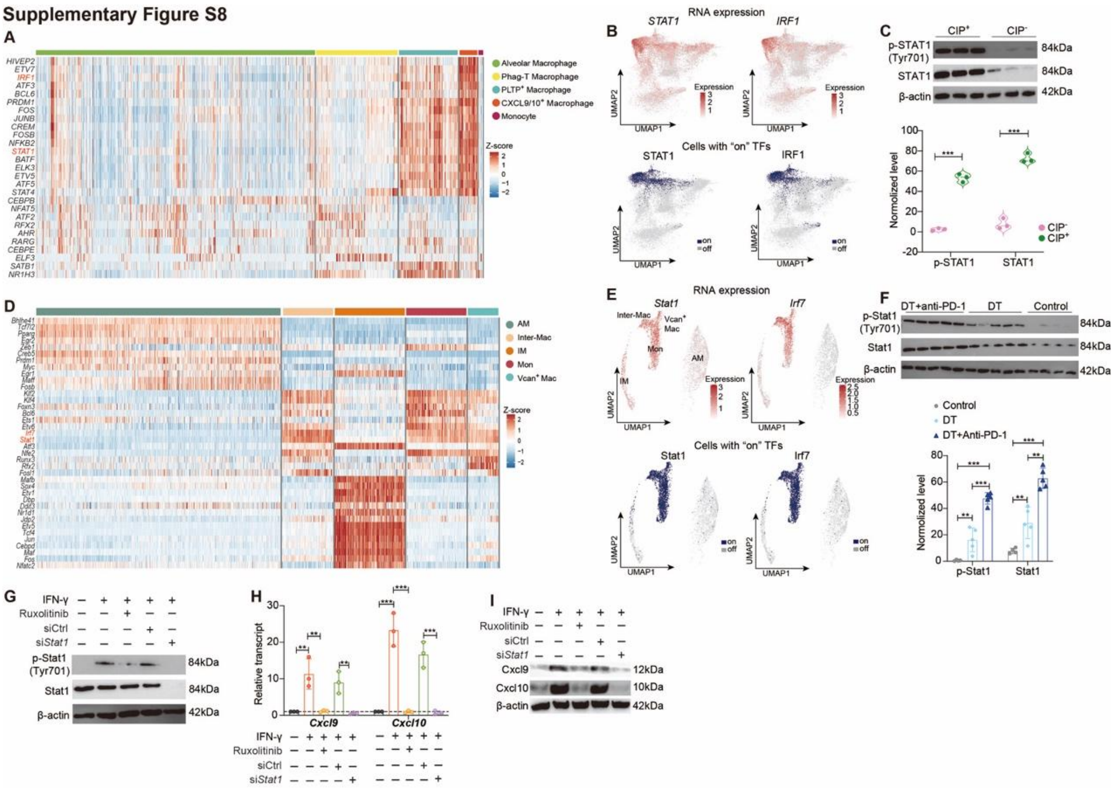

Figure S8: Activation of STAT1 mediates IFN-γ-induced expansion of CXCL9/10^{+} macrophages. (A-C) showing BALF from CIP^{+} patients. **(A)** Heatmap of transcription factor (TF) activity based on AUC scores in monocytes/macrophages, analyzed using the PySCENIC (single-cell regulatory network inference and clustering) pipeline. Each row corresponds to a TF. Top bar annotations indicate cell types defined by scRNA-seq cluster identities. Color intensity reflects the Z-scores of the AUC values. In CXCL9/10^{+} macrophages, the IFN-γ downstream transcription factors STAT1 and IRF1 exhibited specific activation. **(B)** UMAP plots showing mRNA expression levels of *STAT1* and *IRF1* (upper) and cells passing the binary threshold defined by the AUC histogram (lower). Cells passing the threshold defined by the regulon AUC distribution were labeled "on" and considered to have active regulons, whereas the remaining cells were labeled "off." **(C)** Protein levels and phosphorylation states of STAT1 in BALF from CIP^{+} and CIP^{-} patients were determined by Western blotting. Student's t-test, two-tailed; significance denoted as ***$P < 0.001$; $n = 3$ per group. **(D-F)** showing lungs from *Foxp3-DTR-GFP* mice. **(D)** Heatmap of TF activity based on AUC scores in monocytes/macrophages, analyzed using the PySCENIC pipeline. In Inter-Mac, the IFN-γ downstream transcription

factors Stat1 and Irf7 exhibited specific activation. **(E)** UMAP plots showing mRNA expression levels of *Stat1* and *Irf7* (upper) and cells passing the binary threshold defined by the AUC histogram (lower). **(F)** Protein levels and phosphorylation states of Stat1 in lung tissues from each treatment group were determined by Western blotting. One-way ANOVA with Tukey's multiple comparisons test; significance denoted as $^{**}P < 0.01$, $^{***}P < 0.001$; $n = 4$-5 per group. **(G)** Bone marrow-derived macrophages (BMDMs) from mice were treated with IFN-γ in the presence or absence of the JAK1/2 inhibitor ruxolitinib or transfected with *Stat1 siRNA* (si*Stat1*) or *control siRNA* (siCtrl). Protein levels and phosphorylation states of Stat1 were determined by Western blotting. **(H)** Relative mRNA expression of *Cxcl9* and *Cxcl10* in BMDMs under the indicated conditions, as determined by real-time qPCR. One-way ANOVA with Tukey's multiple comparisons test; significance denoted as $^{**}P < 0.01$, $^{***}P < 0.001$; $n = 3$ per group. **(I)** Protein expression of Cxcl9 and Cxcl10 in BMDMs under the indicated conditions was determined by Western blotting.

Abbreviation: AUC, area under the curve; STAT1, signal transducer and activator of transcription 1; IRF1, interferon regulatory factor 1; Irf7, interferon regulatory factor 7; JAK1/2, Janus kinase 1/2.

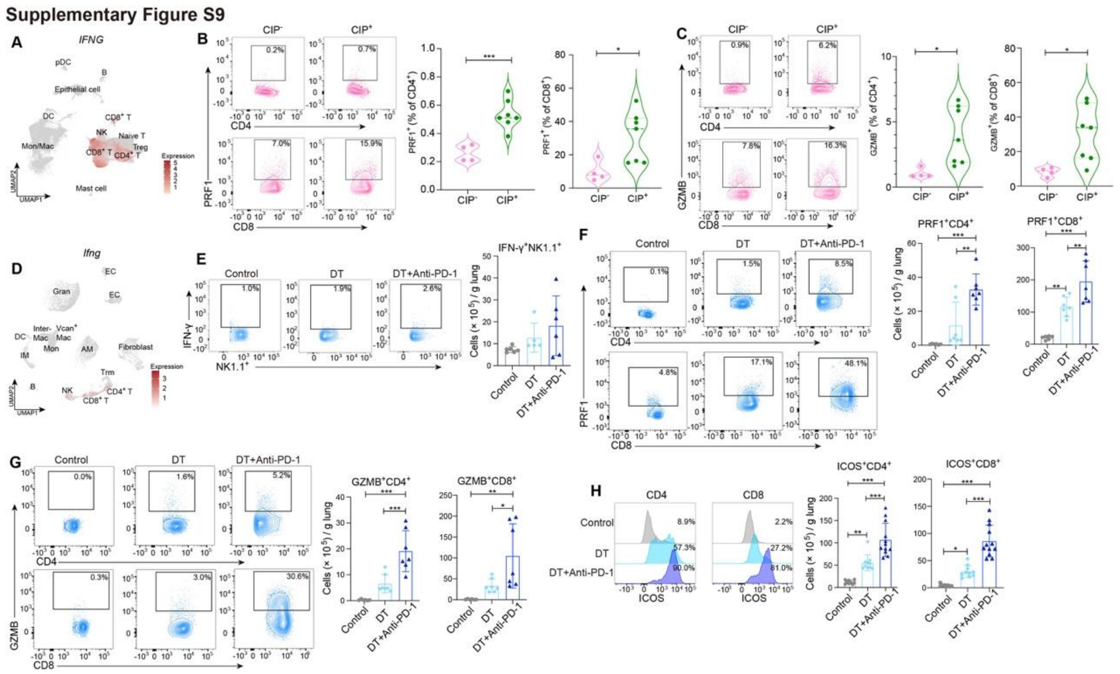

Figure S9: Expansion of T cells exhibiting elevated expression of cytotoxic effector molecules is observed in BALF from CIP^+ patients and lung tissues of *Foxp3-DTR-GFP* mice. (A) UMAP plot showing IFN-γ-specific expression localized to NK/T cell clusters among all cell types in patient BALF, with pronounced enrichment in $CD8^+$ T cells and $CD4^+$ T cell clusters. **(B)** Flow cytometry was used to assess the proportions of Perforin $(PRF1)^+CD4^+$ T and $PRF1^+CD8^+$ T cells in BALF from patients. **(C)** Flow cytometry was used to assess the proportions of Granzyme B $(GZMB)^+CD4^+$ T and $GZMB^+CD8^+$ T cells in BALF from patients. Two-tailed Mann-Whitney U test was used; significance denoted as *$P < 0.05$, ***$P < 0.001$; CIP-: $n = 4$; CIP+: $n = 7$. **(D)** UMAP plot demonstrated IFN-γ expression confined exclusively to NK/T cell clusters in the lungs of *Foxp3-DTR-GFP* mice. **(E)** Flow cytometry quantified the absolute counts of IFN-γ^+NK1.1^+ cells in murine lungs across treatment groups, with no significant intergroup differences detected (One-way ANOVA with Tukey's test; n = 5-7 per group). **(F)** Flow cytometry was used to assess the absolute counts of $PRF1^+CD4^+$ T and $PRF1^+CD8^+$ T cells in murine lungs across treatment groups. **(G)** Flow cytometry was used to assess the absolute counts of $GZMB^+CD4^+$ T and $GZMB^+CD8^+$ T cells in murine lungs across treatment groups. **(H)** Flow cytometry was used to assess the absolute counts of $ICOS^+CD4^+$ T and $ICOS^+CD8^+$ T cells in murine lungs across treatment groups. One-way ANOVA with Tukey's test was used; significance denoted as *$P < 0.05$, **$P < 0.01$, ***$P < 0.001$.

Abbreviation: ICOS, inducible T-cell co-stimulator.

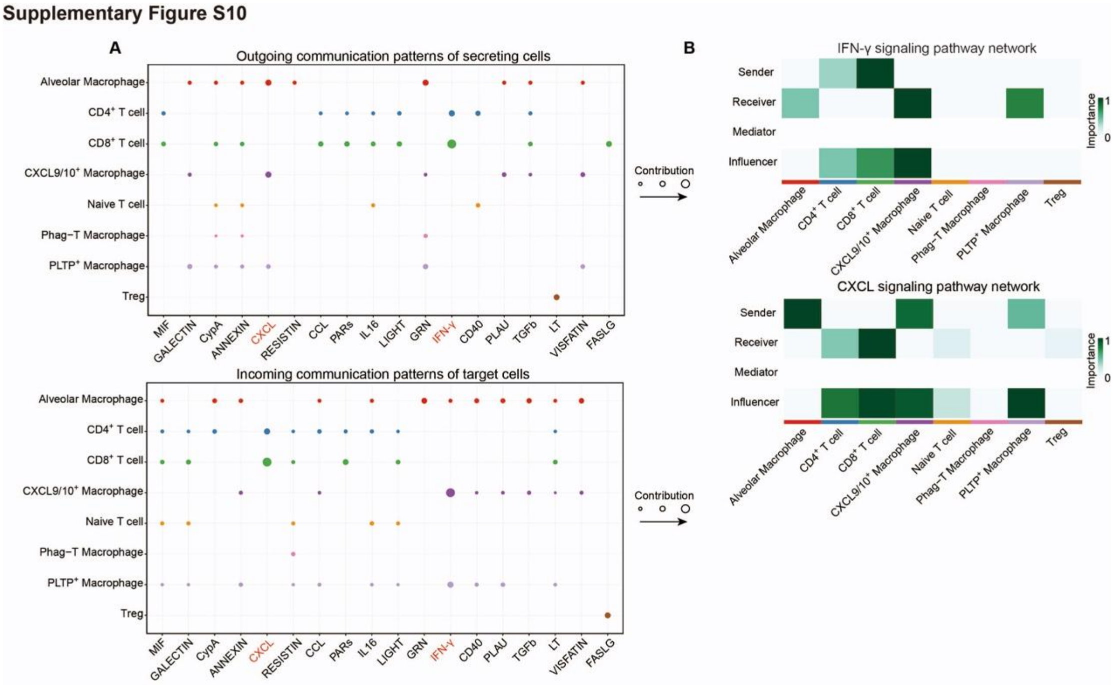

Figure S10: Computationally predicted analysis of macrophage-T cell crosstalk in patient BALF. (A) The dot plots illustrated a comparison between the outgoing signaling patterns of secretory cells and the incoming signaling patterns of target cells in patient BALF. The size of the dots corresponds to the contribution scores calculated by pattern recognition analysis, with larger dots indicating higher contribution scores and greater enrichment of signaling pathways in the corresponding cell subpopulations. **(B)** The heatmap displayed the relative importance of each cell subpopulation based on four network centrality measures calculated for the IFN-γ and CXCL signaling networks in patient BALF.

Supplementary Figure S11

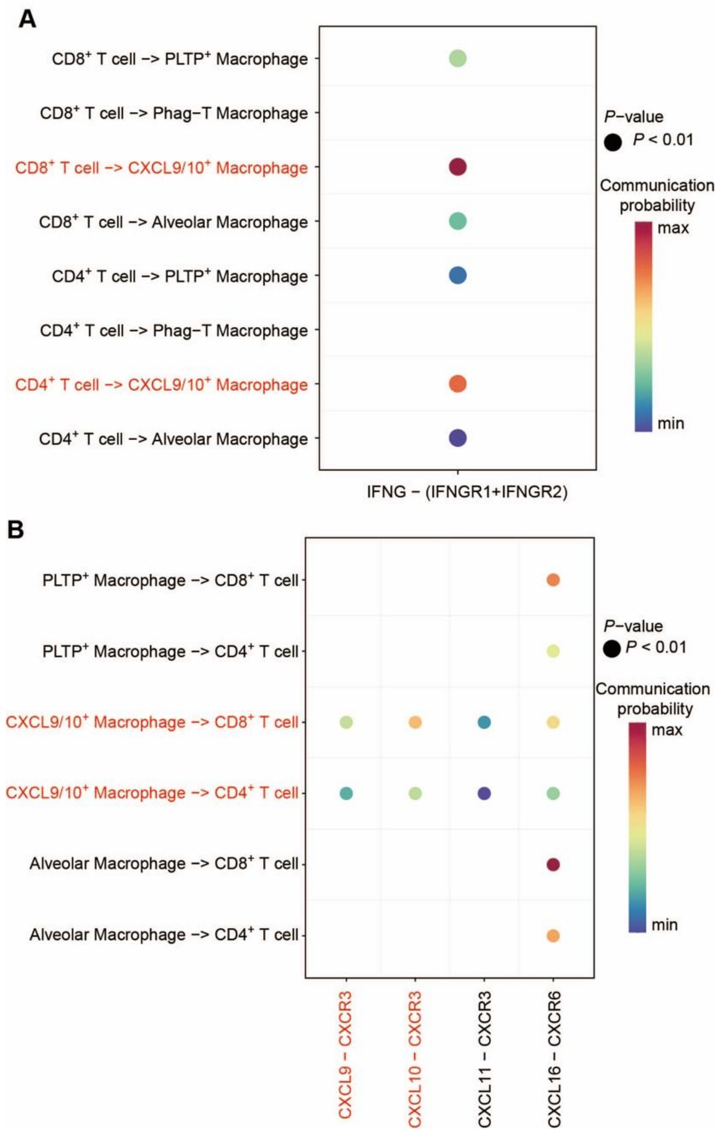

Figure S11: Interaction strength of T cells and macrophages in IFN-γ and CXCL signaling within patient BALF. (A-B) The dot plots illustrated interaction strength between T cells and macrophages within the IFN-γ **(A)** and CXCL **(B)** signaling pathways in patient BALF, with statistical significance (*P* values) computed via the R package CellChat.

Supplementary Figure S12

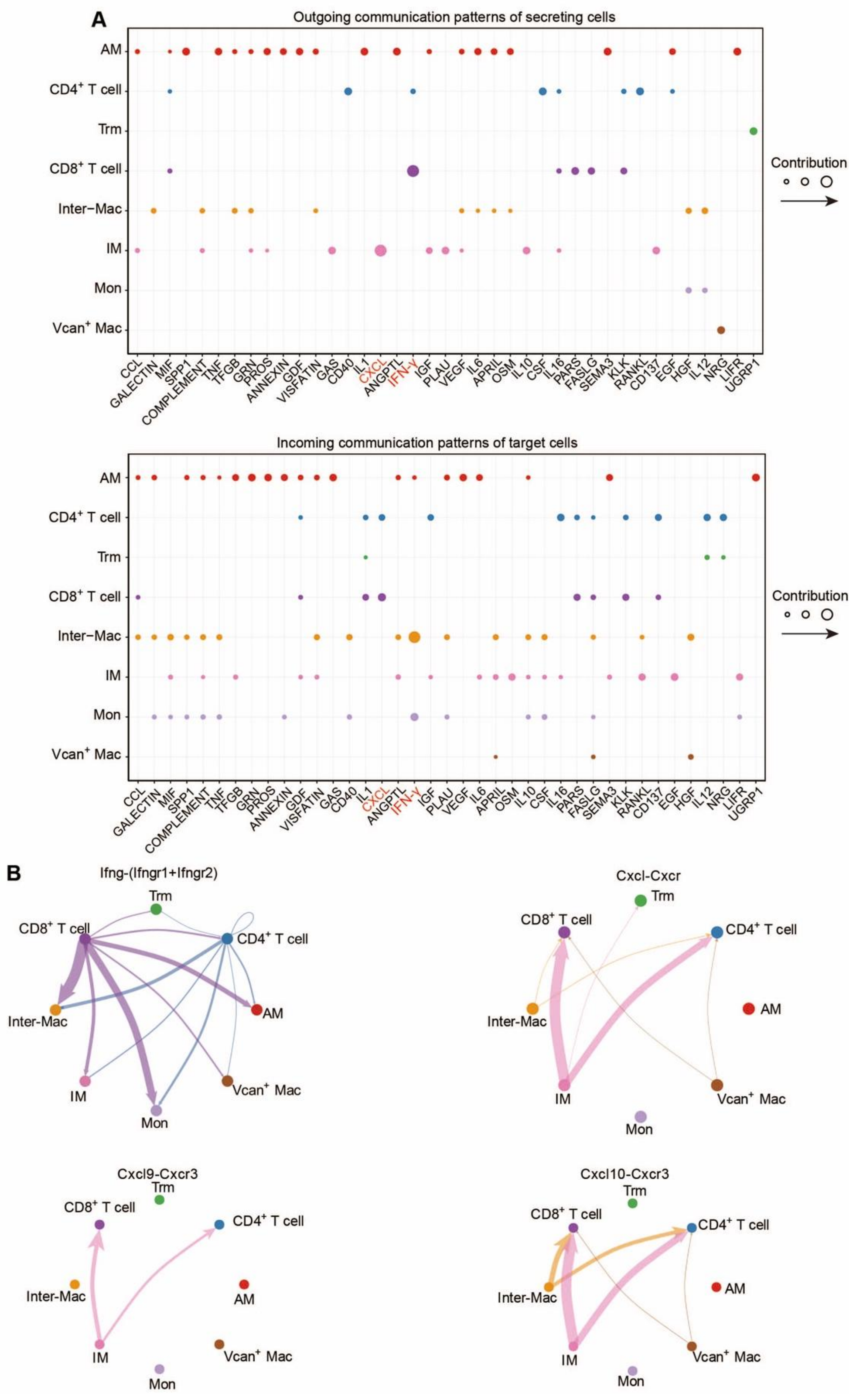

Figure S12: Computationally predicted analysis of macrophage-T cell crosstalk in lungs from *Foxp3-DTR-GFP* mice. (A) The dot plots depicted secretory cell-derived outgoing signaling patterns and target cell-directed incoming signaling

patterns in murine lungs. Dot sizes correlate with pattern recognition analysis-computed contribution scores, where enlargement reflects both elevated scores and heightened signaling pathway enrichment within respective cellular subpopulations. **(B)** The circular plots illustrated the inferred macrophage-T cell communication network in murine lung tissues, highlighting two directional signaling axes: IFN-γ signaling from T cells to macrophages and CXCL9/10-CXCR3 signaling from macrophages to T cells.

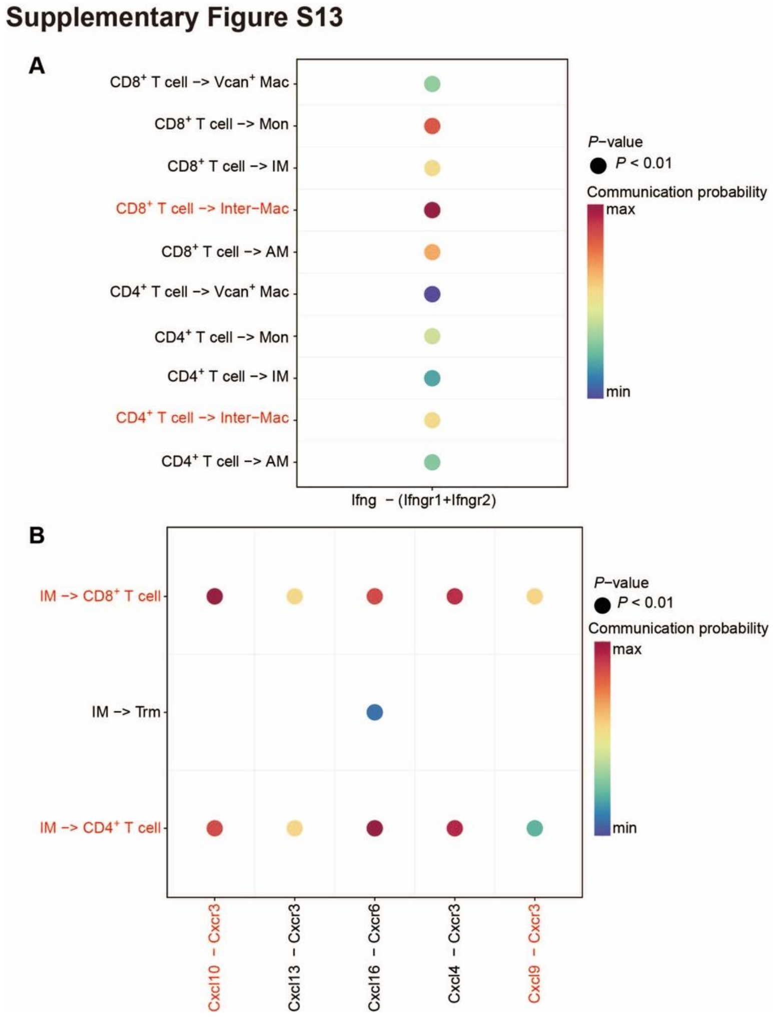

Figure S13: Interaction strength of T cells and macrophages in IFN-γ and CXCL signaling in lungs from *Foxp3-DTR-GFP* mice. The dot plots illustrated interaction strength between T cells and macrophages within the IFN-γ **(A)** and CXCL **(B)** signaling pathways in murine lung tissues, with statistical significance (*P*-values) computed via the R package CellChat.

Supplementary Figure 14

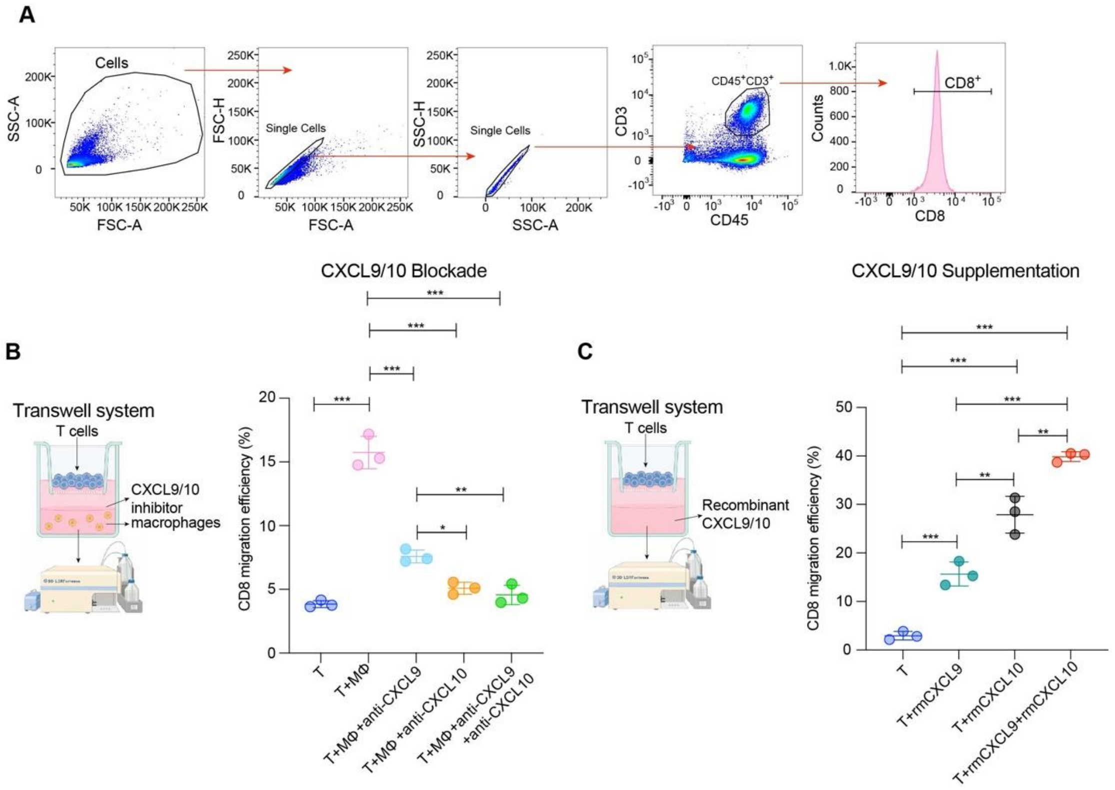

Figure S14: Transwell assay for CD8$^+$ T-cell migration toward macrophages under in vitro perturbation conditions. (A) Flow cytometry gating strategy to quantify migrated murine pulmonary CD8$^+$ T cells after in vitro CXCR3 blockade, CXCL9/10 blockade, or recombinant CXCL9/10 supplementation. At the time of Transwell seeding, the stimulated T-cell population consisted of 56% CD8$^+$ T cells and 38% CD4$^+$ T cells within the CD3$^+$ T cells (approximately a 1.5:1 CD8:CD4 ratio). **(B)** Quantitative analysis of pulmonary CD8$^+$ T-cell migration following CXCL9 and/or CXCL10 blockade of peritoneal macrophages in the lower chamber (n = 3 per group). One-way ANOVA with Tukey's test; significance denoted as $*P < 0.05$, $**P < 0.01$, $***P < 0.001$. **(C)** Quantitative analysis of pulmonary CD8$^+$ T-cell migration following recombinant CXCL9 and/or CXCL10 supplementation in the lower chamber (n = 3 per group). One-way ANOVA with Tukey's test; significance denoted as $**P < 0.01$, $***P < 0.001$.

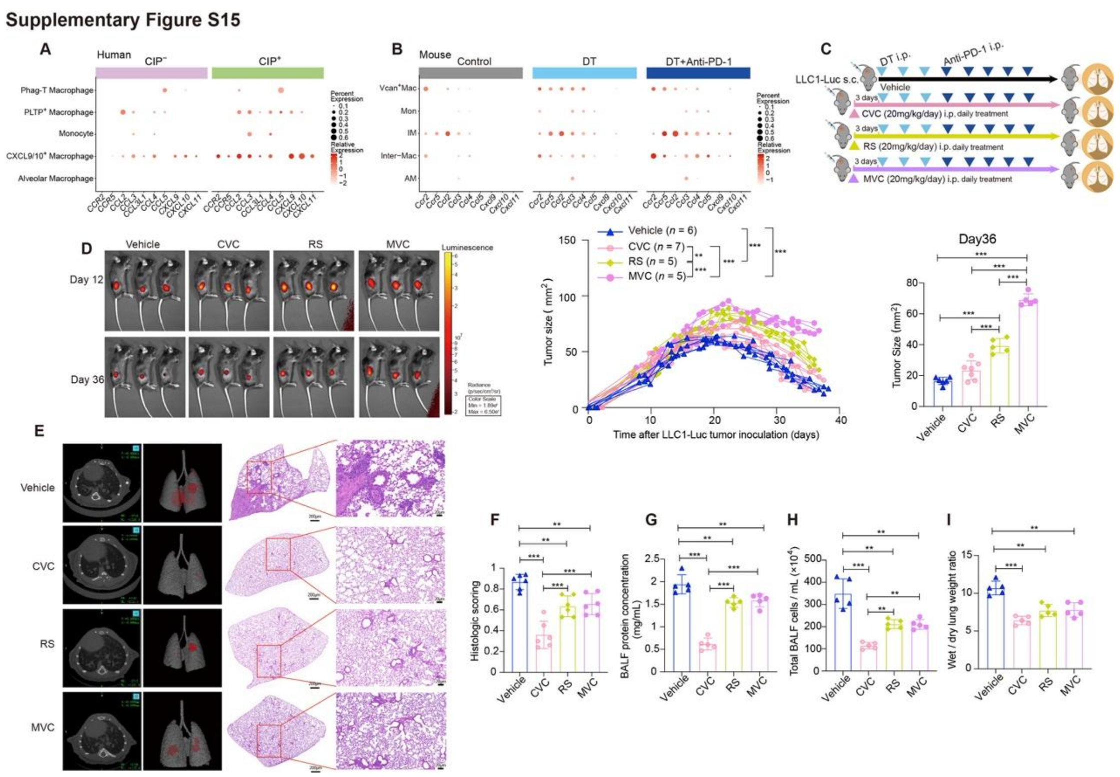

Figure S15: CCR2-CCL2 predominates over the CCR5 axis in pathogenic myeloid recruitment, and dual CCR2/CCR5 blockade provides superior protection in the DT + anti-PD-1 model. (A) Dot plot of CCR2/CCR5, their major ligands (CCL2, CCL3/4), and CXCR3 ligands (CXCL9/10/11) across myeloid subsets in human BALF scRNA-seq, comparing CIP$^-$ and CIP$^+$ samples. Dot size indicates the percentage of expressing cells and color indicates relative expression. **(B)** Dot plot of the same receptor/ligand modules in mouse lung scRNA-seq myeloid clusters across Control, DT, and DT + anti-PD-1 groups. Dot size indicates the percentage of expressing cells and color indicates relative expression. **(C)** Schematic of the pharmacological intervention study in LLC1-Luc–bearing *Foxp3-DTR-GFP* mice treated with DT and anti-PD-1, followed by daily i.p. administration of vehicle, cenicriviroc (CVC; dual CCR2/CCR5 antagonist), RS102895 (RS; CCR2 antagonist), or maraviroc (MVC; CCR5 antagonist) (20 mg/kg/day). **(D)** Representative in vivo imaging (left) of LLC1-Luc tumor burden at the indicated time points across treatment groups. Tumor growth curves (middle) of LLC1-Luc tumors (n = 5-7 per group). Two-way ANOVA with Dunnett’s multiple comparisons test was used. Quantification of tumor sizes on day 36 across treatment groups (right). Individual

data points were shown in the bar graph (n = 5-7 per group). One-way ANOVA with Tukey's multiple-comparisons test was used; significance denoted as $^{**}P < 0.01$, $^{***}P < 0.001$. **(E-I)** Assessment of lung injury. **(E)** Representative micro-CT images (left), 3D-reconstructed lung tissue (gray) and inflammatory foci (red) (middle), and H&E-stained inflated lung sections (right) from LLC1-Luc-bearing mice across treatment groups. Magnification: ×4 and ×40. Scale bars: 200 μm and 20 μm. **(F)** Semi-quantitative histologic scoring of lung pathology. **(G)** BALF protein concentration. **(H)** Total BALF cell counts. **(I)** Wet-to-dry lung weight ratio. $n = 5$ per group. One-way ANOVA with Tukey's multiple comparisons test was used; significance denoted as $^{**}P < 0.01$, $^{***}P < 0.001$.

Supplementary Figure S16

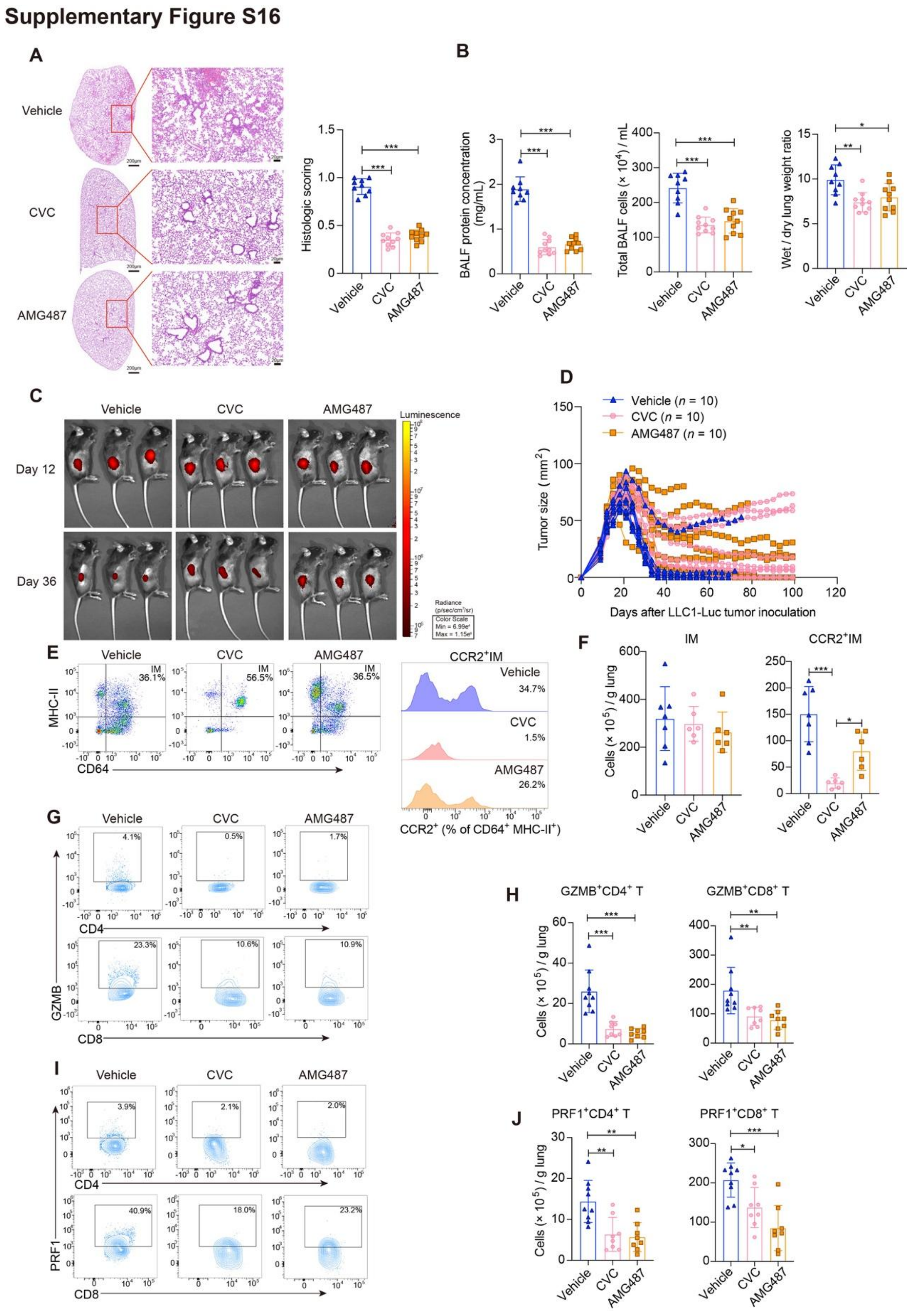

355

356 **Figure S16: CVC and CXCR3 antagonist AMG487 did not affect therapeutic**

357 **efficacy in immunotherapeutic tumors but reduced cytotoxic effector molecule**

358 **expression in lung T cells of CIP mouse models compared with vehicle. (A)**

359 Representative H&E-stained inflated lung sections (left) from LLC1-Luc-bearing

mice across treatment groups. Scale bars: 200 μm and 20 μm. Semi-quantitative histologic scoring of lung pathology (right). **(B)** BALF protein concentration, total BALF cell counts, and Wet / dry lung weight ratio of LLC1-Luc-bearing mice across treatment groups (*n* = 9-10 per group). **(C)** In vivo imaging of LLC1-Luc tumors at the indicated time points across treatment groups. **(D)** Tumor growth curves. CVC and AMG487 treatment did not affect the therapeutic efficacy in immunotherapeutic tumors (DT + anti-PD-1). **(E-F)** Representative images **(E)** and quantification **(F)** of pulmonary interstitial macrophages (IMs: CD11c^{-}CD11b^{+}CD64^{+}MHC-II^{+}) and CCR2^{+} IMs, gated on live CD45^{+} cells. Data from 2 independent experiments (*n* = 6-7 per group). **(G-J)** Representative images (**G**, **I**) and quantification (**H**, **J**) of pulmonary GZMB^{+}CD4^{+} T cells, GZMB^{+}CD8^{+} T cells, PRF1^{+}CD4^{+} T cells, and PRF1^{+}CD8^{+} T cells, gated on live CD45^{+}CD3^{+} cells. Data from 3 independent experiments (*n* = 8 per group). One-way ANOVA with Tukey's test was used; significance levels: *$P < 0.05$, **$P < 0.01$, ***$P < 0.001$.
